# Expectations and human rights protection: the case of Vietnam during COVID-19

**DOI:** 10.12688/wellcomeopenres.18972.1

**Published:** 2023-03-31

**Authors:** Hai Doan, Jing-Bao Nie

**Affiliations:** 1 Bioethics Centre, University of Otago, Dunedin North,, Dunedin, 9016, New Zealand

**Keywords:** expectations; expect; human rights; COVID-19 pandemic; emergency powers; Vietnam; the state of exception.

## Abstract

**Background:** A key part of the complexity of actual social life lies in the fact that it operates on a large web of not only prescribed norms and rules, but also explicit and especially implicit expectations which constantly interact with each other.

**Methods:** This paper offers the first in-depth study on ‘expectations’ in bioethical and health law studies, taking Vietnamese human rights issues in response to coronavirus disease (COVID-19) as illustrations. It defines ‘expectations’ as normative imaginaries that can induce or guide actions and inactions at individual and collective levels.

**Results:** The study suggests that ‘expectations’ is a fruitful concept for bioethical and health law studies because it helps better understand the complexity of interactions in society and patterns of thinking and acting of actors in real-world contexts. Studying expectations also has implications for how human rights and other ethical norms can be better realized in practice. From applying the concept of ‘expectations’ to study human rights issues in Vietnam, this paper also argues that for better protection of human rights, there must be changes concerning expectations of involved parties.

**Conclusions:** Changes in expectations in turn call for changes in stimulus, knowledge, praxis, polices, laws and institutions. Meanwhile, checks and balances mechanisms must effectively constrain the materialization of undue expectations.

## 1. Introduction

Some scholars expect that local actors can help to check the power of central government, thus improving rights protection ^1^. Local actors have been expected to be far better than the central government in controlling and preventing the pandemic ^2^ as they could calibrate policies to local needs, conditions, and preferences ^3^. Such expectations justified the delegation of power – the process in which higher authorities delegate power to lower ones to make and implement public health measures. Such delegation of power fermented conditions for unbounded powers of local officials, thus leading to abuses of powers and possible violations of rights ^4^. Scholars expect that resorting to emergency powers - or more precisely the declaration of resorting to emergency powers – could be a solution for human rights issues amidst the situation. But why did scholars expect this?

It appears that underneath many propositions or actions, there might have been certain expectations. Real-world society is more complex than any theories suggest. A key part of the complexity of actual social life lies in the fact that it operates on a large web of not only prescribed norms and rules but also explicit and especially implicit expectations which constantly interact with each other.

This paper offers the first in-depth study on ‘expectations’ in bioethical and health law studies, taking Vietnamese human rights issues in response to coronavirus disease (COVID-19) as illustrations. It defines ‘expectations’ as normative imaginaries that can induce or guide actions and inactions. It suggests that ‘expectations’ is a fruitful concept for bioethical and health law studies because it helps better understand the complexity of interactions in society and patterns of thinking and acting of actors in real-world contexts, which have implications for how human rights and other ethical norms can be better realized in practice. The second section links human rights issues to ‘expectations’. The third section studies ‘expectations’ as a concept. The fourth section studies interactions between expectations. The fifth section applies the concept of expectations to two human rights issues in Vietnam: (i) advocacies for resorting to emergency powers; and (ii) human rights violations committed by local officials.

## 2. Human rights and expectations

The COVID-19 pandemic can be labelled as an emergency, a state of exception. Since the times of ancient Rome, the state of exception has been a fertile ground for abuses of powers and violations of human dignity and rights ^5^. In Vietnam, with expectations that local actors are better than the central government in controlling and preventing the pandemic ^6^, the delegation of power was implemented nationwide. Following the delegation of power, local (provincial, municipal, commune) governments established checkpoints in their ‘own territories’ and invented significantly divergent rules for their own ‘fortresses’: in some localities, entry of outsiders (people who do not reside there) was banned; in some other places, people were required to test at checkpoints (all other test results, even those provided by previous checkpoints within the previous few seconds, would not be accepted). Unbounded powers soon materialized in abuses of powers and possible violations of rights ^7^. It is reported that local officials have locked citizens' houses, worrying that without such ‘lock-up’, citizens might go out and spread the disease. Tellingly, it is reported in a case – I may call the “ *Bread is not Food*” case - officials first ‘taught’ a citizen that Directive 16 ^8^ only allowed citizens to go out for essential purposes, while buying food was not; such officials then changed the argument and claimed that “bread is not food”. ^9^ In another instance, citizens were coerced by force to take a COVID-19 test administered by public officers. ^10^ These instances cast doubt on the expectations of Tom Ginsburg and Mila Versteeg that local actors can help to check the power of the central government ^11^. Vũ Công Giao and Đào Trí Úc – two Vietnamese constitutional scholars - recognized the awkwardness of such divergences and possible human rights violations. ^12^ They expected that resorting to emergency powers - or more precisely the declaration of resorting to emergency powers – could be a solution ^13^. Their position perhaps captures the view of the wider Vietnamese academia: scholars welcomed the declaration of emergency and initiated proposals to make this possible ^14^. However, it is unclear why emergency powers are expected to be of any help. Notwithstanding, there is a more telling observation: some violations are ridiculously unreasonable. How can we expect a person having full cognitive and perceptive capacity to claim that bread is not food –not just a person but groups of individuals? What did officials expect in committing illegal and unethical acts?

What do the above instances suggest? Perhaps, most observable, in all instances above, there are certain expectations underneath many propositions or actions. The second observation, which can even be a cliché to some people, is that real-world society is complex. Should there be only one moral truth or some rational and virtuous choices which are all opted for by humanity, and people obey high ethical principles or fair legal rules, life would be easy. As stated in the Introduction and as I will illustrate later, various explicit and implicit – sometimes irrational - expectations which constantly interact with each other are responsible for the complexity.

‘Expectations’ is a complex phenomenon that plays a significant role in the shaping and operation of social life. Except for someone who loses the capacity of cognition and perception entirely, everyone has their own expectations for themselves or for others (other people, other groups of people, institutions, or events). Expectations not only represent innate human tendencies, good and bad, but also embed social experiences. Expectations not only respond to explicit messages but also to nuanced and subtle contextual backgrounds. Expectations both reflect on past experiences and represent the present and future speculations. For that reason, ‘expectations’ has long been an important concept for many intellectual fields and theories, which can offer explanations for a range of social phenomena and issues. In the ‘expectancy theory of motivation’ in organizational behaviour, expectations, or expectancy - the *subjective probability* of the attainment of rewards - are *incentives* that motivate employees’ job performance ^15^. In the ‘expectancy violations theory’, expectations refer to *social norms* and *judgements concerning common patterns of behaviours in a given society that actors are expected to follow* ^16^ *,* which impact the attitude of individuals vis-à-vis actions of other individuals and those individuals. In sociology, according to the ‘Expectation states theory’ ^17^, expectations refer to some kinds of *practical beliefs*, i.e., beliefs about practical issues, and even *stereotypes,* which form status hierarchies ^18^ and social inequality ^19^. In science and technological sociology, expectations refer to *wishful enactments of a desired future* ^20^, underlined bold technological advancements.

‘Expectations’ is a core concept in economics and shows its intriguing significance therein. Classical economics claims that expectations are among the factors that drive decisions. Classical economics articulates simply that expectations about the future may affect both demand and supply for a good or service today ^21^. But let’s imagine a case in which the price of fuel is expected to increase tomorrow. Per classical economics’ accounts, many people should buy fuel today. But if many people are that simply rational, running to buy and even hoard fuel, consequently, due to high demand, they must wait in a long queue and some people may have to leave without having fuel (due to shortages). On the contrary, if many consumers expect that many rational people shall run to hoard fuel and decide not to hurriedly buy fuel as their car’s fuel tank is a quarter full, but the fact is unlike what they expected, they may lose a chance of buying less expensive fuel. Indeed, Keynes recognized that a practical decision would not entertain a single undoubting expectation, but *several hypothetical expectations,* held with *varying degrees of probability and definiteness* ^22^. According to Keynes, our own preferences are not as important as the anticipation of *“what average opinion expects average opinion to be”* ^23^, i.e., expectations of an individual about the market are expectations of the average expectation of the market which is the average of the sum of individuals’ expectations. Keynes also recognized that though expectations would be shaped partly by knowledge and creativity, they could be unstable ^24^ and sometimes irrational, influenced by *‘animal spirits’* ^25^. 

Likewise, ‘expectations’ should matter for bioethical and legal studies. Practical ethics, such as bioethics, and law have common aims, among which are to address certain issues or cases within given settings, to assist and guide decision-making in certain cases or issues, to determine what courses of actions subjects (humans, entities, and institutions) should, can, or must implement in particular contexts, and to promote social changes. For these aims, practical ethics and law might want and should need to know and predict how subjects may act or want to act, and why – sometimes with sympathy, sometimes not. Indeed, that is what bioethics and law have been doing. Since the age of ancient Rome, law has attempted to regulate human behaviours. Roman law attempted to expect various typical issues that could arise in a society, for example, patrimony, testation, delict, to provide normative guidance or orders. Similarities could be observed in the Sino sphere. Likewise, since its conception, bioethics – originally medical ethics - has made expectations about various health-related issues. In this vein, as various propositions or actions are motivated by certain explicit and implicit expectations of respective actors, bioethical and legal studies might attempt to understand what expectations of subjects form certain beliefs or conducts of these subjects or else. As a result, though ‘expectations’ as a concept might not yet have occupied a decent position in bioethical and legal studies as in other fields, it is a significant concept to address the complexity in the patterns of thinking and acting of subjects, institutions, and society and thus should have a place in bioethical and legal studies.

As I will demonstrate in greater detail in Section 5, some contested human rights issues in Vietnam can be understood through the concept of expectations. Meanwhile, contested human rights issues in Vietnam illustrate the complexity of actual social life due to various expectations and the significance of the concept of ‘expectations’ in bioethical and health law studies.

## 3. What are expectations?

### 3.1. Definition of expectations

‘Expect’ or ‘expectation’ are words used popularly in daily life. Various learnt fields and theories refer to them conventionally in light of one or other meanings the purpose of such theories or fields. Here this paper attempts to define ‘expectations’ for the sake of this paper and relevant studies. The definition of ‘expectations’ provided by this section is not by any means impact previous understandings of expectations, nor imply that any other definitions are incorrect or so, nor warn against any attempts to develop different definitions of expectations.

Generally, dictionaries for English users define ‘expectation’ or ‘expect’ ^26^ with the following meanings ^27^:

1) A belief, or to think or believe, something will happen ^28^
2) A belief or to think that someone should behave in a particular way or do a particular thing ^29^ or to consider reasonable, due, or necessary ^30^ or to consider bound in duty, responsibility or obligated ^31^.
3) To suppose/a supposition ^32^
4) To await/ awaiting ^33^.

Meaning (2) seems to suggest that expect/expectation may appear as a counter-partner of duty or obligation, but in scrutinizing examples offered by dictionaries, it is not always the case. Examples provided by dictionaries are as follows:

It is reasonable to expect changes in the way we work ^34^. (1)
No one has a right to expect good results without working hard ^35^. (2)
These are the high standards that hotel guests have come to expect ^36^. (3)
He's still getting over his illness, so it's unrealistic to expect too much from him ^37^ (4).
Are you clear what is expected of you? ^38^ (5)
You can't reasonably expect people to pay such high taxes ^39^ (6).
We are expected to work on Saturdays ^40^ (7).
I expect to be paid promptly for the work ^41^ (8).
They expect you to pay your bills ^42^ (9)

From (1) – (3), the sense of legal or moral obligation or responsibility or duty is vague ^43^.

For (4) and (5), subjective expectations from one side need not express a clear sense of being bound by duty, being obliged or obligated, being mandated, or being incurred responsibility from the other side. The other side may not be aware of or may dismiss such expectations.

For (6) and (7), there are contradictions in the sense of being bound. In (6) there can be an obligation under the law which can be irrational or morally invalid. It is the opposite in (7), a feeling of being morally obliged to work extra time can be contrary to the law.

For (8) and (9), the actions at stake can be negotiated, subject to various contextual factors without implying moral or legal wrongs. Consequently, these examples might suggest that certain actions are desired or looked for (irrespective of who carries out such actions).

It would follow from what has been demonstrated that ‘expect’ and ‘expectations’ can also be understood as *“to look for”* or *“to desire”* or *“a desire or an ambition*” and *“to hope”* or *“a hope”*. As a result, ‘expects’ or ‘expectations” do not refer to mere ‘acts of believing’ or ‘beliefs’ in a neutral sense, but ‘to believe’ with or ‘beliefs’ having normative propositions or judgements. 

In brief, this paper defines ‘expectations’ as *normative imaginaries that can induce or guide actions and inactions (generally ‘actions’).*

### 3.2. Objects of expectations

There are several objects of expectations:

- Expectations of a self upon that self.
- Expectations of a self upon other individuals or/and entities (groups, states…) (in short selves). Throughout the paper, I shall use the term ‘selves’. The reason for which I use the term ‘selves’ instead of ‘individuals’ or ‘people’ is that for many entities that are collections of individuals and other entities, (former) entities have their own lives, capabilities, and expectations distinguished from those of their constituents. This can be observed with corporations or states.
- Expectations of selves upon a self.
- Expectations of selves upon other selves,
- Expectations of a self or selves (hereinafter ‘selves’) as to an event or outcome or manifestation (in general ‘event’): Such an event may be driven by selves or just by beyond-control factors, i.e., forces of nature and logic of the universe, e.g., something such as the cause-effect relationship or physical logic underlying the universe. In the cases in which an event is driven by selves, to say ‘an event’ is to stress that what is expected is that event, as opposed to the conduct or characteristics of the selves bringing about such an event. Such expectations may be instrumental (i.e., selves are means to achieve the end) but not necessarily (selves may not concern who brings about that event or how such an event could be brought about).

### 3.3. Some key characteristics of expectations

***3.3.1. The variety***

*First,* expectations vary among selves. All selves have their own expectations, drawing from not only selves’ personalities, past experiences, skills, knowledge, and confidence but also the purposes of these, contexts (i.e., social interactions and expressions), and components of selves.

*Second,* expectations of selves may change from time to time, sometimes just in an instant.

*Third,* expectations are various in terms of issues, contents, and the associated degree of desirability. Expectations are not only about ‘trust/believe or not’. One person can be untrustworthy; however, another person may still have certain positive expectations for them (not to say negative expectations e.g., that the former person may not keep a promise). This shall be illustrated in greater detail in Section 5.

*Fourth,* selves may have various expectations at the same time for themselves or others. Such expectations may be even in sharp contradictions between themselves.

*Fifth,* expectations are various in terms of certainty.

***3.3.2. The rational vagueness***

Expectations can be rationally vague. In saying so, this does not mean that expectations are neither rational nor irrational, but that they may receive a continuum of values with ‘rational’ and ‘irrational’ at polar, depending on various factors. Whether one’s expectations are rational depends not only upon subjective assessments of that given subject but also on “counter-expectations” ^44^ and assessments of others. Keynes is right that rational expectations are only rational as long as they can expect others’ expectations even if the latter can be irrational. The rationality of expectations is also contingent upon uncontrollable factors.

Expectations are also rationally vague in the sense that it is trans-factual: it is not confined to concrete situations or ubiquitous orthodox beliefs or the necessity of clarity of evidence, i.e., they can be daydream, both in the positive and negative sense. ‘Unrealistic’ expectations may sometimes result in unimaginable outcomes, good or bad, or help to achieve all or some unrealistic targets but sometimes are counterintuitive. The iron-willed ‘Zero Covid’ and its unshakable implementation, as illustrated in Section 5, can be observed as an example of ‘unrealistic’ expectations.

***3.3.3. Moral vagueness***

3.3.3.1. The moral value of expectations

Expectations are morally vague. Expectations of selves can be clearly moral or immoral, or morally uncertain, e.g., in case of dilemmas or in times of uncertainty or simply not attending to morality at all. For example, a German soldier expecting to follow orders of superiors or discipline of an army carried Jews to extermination camps without expecting moral consequences or even with expectations of vices. Specifically to human rights violations in Vietnam during COVID-19, Section 5 will illustrate how in some cases, violations were made with immoral expectations in some cases and other cases with morally uncertain expectations.

3.3.3.2. The ethical frame forming expectations

The ethical frame forming expectations is also vague. Expectations can be consequential (inclination to rewards, pleasure or aversion of pains or punishment or risks or disrepute or losses) or deontological or just authority-oriented or herd-oriented (i.e., unidentified moral values). Stanley Milgram has two experiments. The first is *‘Obedience to Authority’,* suggesting that (i) ordinary people may expect themselves to follow orders. As a result, without any particular hostility [and perhaps rewards], people can become agents in a terrible destructive process ^45^; (ii) ordinary people may expect authorities to be truthful. There are explanatory accounts for the behaviours of ‘teachers’ as such behaviours were due to expectations that ‘learners’ would not be permanently damaged, formed upon reassurance from the experimenter ^46^. Notwithstanding, such expectations, if existed at all, would neither be rational nor ethical. People without physics knowledge can still expect that 300-450V would be harmful and even deathly. Non-harm expectations are also not supported by contextual factors (screams of learners); (iii) wrongdoings, immoralities, and harms can predicate upon expectations of a singular fantastic cause while overlooking related surrounding possible ethical concerns and peripheralizing other interests ^47^. There are many variants of ‘Obedience to Authority’ with the willingness to go to the end (the administer of 450V electric shock) varies greatly ^48^. It is suggested that the willingness to go to the end can be reduced significantly should participants sense countervailing considerations ^49^. The second experiment known as the glaze-following effect demonstrates that the probability of a person following the stimulus group’s action increased with the size of the stimulus group ^50^. It suggests that expectations can be formed in response to meaningless behaviours of others.

Conversely, expectations play a role in determining decisions or actions through expecting moral values of these decisions or actions once implemented. For practical purposes, let’s take the debate on vaccine mandates as an example. Proponents of vaccine mandate campaigns may expect a scenario in which in the absence of wide coverage of vaccination, millions of people may die excessively and in the absence of a vaccine mandate, a significant portion of the population may refuse vaccination. Those expectations give grounds for expectations for moral values of vaccine mandate, especially in the absence of free consent. Opponents of mandatory vaccination expect that people are rational and shall choose what is optimal for them or that the vaccine is ineffective or even defective, recalling the ‘Cutter Incidence’. ^51^

***3.3.4. The ambiguousness themselves***

Expectations are ambiguous in themselves. First, as selves may have various expectations at a given time or from time to time, sometimes they are not clear what they expect for or expect for the most. These expectations may even run counter to each other. Second, as a corollary of the first and/or due to the complexity of contextual factors, the decoded messages conveyed by the signalling expectations are vague, thus making ‘counter-expectations’ formed upon such messages vague: A self may receive various messages that they try to interpret, and the interpretation process results in various contradictory imaginaries of signalling expectations and thus leading to contradictory counter-expectations. Sometimes, this vagueness of counter-expectations is accidental or due to poor interpretation skills, but sometimes it is intentionally made by senders.

***3.3.5. The contextual impacts***

Not only internal traits, e.g., genes, brain, and nerves but contextual factors, i.e., social roles, social interactions, or social expressions, play a significant role in shaping expectations. As I will illustrate in more detail in Section 5, in response to the same phenomenon, legal scholars and officials may form different expectations, the same can be observed for legal scholars receiving different training. Meanwhile, certain commonalities in patterns of expectations of individual subjects belonging to a social group can be observed. For example, while legal scholars attend to human rights issues, scholars from democratic jurisdictions such as Tom Ginsburg and Mila Versteeg may have expectations that local authorities protect the rights of citizens from violations of the central government. Scholars from jurisdictions in which local authorities are notorious for abuse of powers may not have and are not confident in the aforementioned expectation.

## 4. Interactions of expectations

Because society is a web of interactions between selves, and between selves and institutions, expectations do not exist in a vacuum or singularity but in a web. It is to say that expectations may direct or respond to other expectations.

### 4.1. Signalling expectations and counter-expectations

As expectations are a subjective state of mind, selves need not be triggered (by other expectations – I call ‘signalling expectations’ - of other selves directed at the former selves or by stimulus) to form expectations. However, expectations are also formed in response to signalling expectations - I call expectations formed in response to signalling expectations ‘counter-expectations’. However, counter-expectations are not direct products of or having any causal relationship with signalling expectations. Rather, counter-expectations are products of subjective interpretations or imaginaries of meanings of a complex web of factors, among which signalling expectations are a factor. As a result, expectations to respond to expectations are more accurately framed as expectations to respond to presumed signalling expectations. Such interpretations of messages upon which counter-expectations rely and form are subjective and can be false - if there are any signalling expectations at all.

### 4.2. States of interactions

***4.2.1. Meeting expectations***

Selves may try to meet expectations of other selves, especially those of higher authority. In this pattern, presumed signalling expectations are interpreted as normative and authoritative. Selves assuming the presumed signalling expectations are directed toward them may have a sense of being bound (though sometimes this sense can be subtle, and there is no legal or moral ground supporting that sense). There are two sub-categories: (i) intentional meeting expectations; (ii) de facto meeting expectations.

(i) *intentional meeting expectations:* Intentional meeting expectations refers to situations in which selves intend to meet the interpretations of presumed signalling expectations. For example, in psychology, there is a phenomenon called *demand characteristics* in which participants (of experiments), perhaps upon expectations that they will somehow be evaluated, subconsciously change their attitude and behaviour to match [interpretations of] expectations of the experimenter or to appear more socially or morally responsible ^52^.
(ii) *de facto meeting expectations:* if intentional meeting expectations acts neatly fit (though it is not necessarily perfectly fit and what is the degree of “neatly” is vague) with real signalling expectations, following accurate interpretations of signalling expectations in whole or in substantial part, such acts are also de facto meet signalling expectations.

De facto meeting expectations acts of selves may make the selves sending signalling expectations feel that their expectations are met. In case signalling expectations are met in light of actions which are derived from counter-expectations which are thought to be formed upon accurate interpretations of signalling expectations, but in fact, upon misinterpretations, signalling expectations and counter-expectations are reinforcing expectations. Expectations are also reinforcing in cases where signalling expectations [I call expectations (i)] are entirely missed and actions that please such expectations (i) are derived from independently considered expectations [I call expectations (ii)] (e.g., in directing at different sets of expectations [I call expectations (ii) or (iii)]. Expectations (ii) are not counter expectations of expectations (i), rather expectations (i), (ii), and possibly (iii) are reinforcing.

***4.2.2. Fulfilling expectations***

There are instances in which expectations are fulfilled but not met. The difference between ‘fulfilling’ and ‘meeting’ is that for ‘fulfilling’ the selves acting do not feel signalling expectations are positive or authoritative.

***4.2.3. Meeting or fulfilling unidentified expectations***

The famous Hawthorne effect suggests that people tend to change their behaviour should they expect that they are being observed ^53^. In these instances, selves may form certain expectations in response to unidentified expectations of unidentified other selves, social norms, or whatever.

***4.2.4. Failing expectations***

Falling expectations refer to two scenarios. The first is that signalling expectations are missed or misread, consequently, selves act in a manner that displeases the signalling expectations. The second is that though signalling expectations are interpreted accurately in total or in substantial part, attempts to achieve what is expected or satisfy needs behind the signalling expectations fail. Often, failing expectations may result in punishments or unfavoured treatments from selves emancipating signalling expectations.

***4.2.5. Evading and resisting expectations***

Sometimes, selves may attempt to evade or resist expectations directed upon them should there be conflicts or clashes in expectations between the former selves and selves sending signalling expectations or between expectations of the latter with any third parties who are more mattered to the former. The “bank wiring room experiments” suggest that despite payment incentives, productivity decreased due to suspicious expectations of workers that their productivity may have been boosted to justify firing some of the workers later ^54^.

***4.2.6. Beyond expectations***

Sometimes, actions are beyond expectations. There are several layers of meanings for ‘beyond expectations’. The standard understanding of the phrase provided by some dictionaries is that “act is more than people thought would be the case”, usually with positive connotations. There are other layers as well. First, cases in which consequences or outcomes of actions can be either better or worse than the initial expectations (desires) of the selves implementing actions. Second, cases in which expectations are directed on selves (directed selves) by other selves (directing selves), and it is either that the actions of directed selves are deviating from or are excessive or insufficient than expectations of directing selves. Third, cases in which consequences or outcomes of expectations of directing selves are either significantly better or worse than first planned by directing selves. Forth, scenarios in which events selves strongly refuse to believe or wish against ultimately take place.

## 5. Applying ‘expectations’ to study human rights issues in Vietnam

After studying conceptual issues of ‘expectations’, this section applies the concept to two human rights issues in Vietnam: (i) advocacies for resorting to emergency powers; (ii) human rights violations committed by local officials. These two issues show specifically how various contradictory expectations have been formed and have been interacting with each other within the concrete social Vietnamese conditions and consequently have shaped or advanced Vietnamese society. This also illustrates the theoretical points I made in Section 3 and Section 4, specifically, expectations are formed due to complex interactions of social-influenced factors and innate tendencies; expectations, in many instances, can be counterintuitive, irrational, and even immoral; and expectations constantly interact with each other.

### 5.1. Expectations and human rights protection in Vietnam (1): Calls for emergency powers

Perhaps, expectations for emergency powers are drawn from (i) instrumental rationale and (ii) normative concerns for human rights and the rule of law. These expectations link to not only the transformation in Vietnam happening over the past decade, i.e., societal background, but also the way of thinking of law people, i.e., social experiences of law people.

***5.1.1. Instrumental rationale***

Vietnamese scholars argued that emergency powers would give legal ground for the state to make and implement strong public-health measures that drastically restrict human rights and thus, should the state of emergency not be declared, the legal basis for restrictive measures would be lacking or insufficient ^55^. Put differently, such accounts can be understood as primarily concerning the instrumental rationale of furnishing a legitimate basis for restrictive measures.

However, in reading Vietnamese law, such a view is incomprehensible. In the cloak of the vague term ****‘social distancing’****, lockdown was possible under Directive 16. Moreover, Decision 447/QD-TTg dated April 1, 2020, recognized COVID-19 epidemic as a *class A infectious disease* ^56^. Decision 447/QD-TTg recalled measures provided under the law on Prevention and Control of Contagious Diseases (LPCCD). The law on Prevention and Control of Contagious Diseases is drafted in broad and vague terms that even if Article 54 dealing with a state of emergency would not be invoked, other articles could still be interpreted without any limitations. Pursuant to Article 53 on control of entry into and exit from class-A epidemic zones, measures include

“(i) *restricting* persons and means of transport from entering and leaving epidemic zones…;

(ii) prohibiting transportation from *epidemic zones* of articles, animals, plants, food, and other commodities capable of transmitting the epidemic disease;

…

(iv) *Other necessary measures as prescribed by law.”* [Emphasis]

If interpreting that the ambit ‘epidemic zones’ comprise the whole nation and restriction is equivalent to prohibition (restricting all movements) then a lockdown at the national scale is legally provided and justified. Article 53.1.3. even permits authorities to invent measures that they deem as necessary to prevent and control diseases ^57^. Nothing within the Vietnamese legal system prevents such an approach of interpretation. Also, nothing prevents associated documents, e.g., Directive No 16/CT-TTg and Decision 447/QD-TTg from tracing their validity and even legal effects back to Article 53 of LPCCD. Gustav Radbruch read straight the spirit of legal positivism: a law is a law which is valid because it is a law, and it is a law if, in the general run of cases, it has the power to prevail ^58^, the positive law, secured by legislation and power, takes precedence even when its content is unjust and fails to benefit the people ^59^. However broad and susceptible to being violated the LPCCD is, because the conflict between statute and justice does not “ *reach such an intolerable degree...”* ^60^ *,* such a law is still valid. Perhaps, legal scholars expected the derogation from rights. Notwithstanding, it should be noted that first, the 2013 Constitution still does not make a distinction between restricting rights and derogation from rights and under Article 14 of the 2013 Constitution, all rights (including non-derogable rights as provided under the International Covenant on Civil and Political Rights [ICCPR]) can be restricted. It would follow that from a pure domestic dimension, again, nothing prevents even the restriction of non-derogable rights ^61^. Second, the LPCCD and associated documents, with blanket bans of activities and authorizations of every measure of agencies, de facto derogated many rights ^62^ and even possibly violated non-derogable rights, e.g., the prohibition of torture, cruel, inhuman, or degrading treatment or punishment (Article 7 of ICCPR) ^63^.

If so, why do scholars expect emergency powers to furnish a basis for drastically restrictive measures? These expectations derive from the phenomenon that I may call ‘laws and legal regimes shopping’. Though it is beyond doubt that before Đổi Mới – the period when Vietnam decided to implement liberal economic policy and open to the world - there had been laws, if not to say a lot of laws ^64^, in abolishing private ownerships and rehabilitation capitalists, the Vietnam Government blew out the rules, branches of laws, legal regimes, and mechanisms dealing with private legal relationships. With Đổi Mới, the state had to re-establish or build as quickly as it could many fields of laws, particularly by means of transplanting laws of many different countries into its legal system ^65^. However, while there have been technical drafting issues, the party-state also has been facing opposites: its manifested socialist ideologies (and associated politico-legal order) and the new liberal legal order. As a result, the transplantation has been a mixed process in which the party-state constantly has been refusing to transplant the capitalist legal order entirely. But incompatibilities between various social and legal structures soon come to the foreground, which forces the party-state to adopt new liberal institutions acquiescently and gradually. Put differently, the party-state must simultaneously modify technical errors and conciliate various structures. Meanwhile, the world has also entered a new era of production which yielded more relations to be addressed. This exacerbates the need for more and more laws and regimes and institutions. There is a feeling that the legal system still lacks legal rules, legal regimes, and institutions and as a result legal rules, legal regimes, and institutions still need to be imported or built.

***5.1.2. A positive linkage to human rights protection***

Vũ Công Giao and Đào Trí Úc subtly refer to human rights issues. They claim further that

*“Because this Law was adopted prior to the 2013 Constitution but its provisions are not reflected in that Constitution, it cannot be a legal basis for Directive 16 or Ordinance 2000 to introduce more specific derogations on human rights than the Constitution allows. Further, from an international human rights perspective, Directive 16 does not meet the procedural requirements for a human rights derogation”* ^66^

However, one has a reason to wonder whether declaring a state of emergency and emergency powers positively impacts human rights. It does not, unfortunately. Past experience shows us that with the distinction between rules for ordinary times and states of exception, and in the latter state, rights could be blanketly limited or derogated, the state of exception is a signpost, an excuse for, or prone to violations: there could be no more stark examples than the declarations of Benito Mussolini or Hitler ^67^. Drawing from past saddened experiences, ICCPR provides strict conditions for limitations of rights (known as the Siracusa principles) and the right to avail the derogation of rights of sovereignties (Article 4). The International Covenant on Economic, Social and Cultural Rights (ICESCR) even suggests that the state of emergency cannot suspend rights provided thereof. Unfortunately, today’s concerns are not less than yesterday’s. While requirements provided under Article 4 of ICCPR have rarely been obeyed, the Siracusa principles have been violated in many instances. Concerns for ‘pandemic backsliding’ or the use of the pandemic as a pretext to violate rights and crack down on voices are real ^68^ and have materialized ^69^. Against this backdrop, it should be noted that not only Vietnam, but many other states felt very reluctant and did not invoke state of emergency ^70^.

While it is beyond doubt that human rights are among the central concerns of legal scholars, expectations of emergency powers for the sake of human rights also link to the transformation in human rights regimes. In the past, there was no place for the term “human rights” in Vietnam. Rights were understood as civic rights at best and inseparable from duties: the State only guaranteed civic rights as long as the citizens had fulfilled their duties first ^71^. This position has been still maintained today in the far dogmatic swings in the Party’s line. The 2013 Constitution goes further, recognizing not only human rights as separate from civic rights but also (the disabled version of) the Siracusa principle (Article 14.2), thus inferring that in theory, there should be a strong case and grounds to limit rights (However, there are still miles for Vietnamese jurisprudence to go in the path of human rights protection).

***5.1.3. Better supervision***

Perhaps, expectations for emergency powers can also make sense as expectations for greater supervision ^72^:

“ *As a result, a mutual control mechanism between the legislative, executive, and judicial bodies has been officially constitutionalised (in art 2 of the 2013 Constitution)”* ^73^ and *“Some may argue that these revisions or reforms are meaningless because in Vietnam’s single political party regime, all constitutional institutions … are controlled by the Communist Party, ... However, according to art 4 of the current 2013 Constitution of Vietnam, even if the Communist Party is the leading force of the State and society, the Party’s organisations and members must still operate within the framework of the Constitution and the law…*” ^74^

5.1.3.1. Supervision from the National Assembly

Generally, to constrain the use of emergency powers and prevent potential distractions, most constitutions require the legislative branch to involve in safeguarding against violations ^75^. Notwithstanding, any authorization from the legislative branch does not necessarily embody the form of a declaration of emergency. The legislative branch can vest in the executive further powers to tackle issues which neatly fit the purpose of handling the disease through ordinary legislation - the “legislative model” of crisis governance. ^76^

If so, the Vietnamese National Assembly did authorize the Vietnamese Government much later at the climax of the Delta wave under Resolution no. 30/2021/QH15 dated 28 July 2021, however problematic such authorization is. Notwithstanding, there are several issues with the authorization. *First,* it is a blanket authorization ^77^: without any mechanisms for scrutinizing, the National Assembly authorized not only the Government but local governments to take any measures not yet prescribed by laws ^78^. This should be read within the context that the Government required that local authorities’ measures must be ‘one-level higher’ and ‘one-step earlier’ than the measures of the Government ^79^. *Second,* it is interesting to mention that not only the National Assembly’s Standing Committee and the National Assembly have acted ultra-vires and deviated from the laws - not just authorized “to deviate from the laws” as a scholar claimed. ^80^ The key point to mention is that on matters of significance to the nation, the National Assembly plays a more facilitative role than checks and balances. Such a facilitative role premises in the fact that most of deputies of the National Assembly are members of the Vietnamese Communist Party ( only 14 out of around 500 members are non-Party members) and for such matters of significance to the nation, always there are orientations from Party offices. Indeed, legal documents all recall political documents and speeches. *“Sovereign is he who decides on the state of exception”*, as Carl Schmitt claimed perhaps ^81^?

5.1.3.2. Supervision from the judiciary

Vietnamese scholars attend to supervision from courts as well. However, they also offer a contrasting light: mechanisms for constitutionality review in Vietnam are “little more than a formality” ^82^ and “although Vietnam has an administrative procedure law that allows citizens to sue state agencies and public officials, such lawsuits are often fruitless, partly due to the lack of independence of the court system from the Communist Party and other state agencies ^83^; local administrative courts tend to defend state agencies and public officials because they are considered ‘their own side’ ^84^. Besides, though it is naïve to say that Vietnamese courts cannot de facto decide cases as they want irrespective of law or cannot ‘invent’ norms even in an arbitrary manner, courts are neither enabled to interpret laws nor reasoning ^85^, which in turn indicates that courts cannot interpret documents with the constitution as the touchstone. These accounts may cast plenty of doubts on calls for supervision from courts.

The expectations of scholars may come from transformation concerning the National Assembly and the Judiciary. In the past few years, despite being subtle, the function of the National Assembly to check the Government and bills has improved. Resolution no. 30/2021/QH15 and Resolution no. 268/NQ-UBTVQH15 are stark examples that show attempts to manifest the function of check and authorization from the National Assembly – despite just being symbolistic and expressive. The National Assembly can express its checks role except for matters of significance to the nation which requires orientation from the Party: because of low voting records, some bills were declined. Changes are more observable concerning the judiciary, both in terms of mechanisms and sources of law which are at disposal of judges.

***5.1.4. Manifestation of the rule of law***

Before Đổi Mới, the party-state only recognized socialist legality. Law was just a discipline of the Party and an instrument of the state ^86^. Socialist rule of law and socialist rule of law-based State was adopted in the Seventh Congress of the Party and Resolution 51/2001/NQ-QH10 ^87^. Though scholars of the Party and other scholars may continue to dispute concerning what is the substance of the so-called Socialist Rule of Law-based State, both groups recognize the principle of *‘the supremacy of the Constitution’* (though sometimes, a high-ranking Party official may believe that the Constitution is secondary important after the Party’s Platform); accordingly, all actors must adhere to and act within the boundary of constitution and laws.

In this vein, many calls for emergency power can be understood as a concern with the rhetoric of socialist rule of law of the party-state, i.e., the party-state should be bound by the procedural rules invented by itself, although this manifestation can be symbolistic or counterintuitive. Perhaps, it is also about the manifestation of the sharp distinction between the state of exception and ordinary states, which implies that the rule of law should be more manifested in ordinary states.

### 5.2. Expectations and human rights protections in Vietnam (2): Violations by local officials

After studying scholars’ expectations of emergency powers, I move to a more controversial matter: expectations of officials in committing human rights violations. There are some questions to be addressed:

1. It seems that in Vietnam and elsewhere, statements and rules of central governments – on paper – seem benevolent and promising, but quite paradoxically there are often issues concerning implementation or elaboration from local authorities, why?
2. Why did local officials commit human rights violations, given that some violations seem ridiculously unreasonable?
3. Why did no one in groups of officials warn against or oppose such contested violations?

In this section, I connect contextual accounts and innate tendencies of individuals - agent accounts - to frame analysis.

***5.2.1. Expectations of central government***

Often the central government may make a lot of contrasting statements, some of which address the central government in a neutral position or as a rational and considerate actor. But as mentioned in Section 4, actions of local actors are formed upon their expectations of what the central government expect them - local actors – to do or achieve. Such expectations of local actors rely not exclusively on interpretations of explicit statements but on confidence in unpublicized and hidden messages. Perhaps, this tendency is due to the human instinct that prefers decoding something complex to just reading public statements or the confidence in privileged intelligence over common knowledge. Perhaps, what local actors expect as expectations of the central government for them has much more gravity than what the central government addresses publicly, especially in the case where two messages may clash. “Đoán ý lãnh đạo” – “Guessing what is in the mind of a boss/high-ranked official” - is not a rare phenomenon. Literacy and sensibility of expectations of bosses is the ability to survive in offices, both private and public, at least somewhere. If local actors expect that they shall be reprimanded or punished should some bad news air (e.g., the outbreak or spread of a disease) and should their measures be perceived as insufficiently aggressive, they will make every attempt to prevent such possibilities, even notwithstanding or in contrary to the principle of humanity. This is the case with the tragic “Great Leap Forward Campaign” in which local officials fearful of Anti-Rightist Campaigns left farmers to starve to death, or with repeated concealment of SARS and COVID-19 outbreaks in China. This is not different within the context of Vietnam. Though the Prime Minister interpreted Directive 16 as neither a lockdown nor a traffic ban and warned against divergent, inconsistent, and excessive measures of local authorities, the Government also required that local authorities’ measures must be ‘one-level higher’ and ‘one-step earlier’ than the measures of the Government and that localities, where disease circulated, would be held accountable. As the two messages are somewhat conflicting, how should local officials act? In fact, while officials who violate human rights are usually not dismissed, many officials are dismissed because the disease circulates in their territories.

While the outcomes are the utmost important parameter for performance, officials may also expect that they are expected to express their functions which either directly produce the expected outcomes or shapes the social attitude and behaviours to serve the Government’s political goals or outcomes. The latter has been known as mass mobilization. *“Mass mobilization is mobilizing all the forces of each citizen without leaving a single citizen out, … to perform … the work assigned by the Government…”* ^88^ In addition to propaganda, activities of officials are also a conduit for mass mobilization. Mass mobilization can be positive ^89^ but also oppressive and degrading, especially for those who are recognized as enemies or who do not fit in the expected patterns of behaviours. Mobilization may comprise *rehabilitation, verbal denunciations and accusations, and public humiliation, especially by making someone a case*. Officials might not perceive their acts as violating human rights; instead, they might expect that their actions can *‘educate’* citizens about the policy and civic duties. In the “Bread is not Food” case, despite opposition being recorded by the citizen at stake, the officials still recorded and circulated the video widely for “educational” purposes. In fact, before that event, there had been other videos of the same kind, circulated online by such officials.

***5.2.2. False Consensus: Expectations of consensus from the masses***

Officials may expect consensus from the masses. However, these expectations might be false or biased. According to the famous *“False Consensus Experiment*”, if people hold certain beliefs or judgments, they may tend to expect that the majority of other people may hold the same beliefs or judgments ^90^. Perhaps, in observing how videos recording the scenarios in which police chased people exercising outside near their houses or in their houses’ hallways received wide support from the masses ^91^, officials may expect that they act correctly and shall be supported by the masses?

***5.2.3. Authority and conformity bias***

Milgram’s “Obedience to Authority” experiment illustrates a phenomenon called “authority bias”. Authority bias describes individuals’ tendency to give excessive weight to the opinions and decisions of authorities in the absence of compelling evidence or logical reasoning. There is also another bias influencing decisions known as conformity bias. An experiment by Solomon Asch showed that a large majority of participants conformed to the group at least once though such an answer was incorrect ^92^. Selves may expect authorities and the masses to be benevolent and truthful without self-reflecting. In this vein, selves may expect to follow patterns of actions of the masses and that such patterns of actions are correct. These biases explain how groups of officials may commit human rights violations – even when such violations are ridiculously unreasonable. These biases might be aggravated under real world conditions due to real political hierarchies, authorities, responsibilities, and punishments. For example, according to the principle of “democratic centralism”, decisions of a high authority once made are binding for all subordinates and there are expectations of being disciplined should the latter refuse to follow orders.

***5.2.4. Expectations of manifestation of power***

The *“Stanford prison experiment”* suggests that people in positions of power might be prone to unethical behaviour and show less empathy and concern for the welfare of others ^93^. It follows that we may expect that people with too much power without constrains (checks and balances included) are prone to abusing power and that ‘expectations to express powers’ is a deep veiled evil inside selves. The previous sentence contains two meanings of ‘expectations’: ‘expectations’ as presumptions that something may happen and ‘expectations’ as desires. The latter ‘expectations’- ‘expectations as desires to express powers’ are among behavioural tendencies, expressed under certain conditions. Such tendencies, however, are not beyond the control of wrongdoers. Nor are they a product of exclusively the status of being: for a world where there is one human being, perhaps there shall be no expectations to express powers. Thus, those expectations are a product of society and social interactions. As mentioned earlier, expectations about how the world would be or people would behave (expectations for others) and expectations of a self about that self or what that self can do are reinforcing each other: for a society in which abuse of powers occurs systematically and people, including those holding offices, expect that those who hold offices can abuse powers and get away with it, how can an official refuse to do so? In this vein, understandings and imaginaries of social roles and concrete societal conditions ferment, realize, and aggravate innate vice expectations. Meanwhile, expectations as behavioural tendencies once realized reinforce and construct societal understandings and imaginaries.

In Vietnamese society, from the feudal to colonial periods, there were shared impressions of powerful and vice officials (mandarins) who could abusively manifest their powers to bend the law for their gains and oppress citizens ^94^. Today, expectations for [manifesting] powers are still a part of imaginaries of the social roles of officials ^95^. Before the pandemic, low-level authorities had already abused their powers in limited bureaucratic works ^96^. The pandemic amplified powers possessed by local authorities to an unprecedented degree. Unfortunately, as low-level authorities had never possessed such unbounded powers, they might expect themselves to make all use of these.

***5.2.5. Expectations of good consequences***

After saying many things about ugly tendencies, it is about worthwhile to consider certain positive possibilities. In many instances of human rights violations, officials invoked public health, i.e., the health of the community, and even the health of individuals whose rights were violated, as justifications. In some instances, such justifications could be a pretext, but in some other instances, they represented honest expectations. Such expectations might have been drawn from sharp distinctions - and even an antagonistic relationship - between health and rights - a false dichotomy, perhaps. On the one hand, the central government constantly conveyed messages such as “staying at home is patriotic”, “surely, we will soon defeat the COVID-19 pandemic”, “rigid and harsh restrictions are the only way to eradicate COVID-19 and to win the battle against COVID-19” – why should local authorities doubt such catchphrases? Is it not true that thanks to the “Zero Covid” policy, Vietnam did successfully handle the pandemic early on? Expectations as such of local authorities were not just authority or conformity bias or merely following rules or averting punishments but, in some instances, embodied sincere but staunch expectations: Lockdown was expected to be the silver bullet for COVID-19. Such expectations were channelled to clash with human rights: “try to withstand a bit more and with more aggressive and rigid restrictions, COVID-19 will soon be stamped out”; “without being survived, you cannot have any rights”, some officials and news claimed; consequently, in the name of survival, suspending all rights would be considered as just. However, on a critical analysis, staunch expectations would have been reconsidered: “Is it correct that the relationship between health and rights is antagonistic?”, “Is it correct that the more rigid the measures are, the sooner COVID-19 is stamped out?”. But unfortunately, authority bias might also have been exacerbated by ‘truth bias’ ^97^. For so long, officials have been indoctrinated with the idea that claiming rights is selfish and that individuals claiming rights have ill intentions ^98^. Officials might not expect that not only there is no positive correlation between restricting rights and better health, but health is also one of the rights and good health cannot exist in the absence of full guaranteeing of rights. Ultimately, the claimed purpose of better protecting the health of the population from COVID-19 soon turned counterintuitive: due to psychological and physical suffering and deprivations of all activities, people may be worse at recovering and sections of the population that face the most detrimental impacts are marginalized groups ^99^. Notwithstanding the awareness of the correlation between rights and health, in some instances, under various pressures, officials stand at a fork in the road and struggle to determine what is the right thing to do, instead of feeling satisfactory due to making use of powers.

## Conclusion

This paper studies ‘expectations’ as a concept for bioethical and health law studies. It defines ‘expectations’ as normative imaginaries that can induce or guide actions and inactions. It suggests that ‘expectations’ is a fruitful concept for bioethical and health law studies because it helps better understand the complexity of interactions in society and patterns of thinking and acting of actors in real-world contexts, which have implications for how human rights and other ethical norms can be better realized in practice. 

Issues emerging in Vietnam or elsewhere during the COVID-19 pandemic are not just human rights: a broad range of issues such as how to respond to risks and uncertainties, or how to govern research activities considering the acute needs of controlling the disease has come to the fore. Also, human rights issues are more than just about advocating for resorting to emergency powers and human rights violations committed by local authorities, but include a range of ethical and legal issues surrounding various policy choices such as decentralization or mandatory isolation. These issues can be similarly addressed by applying the concept of ‘expectations’.

Within the scope of this paper, by applying the concept of ‘expectations’ to study advocacies for resorting to emergency powers and human rights violations committed by local authorities in Vietnam, this paper argues that:

*First,* for better protection of human rights, there must be changes concerning expectations of involved parties, e.g., local authorities and law enforcements. Changes in expectations in turn call for changes in stimulus, knowledge, praxis, polices, laws and institutions.

*Second,* checks and balances mechanisms must effectively constrain the materialization of undue expectations. A minor observation is that, unlike the beliefs of some constitutional scholars, not only emergency powers but local authorities are not good at protecting human rights, at least in some cases where for local authorities, expectations and evaluation from citizens are less important than those from central government.

## Data Availability

No data are associated with this article.

## Footnotes

^1^ Tom Ginsburg and Mila Versteeg, ‘The Bound Executive: Emergency Powers during the Pandemic’ (2021) 19 International Journal of Constitutional Law 1498, 1534 < https://academic.oup.com/icon/article/19/5/1498/6308959?login=true> accessed 29 December 2022.

^2^ Hai Thanh Doan, ‘The Public Health Response to COVID-19 in Vietnam: Decentralization and Human Rights’ [2022] Asian Bioethics Review < https://doi.org/10.1007/s41649-022-00226-1>; Ginsburg and Versteeg (n 4) 1534.

^3^ Doan (n 5).

^4^ ibid.

^5^ Oren Gross, *Law in Times of Crisis: Emergency Powers in Theory and Practice* (Fionnuala Ní Aoláin ed, Cambridge University Press 2006) 17.

^6^ Doan (n 5).

^7^ ibid.

^8^ Directive No 16/CT-TTg dated 31 March 2020 which made lockdown possible by prescribing that *“... more than two people should not gather outside offices, schools…”*

^9^ Doan (n 5).

^10^ ibid.

^11^ Ginsburg and Versteeg (n 4) 1534.

^12^ Cong Giao Vu and Tri Uc Dao, ‘State of Emergency in Vietnamese Law: Reflections on the Government Response to the Covid-19 Pandemic’ (2022) 22 Australian Journal of Asian Law 5, 5–7.

^13^ ibid.

^14^ Bùi Thu Hằng, ‘Ban Hành Văn Bản Trong Tình Trạng Khẩn Cấp Nhìn Từ Dịch Bệnh Covid- 19 [Issuing Documents in a State of Emergency Observed from the Covid-19 Pandemic]’ (2020) 408 Nghiên cứu Lập pháp [Legislative Studies] < http://lapphap.vn/Pages/TinTuc/210491/Ban-hanh-van-ban-trong-tinh-trang-khan-cap-nhin-tu-dich-benh-Covid--19.html>; Cao Vũ Minh, ‘Pháp Luật về Tình Trạng Khẩn Cấp Của Liên Bang Nga và Những Gợi Mở Cho Việt Nam [Law on State of Emergency of the Russian Federation and Suggestions for Vietnam]’ (2021) 434 Nghiên cứu Lập pháp [Legislative Studies] < http://lapphap.vn/Pages/TinTuc/210829/Phap-luat-ve-tinh-trang-khan-cap-cua-Lien-bang-Nga-va-nhung-goi-mo-cho-Viet-Nam.html>.

^15^ Richard L Oliver, ‘Expectancy Theory Predictions of Salesmen’s Performance’ (2018) 11 Journal of Marketing Research 243, 244–245 < https://journals.sagepub.com/doi/10.1177/002224377401100302> accessed 10 January 2023.

^16^ Judee K Burgoon and Jerold L Hale, ‘Nonverbal Expectancy Violations: Model Elaboration and Application to Immediacy Behaviors’ (1988) 55 Communication Monographs 58.

^17^ Joseph Berger, Bernard P Cohen and Morris Zelditch, ‘Status Characteristics and Social Interaction’ (1972) 37 American Sociological Review 241 < http://www.jstor.org/stable/2093465>.

^18^ ibid.

^19^ Cecilia L Ridgeway, ‘Why Status Matters for Inequality’ (2014) 79 American Sociological Review 1 < https://journals.sagepub.com/doi/10.1177/0003122413515997> accessed 14 January 2023.

^20^ Mads Borup and others, ‘The Sociology of Expectations in Science and Technology’ (2006) 18 Technology Analysis & Strategic Management 285 < https://www.tandfonline.com/doi/abs/10.1080/09537320600777002> accessed 14 January 2023.

^21^ Mankiw, *Principle of Economics* (9th edn, Cengage 2021) 4.

^22^ John Maynard Keynes, ‘The Principle of Effective Demand’ [2018] The General Theory of Employment, Interest, and Money 21, 22.

^23^ John Maynard Keynes, *The General Theory of Employment, Interest, and Money* (Palgrave Macmillan 2018) 134.

^24^ ibid xxxviii, 141.

^25^ “animal spirits … a spontaneous urge to action rather than inaction, and not as the outcome of a weighted average of quantitative benefits multiplied by quantitative probabilities” (ibid, 141).

^26^ ‘Expect’ ( *Merriam-Webster*) < https://www.merriam-webster.com/dictionary/expect> accessed 11 January 2023.

^27^ Some definitions are not relevant to the paper, e.g., to be pregnant.

^28^ ‘Expectation’ ( *Cambridge Dictionary*) < https://dictionary.cambridge.org/dictionary/english/expectation> accessed 11 January 2023; ‘Expectation’ ( *Oxford Learners Dictionaries*) < https://www.oxfordlearnersdictionaries.com/definition/english/expectation> accessed 11 January 2023; ‘Expect’ ( *Oxford Learners Dictionaries*) < https://www.oxfordlearnersdictionaries.com/definition/english/expect> accessed 11 January 2023; ‘Expect’ (n 29); ‘Expect’ ( *Cambridge Dictionary*) < https://dictionary.cambridge.org/dictionary/english/expect> accessed 11 January 2023.

^29^ ‘Expectation’ (n 31); ‘Expect’ (n 31).

^30^ ‘Expect’ (n 29).

^31^ ibid; ‘Expect’ (n 31).

^32^ ‘Expect’ (n 29).

^33^ ibid.

^34^ ‘Expect’ (n 31).

^35^ ibid.

^36^ ibid.

^37^ ibid.

^38^ ibid.

^39^ ibid.

^40^ ibid.

^41^ ibid.

^42^ ‘Expect’ (n 29).

^43^ For (1), the fact that one or more people ‘expect changes…’ do not mean there is any rule or obligation dictate as such. For (2), one can daydream and even get fancy results without working hard due to, inter alia, luck, working smartly, making use of the surrounding context and changes. For (3), in the context provided by the sentence, there is a match between the expectations of guests and the real facility of the hotel but in other instances there can be mismatches which neither indicate moral nor legal wrong.

^44^ I will engage more with “counter-expectations” in the following sections.

^45^ Stanley Milgram, ‘Behavioral Study of Obedience’ (1963) 67 Journal of Abnormal and Social Psychology 371 </record/1964-03472-001> accessed 9 January 2023; Stanley Milgram, *Obedience to Authority: An Experimental View* (Harper and Row 1974).

^46^ James Waller, *Becoming Evil: How Ordinary People Commit Genocide and Mass Murder* (Second edition, Oxford University Press 2007) 111.

^47^ *See more* S Alexander Haslam, Stephen D Reicher and Megan E Birney, ‘Nothing by Mere Authority: Evidence That in an Experimental Analogue of the Milgram Paradigm Participants Are Motivated Not by Orders but by Appeals to Science’ (2014) 70 Journal of Social Issues 473 < https://onlinelibrary.wiley.com/doi/full/10.1111/josi.12072> accessed 9 March 2023.

^48^ Nick Haslam, Steve Loughnan and Gina Perry, ‘Meta-Milgram: An Empirical Synthesis of the Obedience Experiments’ (2014) 9 PLOS ONE < https://journals.plos.org/plosone/article?id=10.1371/journal.pone.0093927> accessed 9 March 2023.

^49^ ibid.

^50^ Stanley Milgram, Leonard Bickman and Lawrence Berkowitz, ‘Note on the Drawing Power of Crowds of Different Size’ (1969) 13 Journal of Personality and Social Psychology 79 </doiLanding?doi= https://doi.org/10.1037/h0028070> accessed 9 January 2023.

^51^ In 1955, following the announcement of the success of the polio vaccine trial, more than hundreds of thousands of children in the United States received a polio vaccine manufactured by Cutter Laboratories. Cutter’s vaccine contained live, infectious virus instead of inactivated virus. This caused 40000 cases of polio, leaving 200 children with varying degrees of paralysis and killing (Michael Fitzpatrick, ‘The Cutter Incident: How America’s First Polio Vaccine Led to a Growing Vaccine Crisis’ (2006) 99 Journal of the Royal Society of Medicine 156 </pmc/articles/PMC1383764/> accessed 17 February 2023).

^52^ Martin T Orne, ‘Demand Characteristics and the Concept of Quasi-Controls’ in Robert Rosenthal, Ralph L. Rosnow and Alan E. Kazdin (eds), *Artifacts in behavioral research: Robert Rosenthal and Ralph L. Rosnow’s classic books*, vol 110 (1st edn, Oxford University Press New York, NY 2009).

^53^ Elton Mayo, *The Human Problems of an Industrial Civilization* (Compass books ed, Viking Press 1966).

^54^ ibid.

^55^ Hằng (n 17); Minh (n 17).

^56^ Under Article 3.1 of LPCDD class A consists of extremely dangerous infectious diseases that can transmit very rapidly and spread widely with high mortality rates or with unknown agents

^57^ Doan (n 5).

^58^ Gustav Radbruch, ‘Five Minutes of Legal Philosophy (1945)’ (2006) 26 Oxford Journal of Legal Studies 13, 13.

^59^ Gustav Radbruch, ‘Statutory Lawlessness and Supra-Statutory Law (1946)’ (2006) 26 Oxford Journal of Legal Studies 1, 7.

^60^ ibid.

^61^ From an international human rights law perspective, as a State Party to the ICCPR, to satisfy its international commitment and avail itself of the right of derogation, Vietnam must inform the other States Parties through the intermediary of the Secretary-General of the United Nations the list of the provisions from which it has derogated or wish to derogate, accompanied by reasons (Article 4.3 of ICCPR). Hence, strictly speaking, in this dimension, even if the state of emergency was declared, in theory, it would not be a sufficient basis for derogation of Vietnam.

^62^ Doan (n 5).

^63^ ibid.

^64^ Because Vietnam followed a Soviet Commanding Economy Model, the number of legal documents extremely plentiful: annually the Government decided which goods to product, which governmental agencies would involve in the production, what would be the price of input and output goods, and even who would be distributed/sold goods and how much the volume of the distribution/sell would be.

^65^ Shiro Muto, ‘Reforming the Secured Transactions Regime in Vietnam Using Foreign Law as a Model: A Critical Appraisal’ (2002) 7 Uniform Law Review 1031, 1031, 1034; John Stanley Gillespie, *Transplanting Commercial Law Reform: Developing a ‘Rule of Law’ in Vietnam* (Ashgate Publishing, Ltd 2016) 236–239.

^66^ Vu and Dao (n 15) 7, 8, 10.

^67^ Robert O Paxton, *The Anatomy of Fascism* (1st ed., Alfred A Knopf 2004) 107, 128.

^68^ Colleen M Flood and others, ‘Reconciling Civil Liberties and Public Health in the Response to COVID-19’ (2020) 5 FACETS 887 < https://www.facetsjournal.com/doi/10.1139/facets-2020-0070> accessed 2 January 2023.

^69^ Sebastian Hellmeier and others, ‘State of the World 2020: Autocratization Turns Viral’ (2021) 28 Democratization 1053 < https://www.tandfonline.com/doi/abs/10.1080/13510347.2021.1922390> accessed 2 January 2023; Amanda B Edgell and others, ‘Pandemic Backsliding: Violations of Democratic Standards during Covid-19’ (2021) 285 Social Science & Medicine 114244.

^70^ Ginsburg and Versteeg (n 4) 1502.

^71^ Article 51 The 1992 Constitution 1992; Truong Chinh, ‘Report to the National Assembly 12 December 1980’ (1980) 15 trans. FBIS East Asia Daily Report K15.

^72^ Vu and Dao (n 15) 11.

^73^ ibid 13.

^74^ ibid.

^75^ Ginsburg and Versteeg (n 4) 1507.

^76^ John Ferejohn and Pasquale Pasquino, ‘The Law of the Exception: A Typology of Emergency Powers’ 216–217 < http://www.constitution.org/mac/disclivy_.htm.> accessed 3 January 2023.

^77^ Section 3, particularly 3.8 Nghị quyết số 30/2021/QH15 [Resolution no. 30/2021/QH15] 2021.

^78^ “The Government decides and organizes the implementation of restrictive measures. The Government ****may**** report to the National Assembly’s Standing Committee … for its consideration … *in matters relating to licensing and registering for marketing authorization and circulation, production and procurement of drugs, medical equipment, chemicals and making investment in facilities; procurement of such, and prevention of misconduct, corruption, and waste only if measures decided by the Government in ad hoc basis is insufficient”* (ibid.) The phrasing of *Resolution no. 30/2021/QH15* can be interpreted as that the Government ****may**** report to the National Assemble or National Assembly’s Standing Committee should there be needs related to institutionalized solutions.

^79^ Doan (n 5).

^80^ “As shown above, this is not a function of a resolution of either the NA or the NASC as per the 2015 Law on Promulgation of Legal Documents. The only legal document that can modify the legal effect of a law is another law. If the NA’s resolution cannot alter the effect of a law, it would make the delegation of the NA to the NASC somewhat ultra vires.” (Duc Tien Nguyen, ‘“Authorized to Depart from the Law”: The Curious Case of Viet Nam in Times of COVID-19’ (2021) XX Statute Law Review 1, 7–8 < https://www.ncbi.nlm.nih.gov/pmc/articles/PMC8499998/>.)

^81^ Carl Schmitt, *Political Theology: Four Chapters on the Concept of Sovereignty* (George Schwab and Tracy B Strong eds, University, University of Chicago Press 2005).

^82^ Vu and Dao (n 15) 12; Trần Ngọc Đường, ‘“Hiến Pháp Năm 2013 và Cơ Chế Kiểm Soát Quyền Lực Nhà Nước, Cơ Chế Bảo vệ Hiến Pháp’’’ [The 2013 Constitution and the Mechanism to Control State Powers and to Protect the Constitution]”’ in Cao Đức Thái (ed), *Cơ chế bảo hiến và quyền con người: Kinh nghiệm Cộng hòa Liên bang Đức và Việt Nam [Mechanism to protect the constitution and human rights: Experiences of Germany and Vietnam]* (Lý luận Chính trị 2015).

^83^ Pip Nicholson, ‘Renovating Courts: The Role of Courts in Contemporary Vietnam’, *Asian Courts in Context* (Cambridge University Press 2014) < https://www.cambridge.org/core/product/identifier/9781107588813%23c06608-15-0/type/book_part> accessed 4 January 2023.

^84^ Vu and Dao (n 15) 12.

^85^ Article 159.2 of Luật ban hành văn bản quy phạm pháp luật 2015 [the 2015 Law on Promulgation of Legislative Documents] 2015.

^86^ Lê Duẩn, *Cách Mạng Xã Hội Chủ Nghĩa Tại Việt Nam, Tập I [Socialist Revolution in Vietnam. Vol I]* (NXB Chính Trị Quốc Gia Sự Thật 1980). Cf writings of Hoàng Quốc Việt (Adam Fforde, ‘The Unimplementability of Policy and the Notion of Law in Vietnamese Communist Thought’ (1986) 14 Asian Journal of Social Science 60, 67 < https://brill.com/view/journals/ajss/14/1/article-p60_11.xml> accessed 4 January 2023.

^87^ Nghị quyết 51/2001/NQ-QH10 bổ sung điều của Hiến pháp nước cộng hoà xã hội chủ nghĩa Việt Nam năm 1992 [Resolution 51/2001/NQ-QH10 supplementing articles of the 1992 Constitution of the Socialist Republic of Vietnam] 2001.

^88^ Hồ Chí Minh, ‘Dân Vận [Mass Mobilization]’ (1949) 120 Sự Thật [Truth] < https://cpv.ctu.edu.vn/tin-tc-s-kin/hc-tp-va-lam-theo-tm-gng-o-c-h-chi-minh/181-mt-s-tac-phm-dan-vn-ca-ch-tch-h-chi-minh?fbclid=IwAR1Cb_S22I93Qrk3VmS2nFPUIApavyC8AxSzTwUJtK5AGfSld3J91hU_74k>.

^89^ For example, “Ghen Cô Vy” (“Jealous [of] Coronavirus”) and its associated dance, videos guiding handwashing and wearing mask have been globally popular.

^90^ Lee Ross, David Greene and Pamela House, ‘The “False Consensus Effect”: An Egocentric Bias in Social Perception and Attribution Processes’ (1977) 13 Journal of Experimental Social Psychology 279.

^91^ A controversial program of VTV - the national television broadcaster of Vietnam, even explained that the human brain had three parts “reptile brain,” “animal brain”, and “human brain”, “in the reptilian brain, fear will turn into instinctive actions, such as trying to escape authorities. In the animal brain, fear dominates human behaviour in the form of emotions, such as: “it’s boring at home, let’s go out….” People believed that the program had used the “triune brain theory” as a metaphor to humiliate people. Bạch Dương, ‘Ví Não Người vi Phạm Với “Não Bò Sát”, “Não Thú”, VTV24 Gây Tranh Cãi [Likening Rule-Breachers’ Brain to “Reptilian Brain”, “Animal Brain”, VTV24 Caused Controversies]’ ( *Báo Giao thông [Traffic Newspaper]*, 31 July 2021) < https://www.baogiaothong.vn/vtv-co-vi-pham-luat-khi-vi-nao-nguoi-voi-nao-bo-sat-nao-thu-d518631.html> accessed 30 December 2022.

^92^ Solomon E Asch, ‘Studies of Independence and Conformity: I. A Minority of One against a Unanimous Majority.’ (1956) 70 Psychological monographs: general and applied. 1.

^93^ Philip G Zimbardo, *The Lucifer Effect: Understanding How Good People Turn Evil* (1st ed., Random House 2007).

^94^ Paul Louis Félix Philastre, *Le Code Annamite: Tome Premier* (Leroux 1876); Gustave Dumoutier, *Essais sur les Tonkinois* (Imprimerie d’Extrême-Orient 1908).

^95^ Tu Phuong Nguyen, ‘Flexible and Compassionate, or Violent and Intimidating?: Various Accounts of Law in Vietnam’ (2022) 54 Critical Asian Studies 67.

^96^ ibid; Vu Anh Dao, ‘Tiền Là Tiên Là Phật [Money Is as Powerful as God and Buddha] - Investitgating the Persistent of Corruption in Vietnam’ (Victoria University of Wellington 2017).

^97^ Steven A Mccornack and Malcolm R Parks, ‘Deception Detection and Relationship Development: The Other Side of Trust’ (1986) 9 Annals of the International Communication Association 377 < https://www.tandfonline.com/doi/abs/10.1080/23808985.1986.11678616> accessed 20 January 2023.

^98^ Web Portal of Police of Binh Thuan Province, ‘Chiêu Bài “Dân Chủ, Nhân Quyền” - Mũi Tấn Công Thâm Độc Của Các Thế Lực Thù Địch [The Guise of “Democracy, Human Rights” - The Malicious Attack of Hostile Forces]’ (29 April 2022) < https://congan.binhthuan.gov.vn/chieu-bai-dan-chu-nhan-quyen--mui-tan-cong-tham-doc-cua-cac-the-luc-thu-dich-N1115.aspx> accessed 8 January 2023.

^99^ Matheus Pelinski da Silveira and others, ‘Physical Exercise as a Tool to Help the Immune System against COVID-19: An Integrative Review of the Current Literature’ (2021) 21 Clinical and Experimental Medicine 15; Kathryn L Humphreys, Myo Thwin Myint and Charles H Zeanah, ‘Increased Risk for Family Violence during the COVID-19 Pandemic’ (2020) 146 Pediatrics.
