# Conceptualizing expectations - Vietnam’s responses during COVID-19 as an illustration

**DOI:** 10.12688/wellcomeopenres.18972.2

**Published:** 2024-01-24

**Authors:** Hai Doan

**Affiliations:** 1 Bioethics Centre, University of Otago, Dunedin North,, Dunedin, 9016, New Zealand

**Keywords:** expectations; expect; human rights; COVID-19 pandemic; emergency powers; Vietnam; the state of exception.

## Abstract

**Background:**

A key part of the complexity of actual social life lies in the fact that it operates on a large web of not only prescribed norms and rules, but also explicit and especially implicit expectations which constantly interact with each other.

**Methods:**

This paper offers the first in-depth study on ‘expectations’ in bioethical and health law studies, taking Vietnam’s responses to coronavirus disease (COVID-19) as an illustration.

**Results:**

It defines ‘expectations’ as normative imaginaries that can induce or guide actions and inactions at individual and collective levels.

**Conclusions:**

It suggests that ‘expectations’ is a fruitful concept for bioethical and health law studies because it helps better understand the complexity of interactions in society and patterns of thinking and acting of actors in real-world contexts. Studying expectations has implications for governance and future research agendas.

## Plain Language Summary

This paper offers the first in-depth study on ‘expectations’ in bioethical and health law studies, taking Vietnam’s responses to coronavirus disease (COVID-19) as an illustration. It defines ‘expectations’ as normative imaginaries that can induce or guide actions and inactions at individual and collective levels. It suggests that ‘expectations’ is a fruitful concept for bioethical and health law studies because it helps better understand the complexity of interactions in society and patterns of thinking and acting of actors in real-world contexts. Studying expectations has implications for governance and future research agendas.

## 1. Introduction

Some scholars expect that local actors can help to check the power of the central government ^1^. There is a general expectation that local actors are capable of calibrating policies, responding to local needs, conditions, and preferences, as well as reducing abuses to a lesser extent ^2^. Within the Vietnamese context, that kind of expectation justified the delegation of power – the process in which higher authorities delegate power to lower ones to make and implement public health measures – during the COVID-19 pandemic ^3^. Yet, the delegation of power fermented conditions for unbounded powers of local officials, thus leading to abuses of powers and possible violations of rights ^4^. But why were there human rights violations if such violations are legally unjustified and manifestly ethically horrendous, especially considering that some behaviours even seem strange and unreasonable and local actors have been presumed to calibrate policies to respond to local needs? Amidst that context, some Vietnamese scholars expected the declaration of the state of emergency, but why did scholars expect so? That is to ask, how could the declaration of the state of emergency make a difference and/or how could the expectation be understood as making sense?

It appears that underneath many propositions or actions, there might have been certain expectations. Real-world society is complex and perhaps a key part of the complexity of actual social life lies in the fact that it operates on a large web of not only prescribed norms and rules but also explicit and especially implicit expectations which constantly interact with each other. ‘Expectations’, thus, is a significant concept to address the complexity in the patterns of thinking and acting of subjects, institutions, and society. The concept of expectations signifies the interactions of selves inter se. Unfortunately, this concept is under-conceptualized in bioethical and legal studies. This paper conceptualizes ‘expectations’ and indicates some implications of this concept. The conceptualization is done by the analytical method of interpreting existing definitions, gathering relevant accounts across disciplines and using cases, particularly responses to the COVID-19 pandemic within the context of Vietnam together with other cases. The rationale for Vietnam being chosen as an illustration boils down to not exclusively Vietnam being an under-studied jurisdiction but also an interesting case study. Historically, the mode of governance in North Vietnam was a mixture of family-based, village-based and regional-based ^5^. In South Vietnam, the governance structure was quite similar but looser and more liberal for the reason that people there were originally from different regions and of different origins and came there as settlers ^6^. In brief, Vietnam had a governance structure featured by ‘local units’. Central dynasty courts attempted to centralize powers but were not quite successful in general ^7^. Culturally, being influenced by Confucianism, social norms (ethics, rituals) played a crucial role and had been more emphasized than the law (Analects 2.3). Notwithstanding, Confucianism is not the only school of thought in Vietnam: traces and impacts of Buddhism and many other different schools of thought on society and behaviours have been profound ^8^. Thus, social interactions in Vietnam are rich ^9^. In the modern time, Vietnam is a one-party-ruled state. Its governance structure was modelled after the Soviet Union. That signifies not only the bureaucratic nature but also a centrally-focused governance structure. Being modelled after the Soviet Union also signifies the deemphasizing of human and natural rights ^10^. With the collapse of the Soviet Bloc and economic hardship, Vietnam opened to liberal order and has been in transition, signifying a welcome to the Rule of Law, human rights and decentralization - the transferring of more powers to local actors ^11^. Formal institutions like law are more and more emphasized. Thus, various transitions and struggles between factors can be observed. This paper defines ‘expectations’ as normative imaginaries that can induce or guide actions and inactions. It suggests that ‘expectations’ is a fruitful concept because it helps better understand the complexity of interactions in society and patterns of thinking and acting of actors in real-world contexts. This has implications for governance as well as research. The second section offers some cases to show that a range of expectations can be observed in times of crisis. The third section conceptualizes ‘expectations’. The conceptualization is done by the analytical method by which we interpret plain meanings of expectations provided in/by dictionaries and induce them into the concept of expectations. Then, the concept is demonstrated as embedded in the understandings of expectations of various disciplines. That validates and reinforces the conceptualization of ‘expectations’ of this paper. Thus, that allows an interpretation that understandings of various disciplines concerning expectations can be deduced from our conceptualization, and, vice versa, the conceptualization can still be achieved by inducing from these understandings. That allows the process of conceptualization to be hermeneutic. Understandings of expectations of various disciplines offer indicators concerning the features and operation (both the mode and implications of operation) of expectations. That allows the paper to theorize these matters. The fourth section explores some implications of the concept of expectations for governance and research. For clarity, this paper deploys phrases ethics and law and/or rights together to signify that they are not synonymous but should be simultaneously considered in relation to a particular issue.

## 2. Cases

### 2.1. Local powers vs central powers

In the paper titled *‘The bound executive: Emergency powers during the pandemic’*, studying different jurisdictions, Tom Ginsburg and Mila Versteeg suggest various ways according to which ‘executive power’ can be claimed to have been bounded during COVID-19 pandemic. Their imagination of ‘executive power’ is, however, only ‘central executive over-reach’ ^12^. Perhaps, upon this account, they phrase local actors as ‘subnational constraints’ ^13^. ‘Subnational constraints’ and their actions, including ‘resistance to central government directives’, ‘more restrictive approach than that of the central government’, ‘ignorance of central government’s directives’ seem to be endorsed. The term ‘subnational constraints’ and the overall tone of writing ^14^ suggest that they saw such actions as positive. But why is ‘bounded executive power’ constrained upon the central government’s power but not local governments, and why is disobedience or negligence (even unlawful) of local governments against policies of the central government executive constraints? Aren’t the powers of local government also executive? Or cannot local government’s executive powers pose threats to citizens? Thus, can it be expected that local authorities are ‘subnational constraints’?

### 2.2. Possible human rights violations by local authorities in Vietnam during the COVID-19 Pandemic

There can be no more salient case study that gives rise to the above questions than the Vietnamese situation. With - and even without - formal delegation of powers, local governments established checkpoints in their ‘own territories’ and invented significantly divergent rules for their own ‘fortresses’. For example, in some localities, entry of outsiders (people who do not reside there) was banned; in some other places, people were required to test at checkpoints (all other test results, even those provided by previous checkpoints within the previous few seconds, would not be accepted). Within that context, local governments might be possible sources of human rights violations ^15^. It is reported that local officials have locked citizens' houses with citizens inside, worrying that without such ‘lock-up’, citizens might go out and spread the disease. Tellingly, it is reported in a case (the “ *Bread is not Food”* case), officials first ‘taught’ a citizen that Directive 16 ^16^ only allowed citizens to go out for essential purposes, while buying food was not; such officials then alternatively argued that “bread is not food”. ^17^ In another instance, citizens were coerced by force to take a COVID-19 test administered by public officers. ^18^ But why did officials – *in plural* - commit human rights violations if such violations were manifestly unlawful, ethically horrendous, and unreasonable (in their own initiatives)?

### 2.3. Declaration of emergency

Amidst that context, some Vietnamese scholars believe that the central government should formally declare the state of emergency. This can be observed, for example, in the statement of Vũ Công Giao and Đào Trí Úc – two prominent scholars in the area of constitutional law and socialist rule of law-based state ^19^:

*“As to the misuse of emergency powers, Directive 16* ^20^ *actually caused human rights derogation without declaration of a state of emergency as required by the 2013 Constitution”* ^21^.

By mentioning ‘misuse of emergency powers’, describing many potential violations of human rights, and resorting to the discourse of human rights, they expressed their concerns for emergency powers and human rights. However, the argument that a formal declaration of emergency is a necessary condition for emergency powers and human rights derogation is unconvincing because, different to the International Covenant on Civil and Political Rights (ICCPR), the 2013 Constitution does not make a distinction between restricting rights and derogation from rights. Moreover, under Article 14 of the 2013 Constitution, all rights (including non-derogable rights as provided for under the ICCPR) can be restricted. Satisfying Article 4.3 of ICCPR is, however, a different matter because the procedure thereof relates to notification of/from a state to other states ^22^. Nor can it be argued that only in light of a formal declaration of emergency would the Vietnamese Government have sufficient powers ^23^. The reason for this is that on April 1, 2020, Prime Minister Nguyễn Xuân Phúc signed Decision 447/QD-TTg recognizing the COVID-19 epidemic as ‘ *class A infectious diseases’* ^24^. According to the Law on Prevention and Control of Contagious Diseases (LPCCD), measures can be found in Article 54 (measures to be applied in a state of emergency in case of epidemic) can similarly be found in a range of Articles, from Articles 46 to 55. Policy documents, e.g., Decision 447/QD-TTg, recalled LPCCD in tracing their validity and mentioning measures.

Hardly can a declaration of emergency be linked to better supervision of the National Assembly and Judiciary. On the one hand, it is because of the “ *consensus in the Vietnamese political system, led by the Communist Party”* ^25^ and the lack of independence of the mentioned institutions ^26^. On the other hand, authorization and supervision do not necessarily take the form of a formal declaration of emergency made in advance of the deployment of measures. ^27^ Moreover, the Vietnamese National Assembly did authorize the Vietnamese Government much later at the climax of the Delta wave under Resolution No. 30/2021/QH15 dated 28 July 2021, which is a blanket authorization ^28^.

It can be argued that the declaration of emergency allowed the emergency powers to be expected to be temporary. However, while it can be argued that the declaration of emergency is not a prerequisite condition for emergency powers to be temporary, that is, without any declaration, measures can still be temporary, it can also be argued that with the declaration, measures are not only legitimatized but also perpetuated ^29^.

Notwithstanding, it can still be observed that there are certain expectations for and drawn from the law. 

## 3. Expectations

It can be observed that there are expectations in all the above cases. Perhaps the reason for which expectations appear in all cases is simply that except for someone who loses the capacity of cognition and perception, expectations as belonging to or presenting cognitive ability must exist. As this paper will demonstrate in subsequent sections, expectations not only represent human innate tendencies but also embed social experiences.

### 3.1. Definition of expectations

‘Expect’ or ‘expectation’ are words used popularly in daily life. Generally, dictionaries for English users define ‘expectation’ or ‘expect’ ^30^ with the following meanings ^31^:

1) A belief, or to think or believe, something will happen ^32^
2) A belief or to think that someone should behave in a particular way or do a particular thing ^33^ or to consider reasonable, due, or necessary ^34^ or to consider bound in duty, responsibility or obligated ^35^.
3) To suppose/a supposition ^36^
4) To await/ awaiting ^37^.

Meaning (2) seems to suggest that expect/expectation may appear as a counter-partner of duty or obligation, but in scrutinizing examples offered by dictionaries, it is not always the case. Examples provided by dictionaries are as follows:

It is reasonable to expect changes in the way we work ^38^. (1)
No one has a right to expect good results without working hard ^39^. (2)
These are the high standards that hotel guests have come to expect ^40^. (3)
He's still getting over his illness, so it's unrealistic to expect too much from him ^41^ (4).
Are you clear what is expected of you? ^42^ (5)
You can't reasonably expect people to pay such high taxes ^43^ (6).
We are expected to work on Saturdays ^44^ (7).
I expect to be paid promptly for the work ^45^ (8).
They expect you to pay your bills ^46^ (9)

From (1) – (3), the sense of legal or moral obligation or responsibility or duty is vague ^47^.

For (4) and (5), subjective expectations from one side need not express a clear sense of being bound by duty, being obliged or obligated, being mandated, or incurring responsibility from the other side. The other side may not be aware of or may dismiss such expectations.

For (6) and (7), there are contradictions in the sense of being bound. In (6) there can be an obligation under the law which can be irrational or morally invalid. It is the opposite in (7), a feeling of being morally obliged to work extra time can be contrary to the law.

For (8) and (9), the actions at stake can be negotiated, subject to various contextual factors without implying moral or legal wrongs. Consequently, these examples might suggest that certain actions are desired or looked for (irrespective of who carries out such actions).

It would follow from what has been demonstrated that ‘expect’ and ‘expectations’ can also be understood as *“to look for”* or *“to desire”* or *“a desire or an ambition*” and *“to hope”* or *“a hope”*. As a result, ‘expects’ or ‘expectations” do not refer to mere ‘acts of believing’ or ‘beliefs’ in a neutral sense, but ‘to believe’ with or ‘beliefs’ having normative propositions or judgements. Underlying the normative beliefs are certain imaginaries. 

It would follow from the above that ‘expectations’ can be defined as *normative imaginaries that can induce or guide actions and inactions (generally ‘actions’).*

‘Expectations’ has long been an important concept for many intellectual fields and theories, which aim to offer explanations for a range of social phenomena and issues. Different understandings of disciplines concerning expectations link to each other to a certain extent and can supplement each other. It can also be observed from the investigation of these understandings that the concept of ‘expectations’ as conceptualized above is embedded in these understandings. That is, though various learnt fields and theories refer to ‘expectations’ conventionally in light of one or other meanings for the purpose of such theories or fields, at the essence of these understandings is what this paper conceptualized above. That validates and reinforces the above conceptualization of ‘expectations’. Thus, that allows an interpretation that understandings of disciplines concerning expectations can be deduced from our conceptualization and vice versa, the conceptualization can still be achieved by inducing from these understandings.

In the ‘expectancy theory of motivation’ in organizational behaviour, expectations - the *subjective probability* of the attainment of rewards - are *incentives* that motivate employees’ job performance ^48^. That is, people respond (or are motivated), to a certain degree, to their guess/guesstimate of rewards they can receive. Yet, it is also expected that people will gradually cease to respond to such rewards at some point or under some conditions where they expect otherwise. In short, *expectations are beliefs and practical reasons*.

‘Expectations’ is a core concept in economics and shows its intriguing significance therein. Classical economics studies claim that expectations are among the factors that drive decisions (i.e., *practical reasons as mentioned above*). For example, expectations about the future may affect both demand and supply for a good or service today ^49^. But that does not capture the full picture. Let’s imagine a case in which the price of fuel is expected to increase tomorrow. Per classical economics’ accounts, people should buy fuel today. But if many people behave rationally, running to buy and even hoard fuel, consequently, due to high demand, they must wait in a long queue and some people may have to leave without having fuel (due to shortages). Keynes, thus, recognized that a practical decision would not entertain a single undoubting expectation, but *several hypothetical expectations,* held with *varying degrees of probability and definiteness* ^50^. According to Keynes, our own preferences are not as important as the anticipation of *“what average opinion expects average opinion to be”* ^51^, i.e., expectations of an individual about the market are expectations of the average expectation of the market which is the average of the sum of individuals’ expectations. Keynes also recognized that though expectations would be shaped partly by knowledge and creativity, they could be unstable ^52^ and sometimes irrational, influenced by *‘animal spirits’* ^53^. Keynes, thus, suggested that *expectations are not only various, interactive and adaptive but can also be unstable, irrational, and wishful and can also even be a determinant of realities*.

Perhaps not by coincidence, there is a kind of imagination of *‘animal spirits’* and ‘ *a determinant of realities’* in science and technological sociology studies. Expectations as *wishful enactments of a desired future* are claimed to underline bold technological advancements ^54^.

These accounts have certain commonalities with the Thomas theorem: “If men define situations as real, they are real in their consequences” ^55^ and the self-fulfilling prophecy in socio-psychological studies “a belief or expectation, correct or incorrect, could bring about a desired or expected outcome” ^56^; “men respond not only to the objective features of a situation, but also, and at times primarily, to the meaning this situation has for them. And once they have assigned some meaning to the situation, their consequent behaviour and some of the consequences of that behavior are determined by the ascribed meaning” ^57^.

In sociology, according to the ‘expectancy violations theory’, expectations refer to *social norms* and *judgements concerning common patterns of behaviours in a given society that actors are expected to follow* ^58^ *,* which impact the attitude of individuals vis-à-vis actions of other individuals and those individuals. According to the ‘Expectation states theory’ ^59^, expectations refer to some kinds of *practical beliefs*, i.e., beliefs about practical issues, and even *stereotypes,* which form status hierarchies ^60^ and social inequality ^61^. It follows that these studies suggest that *expectations can be both norms and mechanisms to implement norms (coercion and sanction, formal and informal*, including temptations to meet expectations). Meanwhile, *the realities that expectations can facilitate include the systems of norms.*

In law, expectations have been acknowledged as relating to three aspects: the facilitation, formulation, and legitimation of legal norms and governance, the frustration of legal norms and governance, and the protection of legitimate expectations:

*The facilitation, formulation, and legitimation of legal norms and governance:* Mark L. Flear asserted that the concept of ‘expectations’ has “obvious contemporary importance” for biomedical governance and regulation and legal and regulatory studies ^62^. He considers “expectations … can be shaped by organizations and used by them as techniques for public legitimation of their governance and regulatory activities” ^63^. He considered that the weighing of positive expectations (benefits of new science and technology) and negative expectations (concerns and risks) together with reasons for regulation provide a basis for legitimizing new science and technology and for informed consent to treatment and human participation in health research. ^64^

*The frustration of legal norms and governance:* It has been found that expectations formed within the social context and influenced by social norms can distort and jeopardize legal initiatives and implementations of legal provisions. For example, it has been argued that the initiative to introduce jury trials in Japan in criminal trials would be destined to fail in light of expectations of lay citizens towards experienced judges and expectations to not argue with one another and to question authority ^65^.

*The protection of legitimate expectations:* ‘Legitimate expectations’ is a concept/doctrine of importance in law and legal studies. ‘Legitimate expectations’ are grounded not only on promise ^66^ but ‘good administration of justice’ ^67^. Legitimate expectations are inherently linked to justice and fairness, reasonableness, and ethics ^68^. Expectations are legally protected only if they are reasonable in light of the circumstances ^69^ and must not be undue or illegal ^70^. Thus, legitimate expectations are formed not only upon representations but also upon practices and the legal system and legal provisions and links with legality and morality ^71^.

These theories suggest features and operation (both the mode and implications of operation) of expectations, which shall be explored in subsequent subsections.

### 3.2. Objects of expectations

Drawing from the above accounts, several objects of expectations can be figured out:

- Expectations of a self upon that self.
- Expectations of a self upon other selves. The term ‘selves’ is used to capture both individuals and entities that are collections of individuals, as these entities have their own lives, capabilities, and expectations distinguished from those of their constituents. This can be observed with corporations or states.
- Expectations of selves upon a self.
- Expectations of selves upon other selves,
- Expectations of a self or selves as to an event or outcome or manifestation (in general ‘event’): Such an event may be driven by selves or just by beyond-control factors, i.e., forces of nature and logic of the universe, e.g., something such as the cause-effect relationship or physical logic underlying the universe. In the cases in which an event is driven by selves, to say ‘an event’ is to stress that what is expected is that event, as opposed to the conduct or characteristics of the selves bringing about such an event. Such expectations may be instrumental (i.e., selves are means to achieve the end) but not necessarily (selves may not concern who brings about that event or how such an event could be brought about).
- Expectations of selves on, for, or are derived from institutions, formal and informal (also called selves). These institutional selves, established and operated by human selves, may be separate and independent from human selves.
- Expectations of institutional selves upon or for human selves. Those human selves creating and administering or involved in institutional selves might have expectations for institutional selves which are expressed by institutional selves. Meanwhile, in their course of operation, the totality of expectations of those creating and administering or involved in institutions makes institutional selves have separate expectations on their own. These expectations target and interact selves qua institutions but not qua human selves ^72^.

### 3.3. Some key characteristics of expectations

***3.3.1. The variety***

*First*, expectations vary among selves. All selves have their own expectations, drawing from not only selves’ personalities, past experiences, skills, knowledge, and confidence but also the purposes of these, contexts (i.e., social interactions and expressions), and components of selves.

*Second,* expectations of selves may change from time to time, sometimes just in an instant.

*Third,* expectations are various in terms of issues, contents and the associated degree of desirability. Expectations are not only about ‘trust/believe or not’. One person can be untrustworthy; however, another person may still have certain positive expectations for him/her (not to say negative expectations e.g., that the former person may not keep a promise).

*Fourth,* selves may have various expectations at the same time for themselves or others. Such expectations may even be in tension or contradictions with each other. 

*Fifth,* expectations are various in terms of certainty.

These statements shall be continuously elaborated in subsequent paragraphs and sections.

***3.3.2. The rational vagueness***

Expectations can be rationally vague. In saying so, this does not mean that expectations are neither rational nor irrational, but that they may receive a continuum of values with ‘rational’ and ‘irrational’ at polar, depending on various factors. For example, expectations for the manifestation of the Rule of Law do not seem ex facie rational within the context of an authoritarian regime, but whether they are entirely irrational is a question that cannot be addressed straightforwardly. Whether specific expectations are rational (and to what extent) depends not only upon subjective assessments of that given subject but also on “counter-expectations” and assessments of other actors. Keynes’ example shows that rational expectations are only rational as long as they can expect others’ expectations even if the latter can be irrational. The rationality of expectations is, thus, contingent upon uncontrollable factors. Expectations are also rationally vague in the sense that it is trans-factual: ‘Unrealistic’ expectations may sometimes result in unimaginable outcomes, good or bad, or help to achieve all or some unrealistic targets but sometimes are counterintuitive. Hardly can it be asserted in advance and in abstract whether (and which) expectations are unrealistic. The iron-willed ‘Zero Covid’ policy and its unshakable implementation is an example.

***3.3.3. Moral vagueness***

Expectations can be consequential (inclination to rewards, pleasure or aversion of pains or punishment or risks or disrepute or losses) or deontological or just authority-oriented or herd-oriented (i.e., unidentified moral values). Expectations of selves thus can be clearly moral or immoral, or morally uncertain, e.g., in case of dilemmas, in times of uncertainty, or due to controversies as to the definition and the requirements of morality, or simply not attending to morality at all. Stanley Milgram conducted two experiments. The first is ‘Obedience to Authority’, suggesting that (i) ordinary people may expect themselves to follow orders even when doing so makes them agents in a terrible destructive process ^73^; (ii) ordinary people may expect authorities to be truthful. There are explanatory accounts for the behaviours of ‘teachers’ as such behaviours were due to expectations that ‘learners’ would not be permanently damaged, formed upon reassurance from the experimenter ^74^. Notwithstanding, such expectations, if they existed at all, would neither be rational nor ethical: People without physics knowledge can still expect that 300–450V would be harmful and even deathly; nor are they supported by contextual factors (screams of learners); (iii) wrongdoings, immoralities, and harms can predicate upon expectations of a singular fantastic cause while overlooking related surrounding possible ethical concerns and peripheralizing other interests ^75^. The second experiment known as the glaze-following effect demonstrates that the probability of a person following the stimulus group’s action increased with the size of the stimulus group ^76^. It suggests that expectations can be formed in response to the behaviours of others.

Conversely, expectations play a role in determining decisions or actions by expecting moral values of these decisions or actions once implemented. For practical purposes, the debate on vaccine mandates serves as an example. Proponents of vaccine mandate campaigns may expect a scenario in which, in the absence of wide coverage of vaccination, millions of people may die excessively, and in the absence of a vaccine mandate, a significant portion of the population may refuse vaccination. Those expectations give grounds for expectations for moral values of vaccine mandate, especially in the absence of free consent. Opponents of mandatory vaccination expect that people are rational and shall choose what is optimal for them or that the vaccine is ineffective or even defective, recalling the ‘Cutter Incidence’ ^77^, or at least dangerous to certain people who cannot be predetermined, however.

To return to the case of Vietnam, the mentioned vagueness can be observed concerning described violations. Since the beginning of the COVID-19 pandemic, the central government constantly conveyed messages such as “Surely, we will soon defeat the COVID-19 pandemic” and “Rigid and harsh restrictions are the only way to eradicate COVID-19 and to win the battle against COVID-19”. The Vietnamese National Television (VTV), in response to criticisms to reconsider restrictive measures, claimed that *“Living with COVID-19 is the rhetoric of the rebels”*. Thus, there is a reason to believe that though officials’ justifications, invoking the protection of public health, and even the health of individuals whose rights were violated, can be a pretext, they can also represent honest but staunch expectations. This implies that violations not only can be derived from the temptation to manifest and abuse powers deeply veiled inside selves ^78^, fuelled by culture, social factors, and the state of crisis ^79^, but also may just simply follow orders or might be rooted in expectations for the good. An empirical study by Rachel Wahl reinforces this point. It suggests, within the context of India and concerning violence of the police, that there are moral and social imaginaries that inform and underlie violence. ^80^ A similar line of logic is possibly applicable within the context of Vietnam: Local authorities may have expectations derived from messages and promises of central authorities and state media. In addition, for so long, officials have been familiar with the idea that claiming rights is selfish and individuals claiming rights have ill intentions ^81^. These thus follow that violations can also be victims of mentality biases and heuristics, including, expectations of consensus from the masses ^82^ or “authority bias” (described in the “Obedience to Authority” above) or “conformity bias” ^83^, which might be aggravated under real-world conditions due to real political hierarchies, authorities, responsibilities, and punishments ^84^.

***3.3.4. Expectations are ambiguous***

Expectations are ambiguous in themselves. First, as selves may have various expectations at a given time or from time to time, sometimes they are not clear what they expect for or expect for the most. These expectations may even run counter to each other. For example, expectations underlying calls for the declaration of emergency and expectations for human rights protection might not necessarily correlate with each other.

Second, as a corollary of the first and/or due to the complexity of contextual factors, the decoded messages conveyed by the signalling expectations are vague, thus making ‘counter-expectations’ formed upon such messages vague: A self may receive various messages that they try to interpret, and the interpretation process results in various contradictory imaginaries of signalling expectations and thus leading to contradictory counter-expectations. Sometimes, this vagueness of counter-expectations is accidental or due to poor interpretation skills, but sometimes it is intentional. Perhaps, this tendency is due to, first, the human instinct that prefers decoding something complex to just reading public statements or the confidence in privileged intelligence over common knowledge. Second, sometimes, high authorities or sovereignties or rulers or bosses do not want to outspeak their position and thus express themselves vaguely and incomprehensively by different contrasting statements. A possible reason for this is that they want to maintain themselves as considerate selves. Unpublicized and hidden messages are observable phenomena and are salient in some societies. It thus follows that, thirdly, guessing what is in the mind of a boss, high-ranked official, ruler, sovereignty (“Ðoán ý”) is not a rare phenomenon in certain societies and can be observed throughout history and empirically. These imply that to the extent that two messages an actor received clash, the actor may be inclined to opt for the message that the actor is confident that shall minimize risks and harms on its side and shall make every attempt to get rid of any such risks and harms. It would follow that what local actors expect as expectations of the central government for them has much more gravity than what the central government addresses publicly.

This can be observed with the tragic “Great Leap Forward Campaign” in which local officials fearful of Anti-Rightist Campaigns left farmers to starve to death or with repeated concealment of SARS and COVID-19 outbreaks in China. Within the context of Vietnam, though the Prime Minister interpreted Directive 16 as neither a lockdown nor a traffic ban and warned against divergent, inconsistent, and excessive measures of local authorities, the Government also required that local authorities’ measures must be ‘one-level higher’ and ‘one-step earlier’ than the measures of the Government and that localities, where disease circulated, would be held accountable ^85^. As the messages are somewhat conflicting, presumably, local officials shall opt for messages that are less risky. In fact, while officials who violate rights are not dismissed, some officials have been dismissed because the disease circulated in their territories.

***3.3.5. The contextual impacts***

Not only internal traits, e.g., genes, brain, and nerves, and mental biases and shortcuts but contextual factors, i.e., social roles, social interactions, or social expressions, play a significant role in expectation-formation. As mentioned above, context aggravates and relieves certain instinct tendencies. Expectations are shaped by and/or respond to context. Tom Ginsburg and Mila Versteeg’s expectations are not surprising within the context of the US (where they reside), where there have been worries about the potential tyranny of the central despot or Leviathan ^86^, which prompted Publius (the alias standing for Alexander Hamilton, John Jay and James Madison) to publish a series of essays to defend federalism. Powers were delegated by the people to the central government and local governments in parallel, but powers were still intended to reside largely in local governments which hold general jurisdiction. Nevertheless, whether local governments can be generally expected to serve as constraints to the central government’s powers is a different matter. Within the context of Vietnam, in light of Confucianism, there have been imaginaries and expectations for benign despot ^87^. Rulers rectify wrongdoings of local mandarins, and not the other way around. Today, generally, powers are centralized in the central government, from which some are delegated to local governments. It follows that the declaration of emergency (and its rationale) can be understood on account of the combination of different contextual factors, including the theory of mingjiao (‘the doctrine of names’) and within the continued transition of Vietnam towards rule-of-law-based states and many promises concerning and expectations for the law, the Rule of law, human rights protection. ^88^

### 3.4. Interactions of expectations

Because society is a web of interactions between selves, and between selves (human and institutional), expectations do not exist in a vacuum or singularity but in a web. It is to say that expectations may direct or respond to other expectations.

***3.4.1. Signalling expectations and counter-expectations***

As expectations are a subjective state of mind, selves need not be triggered (by other expectations (‘signalling expectations’) of other selves directed at the former selves or by stimulus) to form expectations. Notwithstanding, expectations are also formed in response to signalling expectations. Expectations formed in response to signalling expectations are called ‘counter-expectations’. However, counter-expectations are not direct products of signalling expectations. Rather, counter-expectations are products of subjective interpretations or imaginaries of meanings of a complex web of factors, among which signalling expectations are a factor. As a result, expectations to respond to expectations are more accurately framed as expectations to respond to presumed signalling expectations. Such interpretations of messages upon which counter-expectations rely and form are subjective and can be false - if there are any signalling expectations at all.

***3.4.2. States of interactions***

**3.4.2.1. Meeting expectations**

Selves may try to meet expectations of other selves, especially those of higher authority. This can, for example, be observed in behaviours of Vietnamese authorities in response to implicit expectations of higher authorities, as described in the cases above. In this pattern, presumed signalling expectations are interpreted as normative and authoritative. Selves assuming the presumed signalling expectations are directed toward them may have a sense of being bound. There are two sub-categories: (i) intentional meeting expectations; (ii) de facto meeting expectations.

(i) *intentional meeting expectations:* Intentional meeting expectations refers to situations in which selves intend to meet the interpretations of presumed signalling expectations. For example, in psychology, there is a phenomenon called *demand characteristics* in which participants (of experiments), upon expectations that they will somehow be evaluated, subconsciously change their attitude and behaviour to match [interpretations of] expectations of the experimenter or to appear more socially or morally responsible ^89^.
(ii) *de facto meeting expectations:* if intentional-meeting-expectations acts neatly fit (though it is not necessarily perfectly fit) with real signalling expectations, following accurate interpretations of signalling expectations in whole or in substantial part, such acts are also de facto meet signalling expectations.

**3.4.2.2. Fulfilling expectations**

There are instances in which expectations are fulfilled but not met. The difference between ‘fulfilling’ and ‘meeting’ is that for ‘fulfilling’ the selves acting do not feel signalling expectations are positive or authoritative. 

De facto meeting expectations acts of selves may make the selves sending signalling expectations feel that their expectations are met; however, in case signalling expectations are fulfilled in light of actions which are derived from counter-expectations which are thought to be formed upon accurate interpretations of signalling expectations, but in fact, upon misinterpretations, signalling expectations and counter-expectations are reinforcing expectations. Expectations are also reinforcing in cases where signalling expectations [‘expectations (i)’] are entirely missed and actions that please such expectations (i) are derived from independently considered expectations [expectations (ii)] (e.g., in directing at different sets of expectations [‘expectations (ii) or (iii)’]. Expectations (ii) are not counter expectations of expectations (i), rather expectations (i), (ii), and possibly (iii) are reinforcing.

**3.4.2.3. Meeting or fulfilling unidentified expectations**

The famous Hawthorne effect suggests that people tend to change their behaviour should they expect that they are being observed ^90^. In these instances, selves may form certain expectations in response to unidentified expectations of unidentified other selves or social norms.

**3.4.2.4. Failing expectations**

Failing expectations may happen in two scenarios. The first is that signalling expectations are missed or misread, consequently, selves act in a manner that displeases the signalling expectations. The second is that though signalling expectations are interpreted accurately in total or in substantial part, attempts to achieve what is expected or satisfy the needs behind the signalling expectations fail. Failing expectations may result in punishments or unfavoured treatments from selves emancipating signalling expectations.

**3.4.2.5. Evading and resisting expectations**

Sometimes, selves may attempt to evade or resist expectations directed upon them should there be conflicts or clashes in expectations between the former selves and selves sending signalling expectations or between expectations of the latter with any third parties who are more mattered to the former. The “bank wiring room experiments” suggest that despite payment incentives, productivity decreased due to other expectations of workers, for example, that their productivity may have been boosted to justify firing some of the workers later ^91^.

**3.4.2.6. Beyond expectations**

Sometimes, actions are beyond expectations. There are several layers of meanings for ‘beyond expectations’. The standard understanding of the phrase provided by some dictionaries is that “act is more than people thought would be the case”, usually with positive connotations. There are other layers as well. First, cases in which consequences or outcomes of actions can be either better or worse than the initial expectations (desires) of the selves implementing actions. Second, cases in which expectations are directed on selves (directed selves) by other selves (directing selves), and it is either that the actions of directed selves deviate from or are excessive or insufficient than expectations of directing selves (e.g., some behaviours of local authorities might be more excessive than what the central government expects). Third, cases in which consequences or outcomes of expectations of directing selves are either significantly better or worse than first planned by directing selves. Fourth, scenarios in which events selves strongly refuse to believe or wish against ultimately take place.

## 4. Implications for governance and research agenda for the future 

### 4.1. Implications for governance

***4.1.1. Reimagining selves***

The imaginaries of classical ethical philosophical writings are that human nature is binary and fixed, either benevolent (Mencius) or evil (Xunzi). The original position of human nature notwithstanding, it can be observed that they shared a belief that selves could be refined through education and ritual and through which deference and courtesy could be attained, thereby bringing about the proper order ^92^. Meanwhile, imaginaries of evil nature indicate the necessary recognition of the subsidiary role of law (legality) for primarily pure punishment purposes.

Classical economics shape the operation of society not on and around benevolence-evil but self-interests and rationality. “It is not from the benevolence of the butcher, the brewer, or the baker that we expect our dinner, but from their regard to their own interest. We address ourselves, not to their humanity but to their self-love, and never talk to them of our own necessities but of their advantages”, Adam Smith suggested ^93^. Human beings are imagined as rational, homo economicus who continuously search for self-interests. By acts of searching for self-interests of each individual, society can be motivated, operated, and flourished ^94^. The shift to ‘rational’ and ‘homo economicus’ depicts selves in more nuances and complexities: selves have various ‘base’ needs; these needs cannot be overlooked, should not be belittled, and need not be immoral. That also suggests the role of law in good governance beyond mere punishments. Good governance must recognize base needs, and allow them to be satisfied, but must simultaneously outlaw and sanction some in some situations. It must, thus, arrange institutions for base needs to be pursued and satisfied but constrain and prevent some.

Notwithstanding, imaginaries of classical economics concerning ‘homo economicus’ have also been challenged. Psychological and behavioural accounts also suggest that even where selves are motivated by expectations for self-interests, they cease due to some counter expectations or are overweighted by some other expectations. In many instances, behaviours are motivated not only and even not by self-interests but (also) by expectations for a better future and for legacies for future generations. Similarly, the imaginaries of classical economics concerning rationality have been challenged by economic accounts like those of Keynes, by behavioural accounts which recognize many biases and heuristics and schemas selves may have. That indicates that there can be wrongdoings formed upon expectations for the wrong as well as upon wrong expectations. That thus reshapes imaginaries about many violations and behaviours that are outright thought of as truly evil and unreasonable.

Meanwhile, selves, their needs, and desires are not shaped solely by certain innate traits but by and are constantly refined by the society in which they live, not only as it stands today but with legacies of the past and the legacies hoped to be passed on to the future, and social interactions.

It follows that a more fruitful way of imagining about selves is to imagine *selves as holders of expectations*. With that selves can be fully captured with nuances and complexities but without predisposition: Expectations may be base and irrational as well as excellent and though selves’ expectations may be base and irrational, they are real and neglecting these means either utopia or dystopia. Because it is not only that external context is a part of the package of conditions and reasons for expectations to be formed and thereby behaviours to be expressed but also that expectations, in turn, prompt behaviours and even social change, social and legal realities included, the concept of ‘expectations’ fully capture selves as selves with autonomy posing the capacity of making social changes, though these selves can possibly be wrong in certain instances, and selves within and in interactions with the large historical-social context rather than selves in isolation of context or selves as mere product of context that act only as proxy of contextual pressures or bounded selves.

***4.1.2. Governing around expectations***

The above subsection logically implicates those social institutions, for example, (bio)ethics and law, whose common aims are to dictate, regulate, or provide guidance for behaviours, to assist and guide decision-making concerning particular settings and contexts, and to shape realities, must pay attention to expectations. As demonstrated, ethics and law, conceding or not, have been informed by and have relied on ‘expectations’. What is ethical or not is, in many instances, influenced by and even shaped on and around expectations (or at least cannot neglect that phenomenon). Ethics as studies entail expectations for ethical behaviours (otherwise, ethics can be utopia). The law can regulate society precisely by imagining and expecting various behaviours which can potentially happen and provide dictations, demands, and guidance accordingly. The political community shaping the law (for some reasons) expects certain behaviours as being necessary, beneficial, or at least not harmful, which can be demanded or permitted. It also expects certain behaviours to be harmful, which must be prohibited, prevented (via certain arrangements), sanctioned, and coerced. The law then expects it to be obeyed by the population on which it rules and regulates, yet simultaneously expects the occurrence of deviants and arranges specific sanctions for specific deviants and specific procedural steps for the imposition of specific sanctions accordingly. Undoubtedly, the law, for it to effectively guide behaviours of selves, must pay attention to selves’ expectations, including concerns, and is responsive to these. Vice versa, for the law can effectively guide behaviours of selves, selves must have expectations on the law and form their expectations upon the law as opposed to, for example, force and violence. This entails, among other things, that attempts to promote human rights necessarily entail attempts to touch on and interact with the expectations of involved parties in relation to human rights. These logically entail that the law should constantly seek ways to enhance its administration of justice and expectations of its subjects on the law via the channel of the administration of justice beyond circumstances. It also follows that there is a point for ethical and legal studies to [continue to] pay attention to the concept of expectations not only to understand its operation, but also to forecast certain potential expectations in certain circumstances, to determine which expectations are appropriate, ethically and legally, to effectively regulate and give guidance to behaviours, particular in situations of crisis, to drive expectations towards being legitimate, and to facilitate and form norms appropriately corresponding to legitimate expectations. Meanwhile, in operating, institutional selves should maintain them to be *selves – holders to the totality of expectations of human selves establishing and maintaining institutions* - more than a proxy or tool for a particular self.

The concept of expectations (which represents the totality of interactions of selves and social context) also implies that the law and governance of one jurisdiction can learn from others, but for them to be really effective, solutions must be designed, with considerations paid to and tailored to historical and social accounts for reason that underlying social and institutional arrangements are different expectations and these in turn yield different expectations. Conversely, attempts to export or transplant a governance model or specific regimes or rules to different societies or generalize certain phenomena as a general theory of governance should be treated with caution.

### 4.2. Agenda for future research

As demonstrated above, the concept of expectation is nuanced, complex, and significant. Notwithstanding, its strength might also be its weakness. It can be inquired, for example, how can the concept and the framework it constructs be meaningful for operational and practical purposes?

It can be true that for a phenomenon or concept that is very complex or abstract, it can be hard – or even unfeasible - for it to be studied or comprehended overnight. But it is not the case here. Expectations have been studied by several disciplines and the method which this paper employs to conceptualize expectations is about synthesizing different accounts across disciplines. Similarly, in this line, the framework the concept constructs can be feasible and operational. Insofar as ‘expectations’ is a concept connecting selves and contexts and has been studied by different disciplines, it stands for the idea that is about bringing together different accounts of different disciplines which shall supplement each other for the purpose of better governance.

Recognizing the complexity which the concept of expectations and the framework that it constructs brings about does not mean denying the benefit of simplicity but opening a way to make use of it more effectively. This can be another way that the concept and the framework that it constructs can be implemented. Undoubtedly, it can never be fully comprehended of each and every self’s expectations; however, certain expectations can be formed as to what selves generally expect and generally expect in certain specific situations and what certain categories of selves generally expect and generally expect in certain specific situations. They are the techniques of categorization and rules of thumb. In light of these simplified expectations, responses, guidance, and norms can be prepared, facilitated, formed, and legitimized.

However, the concept of expectations also emphasizes that expectations are just expectations and it should be conscious of that fact. That means, in many instances, there are points to dismiss the ex-facie expectations and employ more nuanced expectations. As a result, contrary to ex facie impression, the strength of the concept of expectations does not lie in flawlessness but in imperfectness, reflection, and adjustment: unlike many concepts, theories, and frameworks that have fixed contents and are constantly seeking to justify their contents and frameworks, the concept of expectations recognizes different frameworks and their interactive contents as well as their shortcomings or inclinations of being fallible but observes these for practical purposes. That also signifies the need for more studies and the agenda for future research to better understand the real world and phenomena to provide fuller explanatory accounts and to calibrate sound solutions. For example, it can be presumed that officers can abuse powers but how is that possibility realised in different societies having different cultures and social structures? Which arrangements militate in favour or against the possibility of abuse of powers? This paper offers an observatory account that there is a tendency of lower-ranked officials to 'guess' the expectations of their superiors, but is that observation correct, to what extent is it correct, does that hold true in all societies and to what extent it is true across societies? How might [human] selves weigh expectations formed on informal institutions and interactions in comparison to expectations formed on formal ones? Different behavioural, historical, sociological and humanities accounts demonstrate these points and can supplement each other, for example, as can be observed in the earlier cited work of Rachel Wahl ^95^.

## Conclusion

This paper conceptualizes ‘expectations’ for the purpose of bioethical and health law studies. It defines ‘expectations’ as normative imaginaries that can induce or guide actions and inactions. It suggests that ‘expectations’ is a fruitful concept for bioethical and health law studies because it helps better understand the complexity of interactions in society and patterns of thinking and acting of actors in real-world contexts. This paper also suggests the implications the concept has for governance and an agenda for future research.

## Data Availability

No data are associated with this article.

## Footnotes

^1^ Tom Ginsburg and Mila Versteeg, ‘The Bound Executive: Emergency Powers during the Pandemic’ (2021) 19 International Journal of Constitutional Law 1498, 1534.

^2^ Cass R Sunstein, *This Is Not Normal: The Politics of Everyday Expectations* (Yale University Press 2021) 68–69; Charles de Secondat Montesquieu baron de 1689-1755, *The Spirit of the Laws* (New York : Hafner Pub Co, 1949 1949); Hai Thanh Doan, ‘The Public Health Response to COVID-19 in Vietnam: Decentralization and Human Rights’ (2023) 15 Asian Bioethics Review 103, 114.

^3^ Doan (n 2).

^4^ ibid.

^5^ Yu Insun, ‘Law and Family in Seventeenth and Eighteenth Century Vietnam’ (University of Michigan 1978); Samuel Baron and Hoàng Anh Tuấn, *A Description of The Kingdom of Tonqueen [Mô Tả Vương Quốc Đàng Ngoài]* (Omega Việt Nam 2019); Gustave Dumoutier, *Essais sur les Tonkinois* (Imprimerie d’Extrême-Orient 1908).

^6^ Li Tana and Nguyễn Nghị, *Nguyen Cochinchina: Southern Vietnam in 17th and 18th Centuries [Xứ Đàng Trong: Lịch Sử Kinh Tế Xã Hội Việt Nam Thế Kỷ 17 và 18]* (Trẻ 2013); Pétrus Jean-Baptiste Vĩnh Ký Trương, *Cours d’histoire Annamite à l’usage Des Écoles de La Basse-Cochinchine*, vol 1 (Impr du gouvernement 1875).

^7^ Insun (n 5).

^8^ Hai Thanh Doan, Diep Thi Phuong Doan and Nguyen Kim The Duong, ‘Post-Mortem Reproduction from a Vietnamese Perspective - an Analysis and Commentary’ (2020) 12 Asian Bioethics Review 257; Le Thi Lan, ‘Common Root of Three Religions (三敎同源) in History of Vietnamese Thoughts’ (2016) 36 Studies in Confucianism 535.

^9^ Đoàn Thanh Hải and Đoàn Thị Phương Diệp, ‘What Is Wrong with the Functionalist Approach Used by French Scholars in Studying “Điển”?’ (2023) 11 Comparative Legal History 45.

^10^ Lê Duẩn, *Cách Mạng Xã Hội Chủ Nghĩa Tại Việt Nam, Tập I [Socialist Revolution in Vietnam. Vol I]* (NXB Chính Trị Quốc Gia Sự Thật 1980). Cf writings of Hoàng Quốc Việt (Adam Fforde, ‘The Unimplementability of Policy and the Notion of Law in Vietnamese Communist Thought’ (1986) 14 Asian Journal of Social Science 60, 67.) Socialist rule of law and socialist rule of law-based State was adopted in the Seventh Congress of the Party and Resolution 51/2001/NQ-QH10 (Nghị quyết 51/2001/NQ-QH10 bổ sung điều của Hiến pháp nước cộng hoà xã hội chủ nghĩa Việt Nam năm 1992 [Resolution 51/2001/NQ-QH10 supplementing articles of the 1992 Constitution of the Socialist Republic of Vietnam] 2001. Article 51 The 1992 Constitution 1992; Truong Chinh, ‘Report to the National Assembly 12 December 1980’ (1980) 15 trans. FBIS East Asia Daily Report K15. Pham Duy Nghia and Do Hai Ha, *The Soviet Legacy and Its Impact on Contemporary Vietnam* (2018).

^11^ ‘Đẩy Mạnh Hơn Nữa Việc Phân Cấp, Phân Quyền Trong Quản Lý Nhà Nước [Further Promote Administrative Divisions and Decentralization in State Management]’ ( *Báo Điện tử Chính phủ*, 2021) < https://hcmcpv.org.vn/tin-tuc/day-manh-hon-nua-viec-phan-cap-phan-quyen-trong-quan-ly-nha-nuoc-1491886775> accessed 21 September 2022; Nguyễn Đăng Quế, ‘Một Số Vấn Đề Đặt Ra Trong Phân Cấp, Phân Quyền Giữa Trung Ương và Chính Quyền Địa Phương Hiện Nay [Some Issues Concerning Administrative Divisions and Decentralization between the Central Government and Local Governments Today]’ *Quản lý Nhà nước [State Management Review]* (13 May 2022).

^12^ Ginsburg and Versteeg (n 1) 1534.

^13^ ibid 1528.

^14^ “In Indonesia, several regional governments passed stricter regulations than the national government, prompting the President to issue a statement that “regional heads should not make policies of their own.” And in Colombia, Bogotá Mayor Claudia López resisted President Duque’s decision to allow some construction workers and manufacturers to return to work, prohibiting this in Bogotá, even without the formal authority to do so”.

^15^ Doan (n 2).

^16^ Directive No 16/CT-TTg dated 31 March 2020 which made lockdown possible by prescribing that *“... more than two people should not gather outside offices, schools…”*

^17^ Doan (n 2).

^18^ ibid.

^19^ Cong Giao Vu and Tri Uc Dao, ‘State of Emergency in Vietnamese Law: Reflections on the Government Response to the Covid-19 Pandemic’ (2022) 22 Australian Journal of Asian Law 5.

^20^ On 31 March 2020, Prime Minister Nguyễn Xuân Phúc signed Directive No 16/CT-TTg on current emergency measures to prevent and control the Covid-19 pandemic which suggests possible lockdown with a vague language – social distancing “Implementation of social distancing nationwide within 15 days from 0:00 am 1 April 2020, according to the principle that family isolates from family, villages isolate from villages and communes isolated from communes and districts isolated from the district, province, isolated from province ... more than two people are not to gather outside offices, schools, hospitals and in public places and main border gates, secondary border gates for passers-by on the border with Laos and Cambodia will be closed temporarily from 00:00 on 1 April 2020”.

^21^ Vu and Dao (n 19).

^22^ To satisfy Article 4.3 of ICCPR, a state - Vietnam - must inform the other States Parties through the intermediary of the Secretary-General of the United Nations of the list of the provisions from which it has derogated or wishes to derogate, accompanied by reasons.

^23^ See this argument in Bùi Thu Hằng, ‘Ban Hành Văn Bản Trong Tình Trạng Khẩn Cấp Nhìn Từ Dịch Bệnh Covid- 19 [Issuing Documents in a State of Emergency Observed from the Covid-19 Pandemic]’ (2020) 408 Nghiên cứu Lập pháp [Legislative Studies]; Cao Vũ Minh, ‘Pháp Luật về Tình Trạng Khẩn Cấp Của Liên Bang Nga và Những Gợi Mở Cho Việt Nam [Law on State of Emergency of the Russian Federation and Suggestions for Vietnam]’ (2021) 434 Nghiên cứu Lập pháp [Legislative Studies].

^24^ Under Article 3.1 of LPCDD class A consists of extremely dangerous infectious diseases that can transmit very rapidly and spread widely with high mortality rates or with unknown agents

^25^ Vu and Dao, *supra* note 19 at 11.

^26^ Concerning the judiciary, see *Id.* at 12–13; Pip Nicholson, *Renovating Courts: The Role of Courts in Contemporary Vietnam*, *in* A sian C ourts in C ontext 528 (2014); Trần Ngọc Đường, *‘Hiến Pháp Năm 2013 và Cơ Chế Kiểm Soát Quyền Lực Nhà Nước, Cơ Chế Bảo vệ Hiến Pháp’’’ [The 2013 Constitution and the Mechanism to Control State Powers and to Protect the Constitution]’*, *in* C Ơ chế Bảo hiến và quyền con ngƯời: kinh nghiệm C ộng hòa L iên bang Đứ c và V iệT N am [M echanism to protect the constitution and human rights: experiences of germany and V ietnam] (Cao Đức Thái ed., 2015). Concerning the National Assembly, only 14 out of around 500 members thereof are non-Party members.

^27^ John Ferejohn & Pasquale Pasquino, *The Law of the Exception: A Typology of Emergency Powers*, 216–217; Ginsburg and Versteeg, *supra* note 1 at 1512.

^28^ Section 3 Vietnamese National Assembly, *Nghị Quyết Số 30/2021/QH15 [Resolution No. 30/2021/QH15]*, (2021).

^29^ C arl S chmitt, P olitical T heology: F our C hapters on the C oncept of S overeignty (George. Schwab & Tracy B. Strong eds., University ed. 2005); Giorgio Agamben, *The State of Exception Provoked by an Unmotivated Emergency*, praxis, Feb. 26, 2020; R obert O. P axton, T he A natomy of F ascism (1st ed. ed. 2004).

^30^ Expect, M erriam-W ebster, https://www.merriam-webster.com/dictionary/expect (last visited Jan 11, 2023).

^31^ Some definitions are not relevant to the paper, e.g., to be pregnant.

^32^ Expectation, C ambridge D ictionary, https://dictionary.cambridge.org/dictionary/english/expectation (last visited Jan 11, 2023); Expectation, O xford L earners D ictionaries, https://www.oxfordlearnersdictionaries.com/definition/english/expectation (last visited Jan 11, 2023); Expect, O xford L earners D ictionaries, https://www.oxfordlearnersdictionaries.com/definition/english/expect (last visited Jan 11, 2023); Expect, *supra* note 30; Expect, C ambridge D ictionary, https://dictionary.cambridge.org/dictionary/english/expect (last visited Jan 11, 2023).

^33^ Expectation, *supra* note 32; Expect, *supra* note 32.

^34^ Expect, *supra* note 30.

^35^ *Id.*; Expect, *supra* note 32.

^36^ Expect, *supra* note 30.

^37^ *Id.*

^38^ Expect, *supra* note 32.

^39^ *Id.*

^40^ *Id.*

^41^ *Id.*

^42^ *Id.*

^43^ *Id.*

^44^ *Id.*

^45^ *Id.*

^46^ Expect, *supra* note 30.

^47^ For (1), the fact that one or more people ‘expect changes…’ do not mean there is any rule or obligation dictate as such. For (2), one can daydream and even get fancy results without working hard due to, inter alia, luck, working smartly, making use of the surrounding context and changes. For (3), in the context provided by the sentence, there is a match between the expectations of guests and the real facility of the hotel but in other instances there can be mismatches which neither indicate moral nor legal wrong.

^48^ Richard L. Oliver, *Expectancy Theory Predictions of Salesmen’s Performance*, 11 J. M ark. R es. 243, 244–245 (2018). *Id.*

^49^ M ankiw, P rinciple of E conomics 4 (9th ed. 2021). *Id.*

^50^ John Maynard Keynes, *The Principle of Effective Demand*, G en. T heory E mployment, I nteres. M oney 21, 22 (2018). *Id.*

^51^ J ohn M aynard K eynes, T he G eneral T heory of E mployment, I nterest, and M oney 134 (2018). *Id.*

^52^ K eynes, *supra* note 51 at xxxviii, 141. *Id.*

^53^ “animal spirits … a spontaneous urge to action rather than inaction, and not as the outcome of a weighted average of quantitative benefits multiplied by quantitative probabilities” (ibid, 141).

^54^ Mads Borup et al., *The Sociology of Expectations in Science and Technology*, 18 T echnol. A nal. S trateg. M anag. 285 (2006).

^55^ W.I. T homas & D.S. T homas, T he C hild in A merica: B ehavior P roblems and P rograms 571–572 (1928).

^56^ Robert K. Merton, *The Self-Fulfilling Prophecy*, 8 A ntioch R ev. 193 (1948).

^57^ *Id.* at 421–422.

^58^ Judee K. Burgoon & Jerold L. Hale, *Nonverbal Expectancy Violations: Model Elaboration and Application to Immediacy Behaviors*, 55 C ommun. M onogr. 58 (1988). *Id.*

^59^ Joseph Berger, Bernard P Cohen & Morris Zelditch, *Status Characteristics and Social Interaction*, 37 A m. S ociol. R ev. 241 (1972). *Id.*

^60^ Berger, Cohen, and Zelditch, *supra* note 59.

^61^ Cecilia L. Ridgeway, *Why Status Matters for Inequality*, 79 A m. S ociol. R ev. 1 (2014).

^62^ Mark L Flear, *Expectations as Techniques of Legitimation? Imagined Futures through Global Bioethics Standards for Health Research*, 8 J. L aw B iosci. 1, 2 (2021).

^63^ *Id.* at 1.

^64^ *Id.* at 3.

^65^ Mathilda Cohen, *The Rule of Law as the Rule of Reasons*, 96 ARSP A rch. für R echts- und S ozialphilosophie / A rch. P hilos. L aw 1, 12–13 (2010); Norimitsu Onishi, *Japan Learns Dreaded Task of Jury Duty*, T he N ew Y ork T imes, Jul. 16, 2007.

^66^ Flear, *supra* note 62 at 3.

^67^ Lord Templeman’s speech in Preston v IRC [1985] 2 All ER 327 at 341, [1985] AC 835 at 866–867; Nadarajah v Secretary of State for the Home Department [2005] EWCA Civ 1363 at para. 68, C.A. (England & Wales).

^68^ T eerawat W ongkaew, P rotection of L egitimate E xpectations in I nvestment T reaty A rbitration : A T heory of D etrimental R eliance 54–55 (2019).

^69^ Saluka Investments BV v. the Czech Republic (17 March 2006), UNCITRAL, point 304.

^70^ Ioan Micula, Viorel Micula, S.C. European Food S.A, S.C. Starmill S.R.L. and S.C. Multipack S.R.L. v. Romania, ICSID Case No ARB/05/20, Final Award (11 December 2013) para 671; El Paso Energy International Company v. The Argentine Republic, ICSID Case No ARB/03/15, Award (31 October 2011);

^71^ It is not that expectations are legitimate where circumstances facilitate or allow reasons for certain expectations to be formed, but rather expectations are only legitimate if they have certain intrinsic traits. It does not imply that morality can invalidate expectations or laws in each and every situation, however.

^72^ But for simplicity and because there is no difference between expectations concerning the two latter categories and the rest concerning selves, this paper groups and treat them together and similarly under the umbrella of the phrase ‘expectations of selves’.

^73^ Stanley Milgram, *Behavioral Study of Obedience*, 67 J. A bnorm. S oc. P sychol. 371 (1963); S tanley M ilgram, O bedience to A uthority: A n E xperimental V iew (1974).

^74^ J ames W aller, B ecoming E vil: H ow O rdinary P eople C ommit G enocide and M ass M urder 111 (Second edition. ed. 2007).

^75^ *See more* S. Alexander Haslam, Stephen D. Reicher & Megan E. Birney, *Nothing by Mere Authority: Evidence That in an Experimental Analogue of the Milgram Paradigm Participants Are Motivated Not by Orders but by Appeals to Science*, 70 J. S oc. I ssues 473 (2014).

^76^ Stanley Milgram, Leonard Bickman & Lawrence Berkowitz, *Note on the Drawing Power of Crowds of Different Size*, 13 J. P ers. S oc. P sychol. 79 (1969).

^77^ In 1955, following the announcement of the success of the polio vaccine trial, more than hundreds of thousands of children in the United States received a polio vaccine manufactured by Cutter Laboratories. Cutter’s vaccine contained live, infectious virus instead of inactivated virus. This caused 40000 cases of polio, leaving 200 children with varying degrees of paralysis and killing (Michael Fitzpatrick, ‘The Cutter Incident: How America’s First Polio Vaccine Led to a Growing Vaccine Crisis’ (2006) 99 Journal of the Royal Society of Medicine 156 </pmc/articles/PMC1383764/> accessed 17 February 2023).

^78^ The *“* Stanford prison experiment *”* suggests that people in positions of power might be prone to unethical behaviour and show less empathy and concern for the welfare of others. P hilip G. Z imbardo, T he L ucifer E ffect: U nderstanding H ow G ood P eople T urn E vil (1st ed. ed. 2007).

^79^ In Vietnamese society, there has been a shared imaginary of powerful and vice officials (mandarins) who could abusively manifest their powers to bend the law for their gains and oppress citizens. Before the pandemic, low-level authorities had already abused their powers in limited bureaucratic works. See, for example, Tu Phuong Nguyen, *Flexible and Compassionate, or Violent and Intimidating?: Various Accounts of Law in Vietnam*, 54 C rit. A sian S tud. 67 (2022). As low-level authorities had never possessed such unbounded powers, they might expect themselves to make all use of these.

^80^ R achel W ahl, J ust V iolence, T orture and H uman R ights in the E yes of the P olice 49 (2017).

^81^ Web Portal of Police of Binh Thuan Province, *Chiêu Bài “Dân Chủ, Nhân Quyền” - Mũi Tấn Công Thâm Độc Của Các Thế Lực Thù Địch [The Guise of “Democracy, Human Rights” - The Malicious Attack of Hostile Forces]*, (2022), https://congan.binhthuan.gov.vn/chieu-bai-dan-chu-nhan-quyen--mui-tan-cong-tham-doc-cua-cac-the-luc-thu-dich-N1115.aspx (last visited Jan 8, 2023). *Id.*

^82^ According to the famous *“* False Consensus Experiment”, if people hold certain beliefs or judgments, they may tend to expect that the majority of other people may hold the same beliefs or judgments (Lee Ross, David Greene & Pamela House, *The “False Consensus Effect”: An Egocentric Bias in Social Perception and Attribution Processes*, 13 J. E xp. S oc. P sychol. 279 (1977).)

^83^ Solomon Asch showed that a large majority of participants conformed to the group at least once though such an answer was incorrect. Solomon E Asch, *Studies of Independence and Conformity: I. A Minority of One against a Unanimous Majority.*, 70 P sychol. M onogr. G en. A pplied. 1 (1956).

^84^ For example, according to the principle of “ democratic centralism”, decisions of a high authority once made are binding for all subordinates and there are expectations of being disciplined should the latter refuse to follow orders. See Doan, *supra* note 2.

^85^ *Id.*

^86^ T he A nti-F ederalist, (Herbert J Storing & Murray Dry eds., 1985).

^87^ B urton W atson, T he A nalects of C onfucius (2007); I rene B loom & P hilip J. I vanhoe, M encius (Philip J. Ivanhoe ed., 2009).

^88^ Before Đổi Mới – the period when Vietnam decided to implement liberal economic policy, the party-state only recognized socialist legality. It is wrong to say there was no law in Vietnam at that time, rather legal documents were plentiful: Because Vietnam followed a Soviet Commanding Economy Model, periodically, the Government decided which goods to produce, which governmental agencies would be involved in the production, what would be the price of input and output goods, and even who would be distributed/sold goods and how much the volume of the distribution/sell would be for each individual. However, the law was just a discipline of the Party and an instrument of the state (D uẩn, *supra* note 10. Cf writings of Hoàng Quốc Việt (Fforde, *supra* note 10 at 67.). Laws have been amenable to being changed entirely regularly via administrative processes. Meanwhile, there was no place for “human rights”: Rights were understood as civic rights at best and inseparable from duties: the State only guaranteed civic rights as long as the citizens had fulfilled their duties first (Article 51 Vietnamese National Assembly, *supra* note 10; Chinh, *supra* note 10.). Socialist rule of law and socialist rule of law-based State was adopted in the Seventh Congress of the Party and Resolution 51/2001/NQ- QH10 (National Assembly, *supra* note 10.) The concept of “human rights” has been embraced. In light of the fact that laws are amenable to change entirely through administrative processes, for many scholars, a kind of human rights grounded in natural/moral rights is crucial for detecting the Rule of Law (as opposed to Rule by Law).

^89^ Martin T Orne, *Demand Characteristics and the Concept of Quasi-Controls*, 110 *in* A rtifacts in behavioral research: R obert R osenthal and R alph L. R osnow’s classic books 110 (Robert Rosenthal, Ralph L. Rosnow, & Alan E. Kazdin eds., 1 ed. 2009).

^90^ E lton M ayo, T he H uman P roblems of an I ndustrial C ivilization (Compass books ed. ed. 1966).

^91^ *Id*.; J ames M. H enslin, S ociology : A down to E arth A pproach 140 (Adam. Possamai & Alphia. Possamai-Inesedy eds., 1st ed. ed. 2010).

^92^ Bryan Van Norden, Mencius, in T he S tanford E ncyclopedia of P hilosophy (Edward N Zalta ed., Fall 2019 ed. 2019); Paul R Goldin, Xunzi, in T he S tanford E ncyclopedia of P hilosophy (Edward N Zalta ed., Fall 2018 ed. 2018). The different imaginaries lead to different implications for being good: For Mencius being benevolent is to exert one’s mind to the utmost know one’s nature [Mencius, 7A1], and to find “the lost mind” [Mencius, 6A11]. For Xunzi, what is good [in people] is their artifice (其善者偽也). Moral self-cultivation is not to overcome or abandon the xing (性), but to transform it (huaxing 化性).

^93^ A dam S mith, A n I nquiry into the N ature and C auses of the W ealth of N ations 7 (J. R. (John Ramsay) McCulloch ed., 4th ed., corr. an... ed. 1850).

^94^ *Id.*; B ernard M andeville, T he F able of the B ees: P rivate V ices, P ublick B enefits. (Irwin Primer ed., 1962)

^95^ W ahl, *supra* note 80.
