# Expectations and Vietnam’s responses during COVID-19: potential human rights violations and related propositions

**DOI:** 10.12688/wellcomeopenres.18972.4

**Published:** 2025-05-13

**Authors:** Hai Doan

**Affiliations:** 1 Bioethics Centre, University of Otago, Dunedin North,, Dunedin, 9016, New Zealand

**Keywords:** expectations; expect; human rights; COVID-19 pandemic; emergency powers; Vietnam; the state of exception.

## Abstract

**Background:**

It appears that underneath many propositions or actions, there might have been certain expectations. The reality of the complexity of human beings and interactions within a society yields expectations which, in turn, further interact with each other.

**Methods:**

This paper offers the first in-depth study on ‘expectations’ in bioethical and health law studies, taking Vietnam’s responses to coronavirus disease (COVID-19) as an illustration.

**Results:**

It defines ‘expectations’ as normative imaginaries that can induce or guide actions and inactions at individual and collective levels.

**Conclusions:**

It argues that violations of human rights and certain related propositions within the Vietnamese context can be understood in terms of expectations. Within such contexts as Vietnam, expectations account for behaviours, including human rights violations. It suggests that ‘expectations’ is a fruitful concept for bioethical and health law studies. Studying expectations thus has implications for governance and future research agendas.

## 1. Introduction

Some scholars expect that local actors can help to check the power of the central government ^1^. There is a general expectation that local actors are capable of calibrating policies, responding to local needs, conditions, and preferences, as well as reducing abuses to a lesser extent ^2^. Within the Vietnamese context, that kind of expectation justified the delegation of powers – the process in which higher authorities delegate power to lower ones to make and implement public health measures – during the COVID-19 pandemic ^3^. Yet, the delegation of power fermented conditions for unbounded powers of local officials, thus leading to abuses of powers and possible violations of rights ^4^. But why were there human rights violations if such violations are ethically horrendous, especially considering that some behaviours even seem strange and unreasonable? Amidst that context, some Vietnamese scholars expected the declaration of the state of emergency, but why did scholars expect so? That is to ask, how could the declaration of the state of emergency make a difference and/or how could the expectation be understood as making sense?

It appears that underneath many propositions or actions, there might have been certain expectations. The reality of the complexity of human beings and interactions within a society yields expectations which, in turn, further interact with each other. ‘Expectations’, thus, is a significant concept to address the patterns of thinking and acting of subjects and society. This holds true across societies but, of course, is more observable in certain ones. Unfortunately, this concept is under-conceptualized in bioethical and legal studies. This paper conceptualizes ‘expectations’ and indicates some incidental implications. This paper defines ‘expectations’ as *normative imaginaries that can induce or guide actions and inactions*. The conceptualization is done using the analytical method of interpreting existing definitions, gathering relevant accounts across disciplines, and using cases, particularly responses to the COVID-19 pandemic within the context of Vietnam, together with other cases. Conversely, the conceptualization of expectations demonstrates how human rights violations and certain related propositions can be understood in light of expectations. The line of logic in being extended thus implies that within such contexts as Vietnam, expectations account for behaviours, including human rights violations. The paper suggests that the concept of ‘expectations’ has implications for governance as well as research. 

The rationale for Vietnam being chosen as an illustration boils down to not exclusively Vietnam being an under-studied jurisdiction but also an interesting case study. Historically, the mode of governance in North Vietnam was a mixture of family-based, village-based and regional-based ^5^. In South Vietnam, the governance structure was quite similar but looser and more liberal for the reason that people there were originally from different regions and of different origins and came there as settlers ^6^. In brief, Vietnam had a governance structure featured by ‘local units’. Central dynasty courts attempted to centralize powers but were not quite successful in general ^7^. Culturally, being influenced by Confucianism, social norms played a crucial role (Analects 2.3). Notwithstanding, Confucianism is not the only school of thought in Vietnam ^8^. Thus, social interactions in Vietnam are rich ^9^. In the modern time, Vietnam is a one-party state. Its governance structure was modelled after the Soviet Union. That signifies, among many things, the bureaucratic nature, a centrally-focused governance structure, the submission to absolute powers, the deemphasizing of human rights ^10^. With the collapse of the Soviet Bloc and economic hardship, Vietnam opened to liberal order and has been in transition, signifying a welcome to the Rule of Law, human rights and decentralization - the transferring of more powers to local actors ^11^ while retaining bureaucratic and rudimental legal and governance structure. Thus, various transitions and struggles between factors can be observed, and as an inevitable consequence, expectations are socially significant.

The second section offers some cases within the context of Vietnam and makes an initial observation that there were certain expectations borne by certain actors. The third section conceptualizes ‘expectations’. The conceptualization is done using the analytical method by which we interpret plain meanings of expectations provided in/by dictionaries and induce them into the concept of expectations. Then, the concept is demonstrated as embedded in the various disciplines’ understanding of expectations. Though the list of disciplines where the concept of expectations is employed can be longer, this section illustrates disciplines whose findings may be observed as having connections with the ‘thread’ of this paper (so as to keep the paper focused and sections linking together coherently). Understandings of various disciplines validate and reinforce the conceptualization of ‘expectations’ of this paper. Thus, this allows an interpretation that understandings of various disciplines concerning expectations can be deduced from our conceptualization, and vice versa, the conceptualization can still be achieved by inducing from these understandings. That allows the process of conceptualization to be hermeneutic. Following that, this paper then highlights certain features of expectations. Then, with the concept of expectations (and its features) as a backdrop, the paper revisits Vietnamese cases described earlier to demonstrate that expectations account for violations of human rights and certain related propositions within the Vietnamese context. This allows takeaways from Section 3.1– Section 3.4 to be useful to shed light on such patterns of behaviours. To concretize the points and provide a more comprehensive view and convincing argument, cases (legislation, case law, reports) in relation to COVID-19 in certain jurisdictions, namely, the UK, New Zealand, and Taiwan, are added. This reinforces one again the relevancy of expectations and demonstrates common expectations and expectations especially salient within the context of Vietnam. This also reinforces the conceptualization of expectation in earlier subsections. Building on the analysis of Section 3 (particularly Section 3.5), the fourth section explores some implications of the concept of expectations for governance and research. The first subsection of Section 4 suggests general observations concerning the relevancy of expectations to ethics and law. The second subsection suggests implications of the concept of expectations for such jurisdictions and the studies concerning such jurisdictions as Vietnam.

For clarity, this paper deploys phrases ethics and law and/or rights together to signify that they are not synonymous but should be simultaneously considered in relation to a particular issue.

## 2. Cases

### 2.1. Local powers vs central powers

In the paper titled *‘The bound executive: Emergency powers during the pandemic’*, studying different jurisdictions, Tom Ginsburg and Mila Versteeg suggest various ways according to which ‘executive power’ can be claimed to have been bounded during COVID-19 pandemic. Their imagination of ‘executive power’ is, however, only ‘central executive over-reach’ ^12^. Perhaps, upon this account, they phrase local actors as ‘subnational constraints’, *applicable to a wide range of jurisdictions* ^13^. ‘Subnational constraints’ and their actions, including ‘resistance to central government directives’, ‘more restrictive approach than that of the central government’, ‘ignorance of central government’s directives’ seem to be endorsed. The term ‘subnational constraints’ and the overall tone of writing ^14^ suggest that they saw such actions as positive. But why is ‘bounded executive power’ constrained upon the central government’s power but not local governments, and why is disobedience or negligence (even unlawful) of local governments against policies of the central government executive constraints? Aren’t the powers of local government also executive? Or cannot local government’s executive powers pose threats to citizens? Thus, can it be expected that local authorities are ‘subnational constraints’? That also illustrates a general expectation that local actors are capable of calibrating policies and responding to local needs, conditions, and preferences ^2^.

### 2.2. Possible human rights violations ^15^ by local authorities in Vietnam during the COVID-19 Pandemic

There can be no more salient case study that gives rise to the above questions than the Vietnamese situation. Within the Vietnamese context, perhaps the kind of expectations mentioned in the above case justified the delegation of powers to and wide discretion of local authorities to make and implement public health measures during the COVID-19 pandemic ^3^. However, with - and even without - formal delegation of powers, local governments established checkpoints in their ‘own territories’ and invented significantly divergent rules for their own ‘fortresses’. For example, in some localities, entry of outsiders (people who do not reside there) was banned; in some other places, people were required to test at checkpoints (all other test results, even those provided by previous checkpoints within the previous few seconds, would not be accepted). Within that context, local governments might be possible sources of human rights violations. ^16^ It is reported that local officials have locked citizens' houses with citizens inside, worrying that without such ‘lock-up’, citizens might go out and spread the disease. Tellingly, it is reported in a case (the “ *Bread is not Food”* case), officials first ‘taught’ a citizen that Directive 16 ^17^ only allowed citizens to go out for essential purposes, while buying food was not; such officials then alternatively argued that “bread is not food”. ^18^ In another instance, citizens were coerced by force to take a COVID-19 test administered by public officers. ^19^ But why did officials – *in plural* - commit possible human rights violations if such violations were manifestly ethically horrendous and unreasonable?

### 2.3. Declaration of emergency

Amidst that context, some Vietnamese scholars believe that the central government should formally declare the state of emergency. This can be observed, for example, in the statement of Vũ Công Giao and Đào Trí Úc – two prominent scholars in the area of constitutional law and socialist rule of law-based state ^20^:

*“As to the misuse of emergency powers, Directive 16 actually caused human rights derogation without declaration of a state of emergency as required by the 2013 Constitution”* ^21^.

It is worth noting that on 31 March 2020, Prime Minister Nguyễn Xuân Phúc signed Directive No 16/CT-TTg on current emergency measures to prevent and control the Covid-19 pandemic which suggests possible lockdown with a vague language – *social distancing*:

“Implementation of social distancing nationwide within 15 days from 0:00 am 1 April 2020, according to the principle that family isolates from family, villages isolate from villages and communes isolated from communes and districts isolated from the district, province, isolated from province... more than two people are not to gather outside offices, schools, hospitals and in public places and main border gates, secondary border gates for passers-by on the border with Laos and Cambodia will be closed temporarily from 00:00 on 1 April 2020”.

By mentioning ‘misuse of emergency powers’, describing many potential violations of human rights, and resorting to the discourse of human rights, they expressed their concerns for emergency powers and human rights. That is, they expect a declaration of a state of emergency, the use of emergency powers, and certain effects or implications brought about by such a declaration.

## 3. Expectations

It can be observed that there are expectations in all the above cases. Perhaps the reason for which expectations appear in all cases is simply that except for someone who loses the capacity of cognition and perception, expectations as belonging to or presenting cognitive ability must exist. As this section shall demonstrate, expectations represent human innate tendencies and social experiences.

### 3.1. Definition of expectations

‘Expect’ or ‘expectation’ are words used popularly in daily life. Generally, dictionaries for English users define ‘expectation’ or ‘expect’ ^22^ with the following meanings ^23^:

1) A belief, or to think or believe, something will happen ^24^
2) A belief or to think that someone should behave in a particular way or do a particular thing ^25^ or to consider reasonable, due, or necessary ^26^ or to consider bound in duty, responsibility or obligated ^27^.
3) To suppose/a supposition ^28^
4) To await/ awaiting ^29^.

Meaning (2) seems to suggest that expect/expectation may appear as a counter-partner of duty or obligation, but in scrutinizing examples offered by dictionaries, it is not always the case. Examples provided by dictionaries are as follows:

- It is reasonable to expect changes in the way we work ^30^. (1)
- No one has a right to expect good results without working hard ^31^. (2)
- These are the high standards that hotel guests have come to expect ^32^. (3)
- He's still getting over his illness, so it's unrealistic to expect too much from him ^33^ (4).
- Are you clear what is expected of you? ^34^ (5)
- You can't reasonably expect people to pay such high taxes ^35^ (6).
- We are expected to work on Saturdays ^36^ (7).
- I expect to be paid promptly for the work ^37^ (8).
- They expect you to pay your bills ^38^ (9)

From (1) – (3), the sense of legal or moral obligation or responsibility or duty is vague ^39^.

For (4) and (5), subjective expectations from one side need not express a clear sense of being bound by duty, being obliged or obligated, being mandated, or incurring responsibility from the other side. The other side may not be aware of or may dismiss such expectations.

For (6) and (7), there are contradictions in the sense of being bound. In (6), there can be an obligation under the law that can be irrational or morally invalid. It is the opposite in (7), a feeling of being morally obliged to work extra time can be contrary to the law.

For (8) and (9), the actions at stake can be negotiated, subject to various contextual factors without implying moral or legal wrongs. Consequently, these examples might suggest that certain actions are desired or looked for (irrespective of who carries out such actions).

It would follow from what has been demonstrated that ‘expect’ and ‘expectations’ can also be understood as *“to look for”* or *“to desire”* or *“a desire or an ambition*” and *“to hope”* or *“a hope”*. As a result, ‘expects’ or ‘expectations” do not refer to mere ‘acts of believing’ or ‘beliefs’ in a neutral sense, but ‘to believe’ with or ‘beliefs’ having normative propositions or judgements. Underlying the normative beliefs are certain imaginaries.

It would follow from the above that ‘expectations’ can be defined as *normative imaginaries that can induce or guide actions and inactions (generally ‘actions’).*

‘Expectations’ has long been an important concept for many intellectual fields and theories, which aim to offer explanations for a range of social phenomena and issues. Different understandings of disciplines concerning expectations link to each other to a certain extent and can supplement each other. It can also be observed from the investigation of these understandings that the concept of ‘expectations’ as conceptualized above is embedded in these understandings. That is, though various learnt fields and theories conventionally refer to ‘expectations’ in light of one or other meanings for the purpose of such theories or fields, the essence of these understandings is what this paper conceptualized above. That validates and reinforces the above conceptualization of ‘expectations’. Thus, it allows an interpretation that understandings of disciplines concerning expectations can be deduced from our conceptualization and vice versa, the conceptualization can still be achieved by inducing from these understandings.

In the ‘expectancy theory of motivation’ in organizational behaviour, expectations - the *subjective probability* of the attainment of rewards - are *incentives* that motivate employees’ job performance ^40^. That is, people respond (or are motivated), to a certain degree, to their guess/guesstimate of rewards they can receive. Yet, it is also expected that people will gradually cease to respond to such rewards at some point or under some conditions where they expect otherwise. In short, *expectations are beliefs and practical reasons*.

‘Expectations’ is a core concept in economics and shows its intriguing significance therein. Classical economics studies claim that expectations are among the factors that drive decisions (i.e., *practical reasons*). For example, expectations about the future may affect both demand and supply for a good or service today ^41^. But that may simplify the matter too much. Keynes recognized that a practical decision would not entertain a single undoubting expectation, but *several hypothetical expectations,* held with *varying degrees of probability and definiteness* ^42^. Our own preferences are not as important as the anticipation of *“what average opinion expects average opinion to be”* ^43^, i.e., expectations of an individual about the market are expectations of the average expectation of the market which is the average of the sum of individuals’ expectations. Keynes also recognized that though expectations would be shaped partly by knowledge and creativity, they could be unstable ^44^ and sometimes irrational, influenced by *‘animal spirits’* ^45^. Keynes, thus, suggested that *expectations are not only various, interactive and adaptive but can also be unstable, irrational, and wishful and can also even be a determinant of realities*.

Perhaps not by coincidence, there is a kind of imagination of *‘animal spirits’* and ‘ *a determinant of realities’* in science and technological sociology studies. Expectations as *wishful enactments of a desired future* are claimed to underline bold advancements ^46^. These accounts have certain commonalities with the Thomas theorem: “If men define situations as real, they are real in their consequences” ^47^ and the self-fulfilling prophecy in socio-psychological studies: “men respond not only to the objective features of a situation, but also, and at times primarily, to the meaning this situation has for them. And once they have assigned some meaning to the situation, their consequent behaviour and some of the consequences of that behavior are determined by the ascribed meaning” ^48^.

In sociology, according to the ‘expectancy violations theory’, expectations refer to *social norms* and *judgements concerning common patterns of behaviours in a given society that actors are expected to follow* ^49^, which impact the attitude of individuals vis-à-vis actions of other individuals and those individuals. According to the ‘Expectation states theory’ ^50^, expectations refer to some kinds of *practical beliefs*, i.e., beliefs about practical issues, and even *stereotypes,* which form status hierarchies ^51^ and social inequality ^52^. It follows that these studies suggest that *expectations can be both norms and mechanisms to implement norms (coercion and sanction, formal and informal*, including temptations to meet expectations). Meanwhile, *the realities that expectations facilitate include the systems of norms.*

Certain aspects of each of the above theories are captured by ‘the Theory of Planned Behavior’. Accordingly, the interaction between behavioural beliefs, which are yielded by the expectations of outcomes, with control beliefs, which are the expectations of the ability to perform behaviours and normative beliefs, which are expectations of attitudes of peers and respected figures toward the behaviours, forms the precise intention and behaviours ^53^. 

In law, expectations have been acknowledged as relating to various matters though it is argued to be a relatively novel concept in bioethics and health law ^54^:

    (i) *The facilitation, formulation, and legitimation of legal norms and governance:* Mark L. Flear asserted that “expectations … can be shaped by organizations and used by them as techniques for public legitimation of their governance and regulatory activities” ^55^. Meanwhile, weighing positive expectations (benefits) and negative expectations (concerns and risks) provides a basis for legitimizing new science and technology, for informed consent to treatment, and for human participation in health research. ^56^

    (ii) *The frustration of legal norms and governance:* It has been found that expectations formed within a social context and influenced by social norms can distort and jeopardize legal initiatives and implementations of legal provisions. For example, it has been argued that the initiative to introduce jury trials in Japan in criminal trials would be destined to fail in light of expectations of lay citizens towards experienced judges and expectations to not argue with one another and to question authority ^57^.

    (iii) *The protection of legitimate expectations:* ‘Legitimate expectations’ is an important concept/doctrine in law and legal studies. ‘Legitimate expectations’ are grounded not only on promise ^58^ but ‘good administration of justice’ ^59^. Legitimate expectations are inherently linked to justice and fairness, reasonableness, and ethics ^60^. Expectations are legitimate and legally protected only if they are reasonable in light of the circumstances ^61^ and must not be undue or illegal ^62^.

These theories suggest features and operation (both the mode and implications of operation) of expectations, which shall be explored in subsequent subsections.

### 3.2. Subjects and objects of expectations

Several objects can give rise to expectations: persons (capturing both individuals and entities, the plurality includes the singularity), institutions (formal and informal), and manifestations (real, potential, or unreal, events included). Institutions are distinct and independent from persons (who invent and operate the institutions), and manifestations are mentioned in the sense emphasizing the manifestations themselves as opposed to the agency that performs or induces such manifestations. Meanwhile, only persons and institutions (generally called selves) are capable of holding expectations. It can be put differently that agents are capable of expectations which are directed toward their environment which is a subset of surrounding subjects and objects. Conversely, they also receive, perceive, or interpret expectations of other agents.

### 3.3. Some key characteristics of expectations

***3.3.1. The variety***

Expectations vary among selves. All selves have their own expectations, drawing from not only selves’ personalities, past experiences, skills, knowledge, and confidence but also the purposes of these, contexts (i.e., social interactions and expressions), and components of selves. Such expectations may change from time to time. They are various in terms of issues, contents and the associated degree of desirability: Expectations are not only about ‘trust/believe or not’. In certain instances, various expectations of a given self at a time point may be in tension or contradictions with each other.

***3.3.2. The rational vagueness***

Expectations can be rationally vague. In saying so, this does not mean that expectations are neither rational nor irrational, but that they may receive a continuum of values with ‘rational’ and ‘irrational’ at polar, depending on various factors. Whether specific expectations are rational (and to what extent) depends not only upon subjective assessments of that given subject but also on “counter-expectations” and assessments of other actors. Expectations are also rationally vague in the sense that they are trans-factual: ‘Unrealistic’ expectations may sometimes result in unimaginable outcomes, good or bad, or help to achieve all or some unrealistic targets but sometimes are counterintuitive.

***3.3.3. Moral vagueness***

Expectations can be consequential (inclination to rewards, pleasure or aversion of pains or punishment or risks or disrepute or losses) or deontological or just authority-oriented or herd-oriented (i.e., unidentified moral values). Expectations of selves thus can be clearly moral or immoral or morally uncertain, e.g., in case of dilemmas, in times of uncertainty. These points are illustrated by two experiments conducted by Stanley Milgram.

The first experiment is ‘Obedience to Authority’, aiming to investigate how far individuals would go in obeying an authority figure's instructions, even if that may cause harm to other persons. The experiment is as described: The volunteer arrived at the laboratory setting where that person was assigned the role of ‘teacher’ and met two other persons: an actor falsely self-introducing as another volunteer and being assigned the ‘learner’ role and the third member of the triad dressed in a gray technician's coat. This third member then “explained” to the other two that the study they are working on is concerned with “the effects of punishment on learning” that the “learner” would be taken to an adjacent room, strapped into a chair, and an electrode is attached to his wrist. The real volunteer – the “teacher” is seated in front of “an impressive shock generator,” on which there are 30 switches ranging from 15 to 450 volts and accompanying written designations ranging from “SLIGHT SHOCK” to “DANGER—SEVERE SHOCK.” The “teacher” was instructed to administer a verbal learning test to the person in the next room, and whenever an incorrect answer was given, the “teacher” gave the other an electric shock in return. In that experiment, nearly two‐thirds of the “teachers” or subjects kept pressing away to 450 volts—and when in one modified form of the experiment the further hoax that the “learner” had a bad heart was introduced, and when he began to shout and scream about pains in the chest, it made no difference at all. Sixty‐five per cent kept pressing the switches. It suggests that (i) ordinary people may expect themselves to follow orders even when doing so makes them agents in a terrible destructive process ^63^; (ii) ordinary people may expect authorities to be truthful. There are explanatory accounts for the behaviours of ‘teachers’ as such behaviours were due to expectations that ‘learners’ would not be permanently damaged, formed upon reassurance from the experimenter ^64^. Notwithstanding, such expectations, if they existed at all, would neither be rational nor ethical: People without physics knowledge can still expect that 300–450V would be harmful and even deathly; nor are they supported by contextual factors (screams of learners); (iii) wrongdoings, immoralities, and harms can predicate upon expectations of a singular fantastic cause while overlooking related surrounding possible ethical concerns and peripheralizing other interests ^65^.

The second experiment is known as the gaze-following effect ^66^. In this experiment, at a signal, a group of confederates (stimulus crowd) entered the middle of the observation area, stopped, and looked up at the sixth-floor window for 60 seconds ^66^. At the end of this period the group was signaled to disperse ^66^. After the area was cleared of the gathered crowd, the procedure was repeated using a different size stimulus, crowd ^66^. The study demonstrates that the probability of a person following the stimulus group’s action increased with the size of the stimulus group. It seems to be the case that people may imitate behaviours of other people (a crowd) and expectations can be formed upon or in response to the behaviours of others.

Conversely, expectations play a role in determining decisions or actions by expecting moral values of these decisions or actions once implemented. For practical purposes, the debate on vaccine mandates serves as an example. Proponents of vaccine mandate campaigns may expect a scenario in which, in the absence of wide coverage of vaccination, millions of people may die excessively and the society shall be discontinued, but in the absence of a vaccine mandate, a significant portion of the population may refuse vaccination. Those expectations give grounds for expectations for moral values of vaccine mandate, especially in the absence of free consent. Opponents of mandatory vaccination expect that people are rational and shall choose what is optimal for them or that the vaccine is ineffective or even defective, recalling the ‘Cutter Incidence’ ^67^, or at least dangerous to certain people who cannot be predetermined, however. Whether an authority accepts the moral and legal values of the vaccination mandate is subject to the weighing of expected associated benefits and costs ^68^.

***3.3.4. Expectations are ambiguous***

Expectations are ambiguous. First, selves may be not clear what they expect for or expect for the most. Also, their expectations may be in tension or contradictions with each other. Second, as a corollary of the first and/or due to the complexity of contextual factors, the decoded messages conveyed by the signalling expectations are vague, thus making ‘counter-expectations’ formed upon such messages vague: A self may receive various messages that they try to interpret, and the interpretation process results in various contradictory imaginaries of signalling expectations and thus leading to contradictory counter-expectations. Sometimes, this vagueness of counter-expectations is accidental or due to poor interpretation skills, but sometimes it is intentional. Perhaps, this tendency is due to, first, the human instinct that prefers decoding something complex to just reading public statements or the confidence in privileged intelligence over common knowledge. Second, sometimes, high authorities or sovereignties or rulers or bosses do not want to outspeak their position and thus express themselves vaguely and incomprehensively by different contrasting statements. A possible reason for this is that they want to maintain themselves as considerate selves ^69^. Unpublicized and hidden messages are observable phenomena and are salient in some societies. It thus follows that, thirdly, guessing what is in the mind of a boss, high-ranked official, ruler, sovereignty (“Ðoán ý”) is not a rare phenomenon in certain societies and can be observed throughout history and empirically. These imply that to the extent that two messages an actor received clash, the actor may be inclined to opt for the message that the actor is confident shall minimize risks and harms on his or her side. It would follow that what local actors expect as expectations of the central government for them has much more gravity than what the central government addresses publicly. This can be observed with the tragic “Great Leap Forward Campaign” in which local officials fearful of Anti-Rightist Campaigns left farmers to starve to death or with repeated concealment of SARS and COVID-19 outbreaks in China.

***3.3.5. The contextual impacts***

Not only internal traits, e.g., genes, brain, and nerves, and mental biases and shortcuts but contextual factors, i.e., social roles, social interactions, or social expressions, play a significant role in expectation-formation. Expectations are shaped by and/or respond to context, and context aggravates and relieves certain instinctive tendencies. Tom Ginsburg and Mila Versteeg’s expectations seem to be understandable within the context of the US (where they reside), where there have been worries about the potential tyranny of the central despot or Leviathan ^70^, which prompted Publius (the alias standing for Alexander Hamilton, John Jay and James Madison) to publish a series of essays to defend federalism. Powers were delegated by the people to the central government and local governments in parallel, but powers were still intended to reside largely in local governments which hold general jurisdiction ^71^.

### 3.4. Interactions of expectations

Because society is a web of interactions between selves, expectations do not exist in a vacuum or singularity but in a web. It is to say that expectations may direct or respond to or cause other expectations.

***3.4.1. Signalling expectations and counter-expectations***

As expectations are a subjective state of mind, selves need not be triggered (by other expectations (‘signalling expectations’) of other selves directed at the former selves or by stimulus) to form expectations. Notwithstanding, expectations are also formed in response to signalling expectations. Expectations formed in response to signalling expectations are called ‘counter-expectations’. However, counter-expectations are not direct products of signalling expectations. Rather, counter-expectations are products of subjective interpretations or imaginaries of meanings of a complex web of factors, among which signalling expectations are a factor. As a result, expectations to respond to expectations are more accurately framed as expectations to respond to presumed signalling expectations. Such interpretations of messages upon which counter-expectations rely and form are subjective and can be false - if there are any signalling expectations at all.

***3.4.2. States of interactions***

*Meeting expectations:* Selves may try to meet the expectations of other selves, especially those of higher authority. In this pattern, presumed signalling expectations are interpreted as normative and authoritative.

*Failing expectations:* Expectations can be failed in certain circumstances, for example, when signalling expectations are missed or misread, or when though signalling expectations are interpreted accurately in total or in substantial part, attempts to achieve what is expected or to satisfy the needs behind the signalling expectations fail. Failing expectations may result in punishments or unfavoured treatments from selves emancipating signalling expectations.

*Evading and resisting expectations:* Sometimes, selves may attempt to evade or resist expectations directed upon them should there be conflicts or clashes in expectations between the former selves and selves sending signalling expectations or between expectations of the latter with any third parties who are more mattered to the former. The “bank wiring room experiments” suggest that despite payment incentives, productivity decreased due to other expectations of workers, for example, that their productivity may have been boosted to justify firing some of the workers later ^72^.

*Beyond expectations:* There are several layers of meanings for ‘beyond expectations’. The standard understanding of the phrase provided by some dictionaries is that “act is more than people thought would be the case”, usually with positive connotations or approval. There are other layers as well. First, cases in which expectations are directed on selves (directed selves) by other selves (directing selves), and it is either that the actions of directed selves deviate from or are excessive or insufficient than expectations of directing selves (e.g., some behaviours of local authorities might be more excessive than what the central government expects). Second, cases in which consequences or outcomes of the realization of expectations of directing selves are either [significantly] better or worse than first planned by directing selves or the selves implementing actions. Third, consequences or outcomes of the realization of expectations are entirely not expected. Fourth, scenarios in which manifestations selves strongly refuse to believe or wish against ultimately take place.

### 3.5. Expectations in Vietnamese cases

After conceptualizing ‘expectation’, this subsection shall analyse and demonstrate that expectations accounts for human rights violations and related propositions within the Vietnamese context, as mentioned in the above cases.

***3.5.1. Central government***

As mentioned, selves, namely the governments and authorities, are capable of establishing expectations via their behaviours. This, of course, has been recognized in many instances as well. For example, it can be read from the opinion of the International Court of Justice in the case ‘ *Nuclear Tests*’ ^73^ that in light of the behaviours of the President of the Republic of France, there was an expectation that France had an intention and shall honour the intention of terminating the nuclear tests, thus absolving the case. In another well-known case - SPP v. Egypt, it was recognized that under Egypt’s domestic law, certain decisions by certain high-ranking Government officials “which are now alleged to have been in violation of the Egyptian municipal legal system, created expectations protected by established principles of international law” ^74^. Amidst the context of the COVID-19 pandemic, the High Court of New Zealand in the case Borrowdale ^75^, after examining various factors including the Prime Minister’s role at the time with the full authority of her office and the State ^76^, the wider context: the factual reality that the country then faced ^77^, the awareness of the legal enforcers ^78^, the hardship that may be yielded incidental to the proposition that the mere education of the public is capable of yielding the desired outcomes ^79^, the Court concluded that Statements of the NZ Prime Minister created the overwhelming impression that compliance was required by law and thus was not guidance but legal commands ^80^. The commands ultra vires restricted rights protected under the New Zealand Bill of Rights Act 1990. Any limits on rights that were *implicit* in the Restrictive Measures were not prescribed by law ^81^.

Following these instances, there are ample reasons to believe that within the context of Vietnam, though Prime Minister Nguyễn Xuân Phúc in issuing Directive 16 claimed that it was neither a lockdown nor a traffic ban and warned against divergent, inconsistent, and excessive measures of local authorities ^18^, the Central Government expected local authorities to legislate and employ any measures they deemed fit at whatever costs. *Alternatively,* even notwithstanding the precise de facto expectations of the Central Government, the conjecture and interpretation of the Central Government’s expectations that the local authorities could legislate and employ any measures they deemed fit could be objectively and reasonably drawn.

*First,* the Government forcefully required that local authorities’ measures must be ‘ *one-level higher’ and ‘one-step earlier’* than the measures of the Government and left the whole matter or discipline to whatever behaviours of local actors.

*Second,* the matter is further elaborated by the legal framework itself. Article 53 of the Law on Prevention and Control of Contagious Diseases (LPCCD) concerning ‘Control of entry into and exit from class-A epidemic zones’ permitted in Clause 1 Point d ‘Các biện pháp cần thiết khác theo quy định của pháp luật’ (Other necessary measures as prescribed by law). The concept of ‘pháp luật’ (law/legal) itself within the Vietnamese context is [worrisome] over-broadly defined, comprising not only primary legislations such as LPCCD itself, secondary legislations, e.g., any kinds of regulations and circulars, but administrative decisions that have a general scope of applicability ^82^. Generally, local authorities can enforce whatever measures they deem fit ^83^. 

*Third,* the Vietnamese National Assembly did authorize the Vietnamese Government and local authorities with *a blanket authorization* under Resolution No. 30/2021/QH15 dated 28 July 2021, again, to [legislate and] employ any measures they deem fit ^84^.

*Fourth,* in most instances, officials were not incurred liabilities for virtually excessive and unreasonable measures, but they would be held accountable where disease circulated: some officials were dismissed because the disease circulated in their territories. ^85^

However, insofar as such expectations are tacit, the central government bear no responsibility in relation to any courses of action of local actors. Meanwhile, certain behaviours of local actors seem to have gone beyond the expectations of the central authorities.

***3.5.2. Local actors***

The behaviours of the central government provided signals for local actors to believe that they were expected to legislate and employ any measures they deemed fit at whatever costs, notwithstanding the central government’s formal and explicit announcements.

It should be noted that there are also reasons for local actors to expect that their measures at whatever costs are valid and beneficial to contribute to the containing of the diseases and that such elimination strategies were bound to be successful. It is helpful to recall that since the beginning of the COVID-19 pandemic, the central government constantly conveyed messages such as “Surely, we will soon defeat the COVID-19 pandemic” and “Rigid and harsh restrictions are the only way to eradicate COVID-19 and to win the battle against COVID-19”. The Vietnamese National Television (VTV), in response to criticisms to reconsider restrictive measures, claimed that *“Living with COVID-19 is the rhetoric of the rebels”*. Thus, there seems to be a reason to believe that officials’ invocation of the protection of public health, and even the health of individuals whose rights were violated, is not necessarily a pretext, but they can also represent staunch expectations. This can be observed on the one hand as to the remarks of various local and central authorities ^18^. A parallel observation can be drawn from a comparative lens. An empirical study by Rachel Wahl suggests that within the context of India and concerning violence of the police, there are moral and social imaginaries that inform and underlie violence. ^86^ Inappropriate measures, abuse, and violations sometimes do not necessarily entail bad faith.

Several factors may aggravate such kind of expectations:

*First,* for so long, officials have been familiar with the idea that claiming rights is selfish and that individuals claiming rights have ill intentions ^87^. It has also been believed that human rights are not just unnecessary and irrelevant to public health and welfare but adversarial to such.

*Second,* as partially mentioned in the previous subsection, actors are not expected to be subject to any constraints. The legal system at wide contains no significant constraints.

(1) Unlike common-law-tradition jurisdictions, e.g., the UK, Australia, New Zealand, there is no principle for the exercise of powers in Vietnam, even such a stringent test like the Wednesbury reasonableness principle ^88^. Nor is there any protection or limitations like the safeguard endowed against the use of unreasonable force ^89^ or endowed to dwellings ^90^. Nor is Vietnam similar to Taiwan where the Administrative Procedure Act provides under Article 7 certain principles in pursuance of which administrative acts shall be performed ^91^. Measures and actions of local actors in such cases, however unreasonable they are, are, valid and lawful to the view of local authorities. This is evident in statements of local agencies and judgments of local courts. However, the very self that previously approved certain conducts may abandon such approval and any responsibilities associated with the approval, arguing that behaviours are not appropriate (and beyond expectations) when facing public backlash ^18^.
(2) The Constitution of Vietnam 2013, while containing various rights promised to be protected, is only on paper and not actionable. That is, persons cannot claim rights protected by the Constitution by resorting to the Constitution and thus Vietnam is, of course, very different to the US where the Constitution is the sacred instrument.
(3) Though Vietnam is a contracting party to various human rights instruments, most notably ICCPR and ICESCR, they are also symbolistic and not actionable. Very rarely did Vietnamese courts enforce international treaties ^92^. Courts have never enforced ICCPR and ICESCR.
(4) Although Article 4 of the 2015 Law on Promulgation of Legislative Documents provides the hierarchy of legal documents and Article 156.(2) of the same law provides that “documents contain different regulations on the same issue, the superior document shall apply”, these rules (as well as the rule concerning the supremacy of the Constitution, the priority of international instruments ^93^) are rarely – if any ever – been complied with. Authorities prioritize and are expected to prioritize subordinate documents (e.g., regulations, directives, circulars) and even obviously non-legal documents (namely, official letters) over superior documents (e.g., the Constitution, primary legislation). The legal system is chaotic.
(5) Vietnam has not signed the First Optional Protocol to the ICCPR for any possible violations to be heard by the mechanisms provided therein and legal and human rights principles to be brought into effect.

*Third,* these expectations can be amplified by biases and heuristics. Such biases and heuristics include

- “Authority bias” (described in the “Obedience to Authority”): it is not likely that local authorities, in the absence of awareness of human rights or of the larger context of the law that pledges to protect rights and dignity, shall self-reflect statements of the central government or their own behaviours.
- Expectations of consensus from the masses: According to the famous *“* False Consensus Experiment”, if people hold certain beliefs or judgments, they may tend to expect that the majority of other people may hold the same beliefs or judgments ^94^. Where news has constantly repeated the statements of the central government, depicted draconian measures as desirable and necessary, and those objecting to these measures as ill-intentioned and even ridiculed such people, then officials have reasons to expect that their actions would be applauded not only by the central government but also by the public and be reasonable. In fact, the official who claimed that bread is not food before being lapidated by the general public had been applauded by the public for uploading videos with similar content.
- “Conformity bias” ^95^: for example, where a group of officials reach a consensus to use force to break into and force testing, or that bread is not food, it is not likely that any individual belonging to the group shall oppose outright that assertion.

These might be aggravated under real-world conditions due to real political hierarchies, authorities, responsibilities, and punishments. It should be noted, strikingly, that the Party employed the principle of “ democratic centralism” developed by Lenin, which aimed to establish blind obedience and iron discipline. Lenin asserted that

“Parties belonging to the Communist International must be organised on the principle of democratic centralism … the Communist parties can perform their duty only if they are organised in a most centralised manner, are marked by an *iron discipline* bordering on military discipline, and have strong and authoritative party centres *invested with wide powers* and enjoying the unanimous confidence of the membership.” (V. I. Lenin, Terms of Admission into Communist International, July, 1920, para. 13) [Emphasis added].

In light of the principle of “ democratic centralism”, decisions of high authority, once made, are expected to be [blindly] followed by all subordinates; that is, there are no expectations that the decisions are to be (re-)discussed or consulted or challenged. Democratic centralism expects officials to submit fully to their superiors, thus aggravating the Obedience to Authority bias. ^96^

*Fourth,* the tangled and wild legal landscape of absence – or, at best, insufficiency – of constraints of powers tacitly permit or even ferment conditions for human beings’ inherent tendency to abuse. It is self-evident that deeply veiled inside selves there are esteem needs that may include needs for status, fame, prestige, and attention ^97^ and inclination to abusing powers, as correctly captured by the Federalist Paper No. 51: “If men were angels, no government would be necessary. If angels were to govern men, neither external nor internal controuls on government would be necessary. In framing a government which is to be administered by men over men, the great difficulty lies in this: You must first enable the government to controul the governed; and in the next place, oblige it to controul itself. A dependence on the people is no doubt the primary controul on the government; but experience has taught mankind the necessity of auxiliary”, Lord Acton well-known aphorism “Power tends to corrupt and absolute power corrupts absolutely”, and empirical evidence in the “ Stanford prison experiment” ^98^. That deep inherent tendency could also be aggravated by the context that may include the social, political, legal structure and might have been observed as particularly relevant and salient within the context of Vietnam. ^99^

Thus, the legal landscape also hints at the normativity of expectations however unreasonable such expectations can be.

***3.5.3. Expectations for the Rule of Law***

It can be argued that the statement of Vũ Công Giao and Đào Trí Úc expressed an expectation for the Rule of Law, the expression of the Rule of Law, and the protection of human rights amidst the Covid-19 pandemic. Such expectations are not exclusively of Vũ Công Giao and Đào Trí Úc but of larger audiences with legal backgrounds who deeply care for democracy and the Rule of Law, as can be observed across jurisdictions and cultures. Though Tom Ginsburg and Mila Versteeg reached an opposite conclusion as to the reality in Vietnam which gave rise to concerns of Vũ Công Giao and Đào Trí Úc, the more overarching thread of their paper, without doubt, also expresses expectation for and of the Rule of Law, its manifestation, and the protection of human rights. It is useful to recall other instances as well. Within the context of the UK, where the delegation of powers and the wide use of secondary instruments and the Henry VIII Clause (i.e., a clause or clauses in a bill that enables ministers to amend or repeal provisions in an Act of Parliament using secondary legislation) have been observed, the UK Parliament has expressed concerns in relation to this practice inasmuch as it has implications for the Rule of Law. Several reports have been produced by the House of Commons Joint Committee on Statutory Instruments (‘JCSI’), the House of Lords Delegated Powers and Regulatory Reform Committee (‘DPRRC’) and the House of Lords Secondary Legislation Scrutiny Committee such as the Rule of Law Themes from the Scrutiny of COVID-19 Regulations First Special Report of Session 2021–22 of JCSI, ‘ Democracy denied? The urgent need to rebalance power between Parliament and the executive’ of DPRRC or Government by diktat: A call to return power to Parliament of SLSC. Paragraphs 2, 3, 5 of the Rule of Law Themes from the Scrutiny of COVID-19 Regulations First Special Report of Session 2021–22 read

“2. It became apparent to the Committee … several common themes were emerging. Some of these themes appeared to raise rule of law issues...
3. The purpose of this Report is therefore to draw … matters which the Committee believes are of general and enduring significance in relation to the preservation and protection of the rule of law…
5. Despite this, the Committee decided early on that the necessary constraints on the normal law-making processes for subordinate legislation during the pandemic made it all the more important for the Committee to continue to play its role in preserving fundamental rule of law standards in relation to delegated legislation.”

Paragraph 5 of ‘Democracy denied? The urgent need to rebalance power between Parliament and the executive’ reads

5. “…We take the view that a critical moment has been reached where action is needed to bring about significant change in the way in which legislation is framed so that it is, first and foremost, founded on the principles of parliamentary democracy, namely parliamentary sovereignty, the rule of law and the accountability of the executive to Parliament”

Paragraph 5 of ‘Government by diktat: A call to return power to Parliament’ reads

“The statement should require ministers, when seeking a delegation of legislative power, to take into account to the fullest extent possible the principles of parliamentary democracy, namely parliamentary sovereignty, the rule of law and the accountability of the executive to Parliament.”

Meanwhile, in the Borrowdale Case, in response to concerns for the Rule of Law of the New Zealand Law Society (Intervener) ^100^, though the measures impugned had ceased to be in effect for which it was argued by the Crown that the questions are academic and thus no declaratory relief should be granted ^101^, the High Court, despite recognizing that the fact that the unlawfulness has long since been remedied militates against making a declaration, granted declaratory relief by virtue of weighty rule of law considerations. It opined that

“Although the state of crisis during those first nine days goes some way to explaining what happened, it is equally so that in times of emergency the courts’ constitutional role in keeping a weather eye on the rule of law assumes particular importance. For these reasons we conclude that it would be appropriate to make a declaration” ^102^.

Notwithstanding, as mentioned in Section 3.1 and Section 3.3, expectations need not be well-justified, realistic, can be daydreaming, and are, to a recognizable extent, shaped and facilitated by the context.

On the one hand, because on April 1, 2020, Prime Minister Nguyễn Xuân Phúc signed Decision 447/QD-TTg recognizing the COVID-19 epidemic as ‘ *class A infectious diseases’* ^103^, the claim that a formal declaration of emergency (which is not precisely emergency powers nor certain de facto constraints and check upon such powers) is so necessary and essential for the Rule of Law and the protection of human rights can be cast doubt upon. It is hard to find what further benefits a [further] formal declaration can add. The argument that a [further] formal declaration of emergency is a necessary condition for emergency powers and human rights derogation is hardly convincing because different to the International Covenant on Civil and Political Rights (ICCPR), the 2013 Constitution does not make a distinction between restricting rights and derogation from rights. Under Article 14 of the 2013 Constitution, all rights (including non-derogable rights as provided for under the ICCPR) can be restricted by primary laws (satisfying Article 4.3 of ICCPR is, however, a different matter because the procedure thereof relates to notification of/from a state to other states) ^104^. As shown in the previous subsection, it cannot be argued that only in light of a formal declaration of emergency would the Vietnamese Government have sufficient powers for the purpose of protecting public health ^105^. Article 53 of the LPCCD is even broader than Article 54 concerning measures to be applied in a state of emergency in case of epidemic. While Article 54 [only] permits “the application of other measures specified in Section 3 of this Chapter” (Article 54.2.(h)), Article 53.(1).(d) of the LPCCD allows any other measures. Notwithstanding, the validity of such legal instruments can hardly be doubted. As Gustav Radbruch put straightforwardly: *“a law is a law which is valid because it is a law, and it is a law if, in the general run of cases, it has the power to prevail”* ^106^, the positive law takes precedence even when its content is unjust and fails to benefit the people ^107^. However broad and susceptible to being violated the LPCCD, its associated subordinated instruments, and measures are, they are valid and have the force of law. This is what has been confirmed by the judgment of a Vietnamese local court. Meanwhile, it should also be noted that not all jurisdictions declared a formal declaration of emergency. Many jurisdictions, such as the UK, did not make a formal declaration of the state of emergency. There can be arguments that the declaration of emergency allowed the emergency powers to be expected to be temporary. But, while it can be argued that the declaration of emergency – in its formalist sense - is not a prerequisite condition for emergency powers to be temporary, that is, without any declaration, measures can still be temporary, it can also be argued that with the declaration, measures are not only legitimatized but also perpetuated ^108^. It should also be noted that authorization and supervision do not necessarily take the form of a formal declaration of emergency made in advance of the measures. ^109^ Notwithstanding, it can be argued that social context gives rise to such kinds of expectations as of Vũ Công Giao and Đào Trí Úc. In addition to the expectations of the Rule of Law and the expression of the Rule of Law, and human rights protection, the doctrine of mingjiao (‘the doctrine of names’) of Confucianism that has profound influence within the context of Vietnam emphasizes the importance of justifying rationale and using proper formal titles.

On the other hand, more strikingly, for many readers, especially foreign scholars, expecting the Rule of Law, the manifestation of the Rule of Law, and human rights protection in a system with disabled checks and constraints like Vietnam is not realistic, naïve, and daydreaming. Clearly, the provisions such as Article 53.1.(d) of LPCCD that permit any other measures deemed fit by authorities without any checks and constraints are alien to developed jurisdictions, may they be in the East or the West. Meanwhile, there is no – or at best, there is insufficiency of - independence between institutions ^110^. Most of the deputies of the National Assembly are members of the Vietnamese Communist Party (only 14 out of around 500 members are non-Party members), similar can be observed with local People’s Councils. Chief Justice of the Supreme People’s Court was given to a member of the Communist Party’s Central Committee, while the Deputy Chief Justice and Judges of the Supreme People’s Courts were nominated by the Party. Likewise, Chief Judges of local People’s Courts are also Members of the local Party Committee. Meanwhile, where a draft bill is not endorsed by the National Assembly, deputies of the National Assembly who are members of the Vietnamese Communist Party shall be ‘quán triệt-ed’ (i.e., to be put to understand thoroughly) in favour of endorsing the not-yet-to-have-been-passed draft bill by a superior Party authority or authorities before it is voted again in a shape that is not so much changed. Judges of lower courts in deciding cases may ask for opinions from their chiefs or superior courts (‘ thỉnh thị án’) and before ‘hard cases’ are adjudged there can be joint meetings between the Judiciary, the Procuracy, the Police, the Party Internal Affairs Committee organized to determine the ‘orientation’ for adjudicating the case ^111^. It can also be argued that social context gives rise to such kinds of expectations concerning the Rule of Law, the expression of the Rule of Law, and human rights protection in Vietnam. Such expectations reflect the process of transition and opening in Vietnam (‘Đổi Mới’). Before Đổi Mới – the period when Vietnam decided to implement liberal economic policy and open to the world – though there had been laws, if not to say a lot of laws (Because Vietnam followed a Soviet Commanding Economy Model, periodically, the Government commanded which goods to produce, which governmental agencies would be involved in the production, what would be the price of input and output goods, and even who would be distributed/sold goods and how much the volume of the distribution/sell would be for each individual), the Rule of Law and human rights were not recognized. The party-state only recognized socialist legality. Law was just a discipline of the Party and an instrument of the state ^112^. Not different to today, any administrative behaviours could be considered laws; laws have been amenable to being changed entirely regularly via administrative processes. There was also no place for “human rights”: Rights were understood as civic rights at best and inseparable from duties: the State only guaranteed civic rights as long as the citizens had fulfilled their duties first ^113^. Socialist rule of law and socialist rule of law-based State was adopted in the Seventh Congress of the Party and Resolution 51/2001/NQ-QH10. Though scholars of the Party and other scholars may continue to dispute concerning what is the substance of the so-called Socialist Rule of Law-based State, both groups recognize the principle of *‘the supremacy of the Constitution’* (though sometimes, a high-ranking Party official may believe that the Constitution is secondary important after the Party’s Platform). The concept of “human rights” has been embraced ultimately in the 2013 Constitution. Consequently, there is a point for hope and daydreaming however frail and not quite realistic. But for that purpose/vision, there are ‘miles to go before I sleep’. ^114^

## 4. Implications for governance and research agenda for the future

### 4.1. Implications for governance

The above sections logically implicate that those social institutions, for example, (bio)ethics and law, whose common aims are to provide guidance for or dictate or regulate behaviours, to assist and guide decision-making concerning particular settings and contexts, and to shape realities, must pay attention to expectations. Ethics and law, conceding or not, have been informed by and have relied on ‘expectations’ though expectations do not affect ethics and law in identical manners. Certain similarities in the manners in which expectations affect ethics and law, however, can be observed.

What is ethical or not is, in certain instances, influenced by and even shaped on and around expectations, e.g., expectations of a society ^115^. Ethics as studies or a practical idea entail expectations for ethical behaviours (otherwise, ethics can be utopian).

Likewise, the law, for it to effectively guide behaviours of selves, must pay attention to selves’ expectations and be responsive to these. The law can regulate society precisely by imagining and expecting various behaviours which can potentially happen and provide dictations, demands, and guidance accordingly. The political community shaping the law (for some reasons) expects certain behaviours as being necessary, beneficial, or at least not immoral and/or harmful, which can be demanded or permitted. It also expects certain behaviours to be harmful or unethical, which must be prohibited, prevented (via certain arrangements), sanctioned, and coerced. It also follows that governments prepare their policy, laws, propositions, and behaviours upon expectations of their populace's attitudes and behaviours, such as expectations of general patterns of behaviours of the populace, expectations of and for social institutions such as ethics, social norms, and peers’ attitude, and expectations of expectations and attitudes of the populace vis-à-vis the planning policy, laws, propositions, and behaviours. The evaluation and planning of policy, laws, propositions, and behaviours take place in a plane in which expectations of likelihoods, outcomes, and capacity, among other factors, matter and are taken into account. It thus follows that initial expectations are measured against de facto patterns of behaviours, likelihoods, outcomes, and capacity, among other factors, which are kept under constant monitoring for the purpose of appropriately adjusting policy, laws, propositions, and behaviours.

The law, of course, expects it to be obeyed by the population over which it rules and regulates. *Yet,* simultaneously, it also expects the occurrence of deviants and arranges specific sanctions and remedies for specific deviants, as well as specific procedural steps for the imposition of sanctions and remedies accordingly. Nevertheless, the entire legal governance structure must be structured in such a manner that can give robust expectations that obedience shall overwhelm deviants, quantitively and qualitatively, for it is evident that the law can effectively guide behaviours of selves (otherwise, if the policy and law are expected not to be expected, they should be revised with a view of being brought into effectiveness, for not, they will certainly not be obeyed). The law can effectively guide behaviours of selves only if it can yield its subject's expectations of it: selves must have expectations of the law and rely on the law in forming expectations and behaving - as opposed to, for example, expectations of force, violence, despot, arbitrariness, and undue influences. However, as can be observed in the Vietnamese cases described, for that effect to be produced, it is not conditional on the existence of the law but only on the existence of the law that has the quality capable of producing that effect (i.e., being expected). That is, the law must have qualities and features capable of being expected and relied on by its subject. The “inner morality of Law” ^116^, i.e., the Rule of Law and such features being principles of the Rule of Law, and the suitability of the law to circumstances which the former is expected to be applied to are among such qualities. ^117^ Meanwhile, on the one hand, even where the law allows discretion on the side of certain actors, for it to still be expected [by the general public], the law must impose upon such actors certain expectations and demand these actors to meet these expectations. These expectations are expressed in, on the one hand, excellences expected to be expressed by actors (particularly actors holding public offices), namely reasonableness, transparency, impartiality and, on the other hand, ethical and professional rules, principles, codes of conduct, and guidance ^118^. On the other hand, the law aims to form legitimate expectations of actors and only legitimate expectations formed in reliance on the law are expected to be honoured and protected by the law. That also means expectations of and for the law have the effect of narrowing down the range of expectations and making life and society more certain. Ultimately, the law is a valuable tool for the management of expectations and for the management of life and society amidst situations of crisis which can give rise to too many expectations.

A corollary of the above is this: A bad law is a law that is capable of being invalidated or incapable of being expected. In situations where the law is incapable of being expected, there shall be too many expectations, especially too many undue expectations, which can have the effect of casting a society profoundly uncertain and fermenting horrendous behaviours.

### 4.2. Agenda for future research

Notwithstanding the point mentioned in the previous section, as mentioned earlier, the concept of expectation has been of use and significance in a wide range of areas, including ethics and law ( Section 3.1), and for that reason, there are points for ethical and legal studies to [continue to] pay attention to the concept of expectations and broaden the scope of its applicability (though I shall not attempt to repeat or recast these points in details here).

Meanwhile, if the analysis provided in the above section holds true, that is, expectations account for human rights violations and related propositions within the context of the Covid-19 pandemic in Vietnam, then it is likely that the line of analysis can be extended to be applied to the Vietnamese society-wide and many – if not any - behaviours thereof. That is to say, *presumably*, expectations are underlying and account for and have implications for the wide Vietnamese societies and any interactions and behaviours thereof. As a result, expectations are not only inevitable but also a valuable and crucial device to understand certain societies such as Vietnam and all interactions and behaviours thereof. If it is true, expectations are underlying and account for and have implications for comparable societies – those societies in which there are rich social interactions, or there are weak governance structures. That, however, does not preclude, dismiss, or imply to preclude or dismiss the relevancy and significance of expectations to and to the studies of other societies. Consequently, expectations have very wide and significant implications for research across areas and countries, e.g., in relation to governance, which, however, this paper admits its failure to identify in detail. Thus, hereunder, it shall only attempt to mention points which are of proximity to this paper's research subject - governance (during crises such as the COVID-19 pandemic, especially within the context of Vietnam).

Insofar as ‘expectations’ is a concept connecting selves and contexts, it stands for the potentiality of contributions from various research disciplines, cooperations between these disciplines, and implications for better governance. This, to a certain extent, is reflected by the literature concerning expectations mentioned in Section 3.1. Here, I shall just go a little bit further. For example, in light of certain psychological traits of actors, certain expectations related to actors’ behaviours can be guesstimated, e.g., the tendency to abuse powers and towards corruption of powers (this is what classical writings are about, see again Federalist Paper No 51). However, these traits are militated in favour or against by and under certain social, political, and legal structures (See Section 3). As a result, guesstimates need to be reflected and tested. That is to ask how is that possibility realised in societies with different cultures and social structures? Which arrangements militate in favour or against the possibility of abuse of powers? Or this paper offers an observatory account that there is a tendency of lower-ranked officials to 'guess' the expectations of their superiors, but is that observation correct, to what extent is it correct, does that hold true in all societies and to what extent it is true across societies? Or how might selves weigh expectations formed on informal institutions and interactions in comparison to expectations formed on formal ones? That signifies the need for more [interdisciplinary and intercultural] studies for a better understanding of the matters and fuller explanatory accounts.

Likewise, the formulation of solutions should necessarily be considered and reflected by the examination of expectations and associated features. Inter- and multi-disciplinary studies are also helpful for that purpose. For example, the expectations of Tom Ginsburg and Mila Versteeg as to the role of local actors in constraining central government might be reasonable within the context of the US, *to an extent* [Emphasis]. In the US, as mentioned, powers are delegated to the central government and the local government in parallel and powers which are not delegated explicitly to the central government stay with the local government or residual with the people. But that does not mean that protection for rights is, *in general*, relegated: the government is chosen on a democratic basis, and the US Constitution is of sacred value, which is safeguarded by multiple checks ^119^. However, when such expectations, i.e., of ‘subnational constraints’, are extrapolated to other contexts, i.e., beyond the boundaries of the US, they may result in overgeneralization and inappropriateness, which is not without repercussions. This can be drawn from not only the Vietnamese cases described but also the Role of local government in the promotion and protection of human rights – Final report of the Human Rights Council Advisory Committee. Paragraph 31 of that report reads, “The biggest challenge facing local governments is lack of political will, particularly in countries with non-democratic systems or with incipient democracies”. Paragraph 34 reads “A further challenge is the lack of information about the requirements resulting from human rights at the local level ... Often this awareness lacks a well-founded knowledge about the content and the scope of human rights. As a result, many local governments fail to understand and incorporate human rights into local policy and practice. Efforts to increase human rights coordination are marred by a lack of transparency”.

Finally, it is obvious from other studies [in other areas] and from the analysis provided by this study that expectations bring about norms and rules, and thus, for social changes, altering and channelling expectations (towards being legitimate and reasonable) should contribute to an extent. In relation to the issue of human rights and governance, that is evident in the advice of the United Nations Human Rights Council Advisory Committee that better understanding and commitment of local actors to human rights may produce a positive impact on human rights protection and democracy ^120^. This, of course, implies that there should be studies as to how to alter and channel expectations in certain [underdemocratic] societies.

## Conclusion

This paper conceptualizes ‘expectations’ for the purpose of bioethical and health law studies. It defines ‘expectations’ as normative imaginaries that can induce or guide actions and inactions. It argues that violations of human rights and certain related propositions within the Vietnamese context can be understood in terms of expectations. Within such contexts as Vietnam, expectations account for behaviours, including human rights violations. Inasmuch as expectations bring about norms and rules, altering and channelling expectations (towards being legitimate and reasonable) should contribute to social changes and improvements. It thus suggests that ‘expectations’ is a fruitful concept for bioethical and health law studies.

## Data Availability

No data are associated with this article.

## Footnotes

^1^ Tom Ginsburg and Mila Versteeg, ‘The Bound Executive: Emergency Powers during the Pandemic’ (2021) 19 International Journal of Constitutional Law 1498, 1534.

^2^ Cass R Sunstein, *This Is Not Normal: The Politics of Everyday Expectations* (Yale University Press 2021) 68–69; Charles de Secondat Montesquieu baron de 1689–1755, *The Spirit of the Laws* (New York: Hafner Pub Co, 1949 1949); Hai Thanh Doan, ‘The Public Health Response to COVID-19 in Vietnam: Decentralization and Human Rights’ (2023) 15 Asian Bioethics Review 103, 114.

^3^ Doan (n 2).

^4^ ibid.

^5^ Yu Insun, ‘Law and Family in Seventeenth and Eighteenth Century Vietnam’ (University of Michigan 1978); Samuel Baron and Hoàng Anh Tuấn, *A Description of The Kingdom of Tonqueen [Mô Tả Vương Quốc Đàng Ngoài]* (Omega Việt Nam 2019); Gustave Dumoutier, *Essais sur les Tonkinois* (Imprimerie d’Extrême-Orient 1908).

^6^ Li Tana and Nguyễn Nghị, *Nguyen Cochinchina: Southern Vietnam in 17th and 18th Centuries [Xứ Đàng Trong: Lịch Sử Kinh Tế Xã Hội Việt Nam Thế Kỷ 17 và 18]* (Trẻ 2013); Pétrus Jean-Baptiste Vĩnh Ký Trương, *Cours d’histoire Annamite à l’usage Des Écoles de La Basse-Cochinchine*, vol 1 (Impr du gouvernement 1875).

^7^ Insun (n 5).

^8^ Hai Thanh Doan, Diep Thi Phuong Doan and Nguyen Kim The Duong, ‘Post-Mortem Reproduction from a Vietnamese Perspective - an Analysis and Commentary’ (2020) 12 Asian Bioethics Review 257; Le Thi Lan, ‘Common Root of Three Religions (三敎同源) in History of Vietnamese Thoughts’ (2016) 36 Studies in Confucianism 535.

^9^ Đoàn Thanh Hải and Đoàn Thị Phương Diệp, ‘What Is Wrong with the Functionalist Approach Used by French Scholars in Studying “Điển”?’ (2023) 11 Comparative Legal History 45.

^10^ Lê Duẩn, *Cách Mạng Xã Hội Chủ Nghĩa Tại Việt Nam, Tập I [Socialist Revolution in Vietnam. Vol I]* (NXB Chính Trị Quốc Gia Sự Thật 1980). Cf writings of Hoàng Quốc Việt (Adam Fforde, ‘The Unimplementability of Policy and the Notion of Law in Vietnamese Communist Thought’ (1986) 14 Asian Journal of Social Science 60, 67.) Socialist rule of law and socialist rule of law-based State was adopted in the Seventh Congress of the Party and Resolution 51/2001/NQ-QH10 (Nghị quyết 51/2001/NQ-QH10 bổ sung điều của Hiến pháp nước cộng hoà xã hội chủ nghĩa Việt Nam năm 1992 [Resolution 51/2001/NQ-QH10 supplementing articles of the 1992 Constitution of the Socialist Republic of Vietnam] 2001. Article 51 The 1992 Constitution 1992; Truong Chinh, ‘Report to the National Assembly 12 December 1980’ (1980) 15 trans. FBIS East Asia Daily Report K15. Pham Duy Nghia and Do Hai Ha, *The Soviet Legacy and Its Impact on Contemporary Vietnam* (2018).

^11^ ‘Đẩy Mạnh Hơn Nữa Việc Phân Cấp, Phân Quyền Trong Quản Lý Nhà Nước [Further Promote Administrative Divisions and Decentralization in State Management]’ ( *Báo Điện tử Chính phủ*, 2021) < https://hcmcpv.org.vn/tin-tuc/day-manh-hon-nua-viec-phan-cap-phan-quyen-trong-quan-ly-nha-nuoc-1491886775> accessed 21 September 2022; Nguyễn Đăng Quế, ‘Một Số Vấn Đề Đặt Ra Trong Phân Cấp, Phân Quyền Giữa Trung Ương và Chính Quyền Địa Phương Hiện Nay [Some Issues Concerning Administrative Divisions and Decentralization between the Central Government and Local Governments Today]’ *Quản lý Nhà nước [State Management Review]* (13 May 2022).

^12^ Ginsburg and Versteeg (n 1) 1534.

^13^ ibid 1528.

^14^ “In Indonesia, several regional governments passed stricter regulations than the national government, prompting the President to issue a statement that “regional heads should not make policies of their own.” And in Colombia, Bogotá Mayor Claudia López resisted President Duque’s decision to allow some construction workers and manufacturers to return to work, prohibiting this in Bogotá, even without the formal authority to do so” ibid.

^15^ The phrase used ‘possible human rights violations’ denotes two important aspects: *First,* to a certain extent, more or less, the finding of human rights violations is subject to the human rights system/the jurisprudence at stake. There can be human rights violations which are universally accepted as such but some violations are violations within a given jurisdiction but not another. This paper does not intend to argue or point out that behaviours mentioned in this paper (and which measures) are human rights violations which are universally accepted although these behaviours appear to be unethical, irrational, and unreasonable. *Second,* there has been not yet any declaration from any competent tribunals, nationally or internationally, confirming that such behaviours are human rights violations. Thus, a disclaimer here is necessary. However, for convenience and brevity, in certain instances, this paper may omit the word ‘possible’.

^16^ Doan (n 2).

^17^ Directive No 16/CT-TTg dated 31 March 2020 which made lockdown possible by prescribing that *“... more than two people should not gather outside offices, schools…”*

^18^ Doan (n 2).

^19^ ibid.

^20^ Cong Giao Vu and Tri Uc Dao, ‘State of Emergency in Vietnamese Law: Reflections on the Government Response to the Covid-19 Pandemic’ (2022) 22 Australian Journal of Asian Law 5.

^21^ ibid.

^22^ Expect, M erriam-W ebster, https://www.merriam-webster.com/dictionary/expect (last visited Jan 11, 2023).

^23^ Some definitions are not relevant to the paper, e.g., to be pregnant.

^24^ Expectation, C ambridge D ictionary, https://dictionary.cambridge.org/dictionary/english/expectation (last visited Jan 11, 2023); Expectation, O xford L earners D ictionaries, https://www.oxfordlearnersdictionaries.com/definition/english/expectation (last visited Jan 11, 2023); Expect, O xford L earners D ictionaries, https://www.oxfordlearnersdictionaries.com/definition/english/expect (last visited Jan 11, 2023); Expect, *supra* note 30; Expect, C ambridge D ictionary, https://dictionary.cambridge.org/dictionary/english/expect (last visited Jan 11, 2023).

^25^ Expectation, *supra* note 32; Expect, *supra* note 32.

^26^ Expect, *supra* note 30.

^27^ *Id.*; Expect, *supra* note 32.

^28^ Expect, *supra* note 30.

^29^ *Id.*

^30^ Expect, *supra* note 32.

^31^ *Id.*

^32^ *Id.*

^33^ *Id.*

^34^ *Id.*

^35^ *Id.*

^36^ *Id.*

^37^ *Id.*

^38^ Expect, *supra* note 30.

^39^ For (1), the fact that one or more people ‘expect changes…’ does not mean there is any rule or obligation dictating as such. For (2), one can daydream and even get fancy results without working hard due to, inter alia, luck, working smartly, making use of the surrounding context and changes. For (3), in the context provided by the sentence, there is a match between the expectations of guests and the real facility of the hotel, but in other instances, there can be mismatches which neither indicate moral nor legal wrong.

^40^ Richard L. Oliver, *Expectancy Theory Predictions of Salesmen’s Performance*, 11 J. M ark. R es. 243, 244–245 (2018).

^41^ M ankiw, P rinciple of E conomics 4 (9th ed. 2021).

^42^ John Maynard Keynes, *The Principle of Effective Demand*, G en. T heory E mployment, I nteres. M oney 21, 22 (2018).

^43^ J ohn M aynard K eynes, T he G eneral T heory of E mployment, I nterest, and M oney 134 (2018).

^44^ ibid at xxxviii, 141.

^45^ “animal spirits … a spontaneous urge to action rather than inaction, and not as the outcome of a weighted average of quantitative benefits multiplied by quantitative probabilities” (ibid, 141).

^46^ Mads Borup et al., *The Sociology of Expectations in Science and Technology*, 18 T echnol. A nal. S trateg. M anag. 285 (2006).

^47^ W.I. T homas & D.S. T homas, T he C hild in A merica: B ehavior P roblems and P rograms 571–572 (1928).

^48^ *Robert K. Merton, The Self-Fulfilling Prophecy, 8 A NTIOCH R EV. 193 (1948).* at 421–422.

^49^ Judee K. Burgoon & Jerold L. Hale, *Nonverbal Expectancy Violations: Model Elaboration and Application to Immediacy Behaviors*, 55 C ommun. M onogr. 58 (1988). *Id.*

^50^ Joseph Berger, Bernard P Cohen & Morris Zelditch, *Status Characteristics and Social Interaction*, 37 A m. S ociol. R ev. 241 (1972). *Id.*

^51^ Berger, Cohen, and Zelditch, *supra* note 59.

^52^ Cecilia L. Ridgeway, *Why Status Matters for Inequality*, 79 A m. S ociol. R ev. 1 (2014).

^53^ M Conner, “Health Behaviors” [2001] International Encyclopedia of the Social & Behavioral Sciences 6506; Clayton Neighbors, Dawn W Foster and Nicole Fossos, “Peer Influences on Addiction” [2013] Principles of Addiction: Comprehensive Addictive Behaviors and Disorders, Volume 1 323.

^54^ Mark L Flear’s paper belongs to a Special Issue of the Journal of Law and the Biosciences which is about “advancing understanding of the way in which imaginaries underpin law’s engagement with biomedical technologies and science” (Mark L Flear and Richard Ashcroft, ‘Law, Biomedical Technoscience, and Imaginaries’ (2021) 8 Journal of Law and the Biosciences 1, 2.). It is introduced as “the first collection of law-led interdisciplinary accounts of imaginaries” ibid. Flear also suggested that the concept of ‘expectations’ has “obvious contemporary importance” for biomedical governance and regulation and legal and regulatory studies; however, “their roles receive little attention within legal and regulatory studies” Mark L Flear, ‘Expectations as Techniques of Legitimation? Imagined Futures through Global Bioethics Standards for Health Research’ (2021) 8 Journal of Law and the Biosciences 1, 2. It can be observed from such assertions that ‘expectations’ is a relatively novel area of research in bioethics and health law.

^55^ Mark L Flear, *Expectations as Techniques of Legitimation? Imagined Futures through Global Bioethics Standards for Health Research*, 8 J. L aw B iosci. 1, 2 (2021).

^56^ *Id.* at 3.

^57^ Mathilda Cohen, *The Rule of Law as the Rule of Reasons*, 96 ARSP A rch. für R echts-und S ozialphilosophie / A rch. P hilos. L aw 1, 12–13 (2010); Norimitsu Onishi, *Japan Learns Dreaded Task of Jury Duty*, T he N ew Y ork T imes, Jul. 16, 2007.

^58^ Flear, *supra* note 62 at 3.

^59^ Lord Templeman’s speech in Preston v IRC [1985] 2 All ER 327 at 341, [1985] AC 835 at 866–867; Nadarajah v Secretary of State for the Home Department [2005] EWCA Civ 1363 at para. 68, C.A. (England & Wales).

^60^ T eerawat W ongkaew, P rotection of L egitimate E xpectations in I nvestment T reaty A rbitration : A T heory of D etrimental R eliance 54–55 (2019).

^61^ Saluka Investments BV v. the Czech Republic (17 March 2006), UNCITRAL, point 304.

^62^ Ioan Micula, Viorel Micula, S.C. European Food S.A, S.C. Starmill S.R.L. and S.C. Multipack S.R.L. v. Romania, ICSID Case No ARB/05/20, Final Award (11 December 2013) para 671; El Paso Energy International Company v. The Argentine Republic, ICSID Case No ARB/03/15, Award (31 October 2011). It is not that expectations are legitimate where circumstances facilitate or allow reasons for certain expectations to be formed, but rather expectations are only legitimate if they have certain intrinsic traits. It does not imply that morality can invalidate expectations or laws in each and every situation, however.

^63^ Stanley Milgram, *Behavioral Study of Obedience*, 67 J. A bnorm. S oc. P sychol. 371 (1963); S tanley M ilgram, O bedience to A uthority: A n E xperimental V iew (1974).

^64^ J ames W aller, B ecoming E vil: H ow O rdinary P eople C ommit G enocide and M ass M urder 111 (Second edition. ed. 2007).

^65^ *See more* S. Alexander Haslam, Stephen D. Reicher & Megan E. Birney, *Nothing by Mere Authority: Evidence That in an Experimental Analogue of the Milgram Paradigm Participants Are Motivated Not by Orders but by Appeals to Science*, 70 J. S oc. I ssues 473 (2014).

^66^ Stanley Milgram, Leonard Bickman & Lawrence Berkowitz, *Note on the Drawing Power of Crowds of Different Size*, 13 J. P ers. S oc. P sychol. 79 (1969).

^67^ In 1955, following the announcement of the success of the polio vaccine trial, more than hundreds of thousands of children in the United States received a polio vaccine manufactured by Cutter Laboratories. Cutter’s vaccine contained live, infectious virus instead of inactivated virus. This caused 40000 cases of polio, leaving 200 children with varying degrees of paralysis and killing (Michael Fitzpatrick, ‘The Cutter Incident: How America’s First Polio Vaccine Led to a Growing Vaccine Crisis’ (2006) 99 Journal of the Royal Society of Medicine 156 </pmc/articles/PMC1383764/> accessed 17 February 2023).

^68^ See for example, *Four Aviation Security Service Employees v Minister of Covid-19 Response* [2022] 2 NZLR 26; [2021] NZHC 3012; BC202163751 and *Yardley v Minister for Workplace Relations and Safety* (2022) 19 NZELR 125; [2022] NZHC 291; BC202260255.

^69^ See similar observation Ronald A Cass, ‘Delegation Reconsidered: A Delegation Doctrine for the Modern Administrative State’ (2017) 40 Harvard Journal of Law & Public Policy 147, 154.

^70^ T he A nti-F ederalist, (Herbert J Storing & Murray Dry eds., 1985).

^71^ National Federation of Independent Business v. Sebelius, 567 U. S. 519, 536 (2012) (opinion of ROBERTS, C. J.). The federal government’s powers, however, are not general but limited and divided. See McCulloch v. Maryland, 4 Wheat. 316, 405 (1819).

^72^ *ELTON MAYO, THE HUMAN PROBLEMS OF AN INDUSTRIAL CIVILIZATION (Compass books ed. ed. 1966)*; J ames M. H enslin, S ociology : A down to E arth A pproach 140 (Adam. Possamai & Alphia. Possamai-Inesedy eds., 1st ed. ed. 2010).

^73^ Nuclear Tests (Australia v. France), Judgrnent, I.C.J. Reports 1974, p. 253.

^74^ Southern Pacific Properties (Middle East) Limited v. Arab Republic of Egypt, ICSID Case No. ARB/84/3, Award and Dissenting Opinion, 20 May 1992, para. 83

^75^ Borrowdale v Director-General of Health [2020] 2 NZLR 927; [2020] NZHC 1379; BC202061323

^76^ Ibid, paras 186–187.

^77^ Ibid, para. 188.

^78^ Ibid, para. 189.

^79^ Ibid, para. 190.

^80^ Ibid, para. 191.

^81^ Ibid, para. 225.

^82^ See Article 4 of the 2015 Law on Promulgation of Legislative Documents.

^83^ Though the list of documents provided by the system of legislative documents (provided under Article 4 of the 2015 Law on Promulgation of Legislative Documents) is enumerated and *ex-facie* exhaustive (which means documents which are not enumerated are either documents whose applicability are in relation to specific instances or are not legal documents at all, in fact, it may be non-exhaustive. Oppositions of the enforcement (by the force of law) of Directive 16 and other related Directives argued that they were not legal documents but could only be conceived as internal guidance, which only allowed the Prime Minister to instruct his subordinates on the operation of public sectors. There can be two kinds of counter-arguments in response. The first kind of argument recognises that Directive 16 and other related Directives were not legal documents but they still had the coercive force of law. This argument, if accurate, is a trample on the 2013 Constitution, every explicit and official promise concerning human rights protection and every idea of the legal system (that “Human rights and citizens’ rights may not be limited unless prescribed by a primary law solely in case of necessity for reasons of national defense, national security, social order and safety, social morality and community well-being” (Article 14 of the 2013 Constitution), that the Constitution is supreme (Articles 4, 156 the 2015 Law on Promulgation of Legislative Documents, Article 6 of the 2016 Law on Treaties, and the LPCCD). The other kind of argument, while recalling Article 53.1.(d) of LPCCD that permits any other measures as prescribed by law, recognizes Directive 16 and any administrative propositions or behaviours as having legal coercive force despite not being enumerated by the 2015 Law on Promulgation of Legislative Documents. An analogy can be drawn in relation to precedent. A precedent is not (nor are the Decisions concerning the publication and announcement of precedents) a legal document listed by Article 4 of the 2015 Law on Promulgation of Legislative Documents. However, precedent can be considered a source of law (making each precedent published and announced as a legal document) in light of Article 6.(2) of the 2015 Civil Code (“2. In cases where it is impossible to apply analogy of law … basic principles of civil law provided for in Article 3 of this Code, precedent, and justice shall apply”.) and Article 1 of Resolution 04/2019/NQ-HDTP on Process for Selecting, Publishing and Applying Precedents of the Council of Justices of The Supreme People’s Court which defines precedent as arguments and rulings in a legally effective judgment or decision which is capable of being studied and applied in later cases. It is clearly strange that a document with legal [coercive] force and general scope of applicability is not a legislative document (Article 8.(2) of Resolution 03/2015/NQ-HDTP on Process for Selecting, Publishing And Adopting Precedents of the Council of Justices of The Supreme People’s Court provides that “the judges and the jurors must study and adopt the precedent”). A rebuttal, however, can be drawn upon the traditional distinction between sources of law: customary rule, written laws, and precedent (See Lê Minh Tâm, ‘Giáo Trình Lý Luận Chung về Nhà Nước và Pháp Luật [Coursebook on General Theory of State and Law]’. However, it is just mistaken to classify Vietnamese precedent as what is understood as precedent in other jurisdictions, both in light of its root, shape, and modus operandi. Vietnamese cases in and of themselves do not have any precedential status; rather, they gain the status of precedent in light of decisions announcing precedents. Precedents are in the shape and with the status of precedent as provided by the decision promulgating the precedent rather than the original cases. Vietnamese precedents do not aim to shape and apply general principles or facilitate reasonings [like precedents in other jurisdictions]; rather, they aim to form very specific commands on the outcome the court must reach in a given specific circumstances. As a result, precedents do not differ from written legal documents substantively, see more Trịnh Thục Hiền, ‘Án Lệ Việt Nam: Một Biến Thể Của Văn Bản Quy Phạm Pháp Luật? [Vietnamese Case Law: A Variation of Legal Documents?]’ (2019) 5 Nhà nước và Pháp luật [Journal of State and Law] 3. An alternative sub-argument is that although Directive 16 and related Directives were not legal documents, they are enforceable because local authorities enforced them via Decisions of the People’s Committees of provinces, districts, and communes which are legal documents. However, it should be noted that while such Decisions are legal documents, it is strange for a legal document to invoke the validity and content of non-legal documents for drawing validity and content. Thus, this sub-argument should better be considered as supplemental to the argument that Directive 16 was a legal document rather than an alternative. Notwithstanding, not all local authorities resorted to decisions but instead to official letters which are clearly not legal documents. These official letters, though, are obviously not legal documents because they are mere correspondences between authorities and between authorities and certain citizens, are not standardized, nor promulgated transparently, they may be of legislative nature and authorities likely to apply these official letters.

^84^ Section 3 Vietnamese National Assembly, *Nghị Quyết Số 30/2021/QH15 [Resolution No. 30/2021/QH15]*, (2021).

^85^ See Doan, *supra* note 2.

^86^ R achel W ahl, J ust V iolence, T orture and H uman R ights in the E yes of the P olice 49 (2017).

^87^ There have been lingering concerns in Vietnam that reactionary factors may invoke human rights for the purposes of peaceful evolution. People resorting to human rights can be labelled as ‘reactionary factors’. Web Portal of Police of Binh Thuan Province, *Chiêu Bài “Dân Chủ, Nhân Quyền” - Mũi Tấn Công Thâm Độc Của Các Thế Lực Thù Địch [ “Democracy, Human Rights” - The Malicious Attack of Hostile Forces]*, (2022), https://congan.binhthuan.gov.vn/chieu-bai-dan-chu-nhan-quyen--mui-tan-cong-tham-doc-cua-cac-the-luc-thu-dich-N1115.aspx (last visited Jan 8, 2023).

^88^ Associated Provincial Picture Houses Ltd v Wednesbury Corporation, [1947] 2 All ER 680: A reasoning or decision is unreasonable according to the Wednesbury case if it is so unreasonable that no reasonable person acting reasonably could have made it (p.684).

^89^ See, for example, Sections 92Y, 92ZV of the New Zealand Health Act 1956 No 65.

^90^ See, for example, Section 61 of the UK Public Health (Control of Disease) Act 1984; Section 70 of the New Zealand Health Act 1956 No 65.

^91^ “(1) the method adopted must be helpful to the achievement of the objectives thereof, (2) where there are several alternative methods which will lead to the same result in achievement of the objectives, the one with the least harm to the rights and interest of the people shall be adopted; and (3) the harm that may be caused by the method to be adopted shall not be clearly out of balance against the interest of the objectives anticipated to be achieved”

^92^ The United Nations Convention on Contracts for the International Sale of Goods (CISG) is the only exceptional instance, See, Company W [...] v. S [...] Production and Trading Co., Ltd. Tòa án Nhân dân Tỉnh Bình Dương (Binh Duong Province People Court) Vietnam, 16 March 2022 – 02/2022/KDTM-ST. But that instance is also exceptional.

^93^ Article 6 of the 2016 Law on Treaties concerning “Treaties and provisions of domestic laws” and previously Article 6 of the 2005 Law on the Conclusion, Accession to And Implementation of Treaties consistently provide that “ *In cases where a legal document and a treaty to which the Socialist Republic of Vietnam is a party, contains different provisions on the same matter, the provisions of the treaty shall prevail, except the Constitution*” (this is also reflected in Article 156.(5) of the 2015 Law on Promulgation of Legislative Documents)

^94^ Lee Ross, David Greene & Pamela House, *The “False Consensus Effect”: An Egocentric Bias in Social Perception and Attribution Processes*, 13 J. E xp. S oc. P sychol. 279 (1977).

^95^ Solomon Asch showed that a large majority of participants conformed to the group at least once though such an answer was incorrect. Solomon E Asch, *Studies of Independence and Conformity: I. A Minority of One against a Unanimous Majority.*, 70 P sychol. M onogr. G en. A pplied. 1 (1956).

^96^ See Doan, *supra* note 2. Until recently, a handful of exceptions are developed by the Vietnamese Communist Party. See for example, Conclusion No. 21-KL/TW dated October 25, 2021 of the Fourth Conference of the 13th Party Central Committee; Decree No. 73/2023/ND-CP dated September 29, 2023 Regulations on Encouraging and Protecting Officials Who are Dynamic, Creative, Dare to Think, Dare to Do, Dare to Take Responsibility for the Common Good.

^97^ AH Maslow, ‘A Theory of Human Motivation.’ (1943) 50 Psychological Review 370.

^98^ The *“* Stanford prison experiment *”* suggests that people in positions of power might be prone to unethical behaviour and show less empathy and concern for the welfare of others. P hilip G. Z imbardo, T he L ucifer E ffect: U nderstanding H ow G ood P eople T urn E vil (1st ed. ed. 2007).

^99^ In Vietnamese society, there has been a shared imaginary of powerful and vice officials (mandarins) who could abusively manifest their powers to bend the law for their gains and oppress citizens. Before the pandemic, low-level local authorities had already abused their powers in bureaucratic works. See, for example, Tu Phuong Nguyen, *Flexible and Compassionate, or Violent and Intimidating?: Various Accounts of Law in Vietnam*, 54 C rit. A sian S tud. 67 (2022).

^100^ Borrowdale at para. 287.

^101^ Borrowdale at para. 284.

^102^ Borrowdale at para. 291.

^103^ Under Article 3.1 of LPCDD class A consists of extremely dangerous infectious diseases that can transmit very rapidly and spread widely with high mortality rates or with unknown agents

^104^ To satisfy Article 4.3 of ICCPR, a state - Vietnam - must inform the other States Parties through the intermediary of the Secretary-General of the United Nations of the list of the provisions from which it has derogated or wishes to derogate, accompanied by reasons.

^105^ See this argument in Bùi Thu Hằng, ‘Ban Hành Văn Bản Trong Tình Trạng Khẩn Cấp Nhìn Từ Dịch Bệnh Covid- 19 [Issuing Documents in a State of Emergency Observed from the Covid-19 Pandemic]’ (2020) 408 Nghiên cứu Lập pháp [Legislative Studies]; Cao Vũ Minh, ‘Pháp Luật về Tình Trạng Khẩn Cấp Của Liên Bang Nga và Những Gợi Mở Cho Việt Nam [Law on State of Emergency of the Russian Federation and Suggestions for Vietnam]’ (2021) 434 Nghiên cứu Lập pháp [Legislative Studies].

^106^ Gustav Radbruch, ‘Five Minutes of Legal Philosophy (1945)’ (2006) 26 Oxford Journal of Legal Studies 13, 13.

^107^ Gustav Radbruch, ‘Statutory Lawlessness and Supra-Statutory Law (1946)’ (2006) 26 Oxford Journal of Legal Studies 1, 7.

^108^ C arl S chmitt, P olitical T heology: F our C hapters on the C oncept of S overeignty (George. Schwab & Tracy B. Strong eds., University ed. 2005); Giorgio Agamben, *The State of Exception Provoked by an Unmotivated Emergency*, praxis, Feb. 26, 2020; R obert O. P axton, T he A natomy of F ascism (1st ed. ed. 2004).

^109^ John Ferejohn & Pasquale Pasquino, *The Law of the Exception: A Typology of Emergency Powers*, 216–217; Ginsburg and Versteeg, *supra* note 1 at 1512.

^110^ Concerning the judiciary, see *Id.* at 12–13; Pip Nicholson, *Renovating Courts: The Role of Courts in Contemporary Vietnam*, *in* A sian C ourts in C ontext 528 (2014); Trần Ngọc Đường, *‘Hiến Pháp Năm 2013 và Cơ Chế Kiểm Soát Quyền Lực Nhà Nước, Cơ Chế Bảo vệ Hiến Pháp’’’ [The 2013 Constitution and the Mechanism to Control State Powers and to Protect the Constitution]’*, *in* C Ơ chế Bảo hiến và quyền con ngƯời: kinh nghiệm C ộng hòa L iên bang Đứ c và V iệT N am [M echanism to protect the constitution and human rights: experiences of germany and V ietnam] (Cao Đức Thái ed., 2015).

^111^ See further, for example, Pip Nicholson, ‘Judicial Independence and the Rule of Law: The Vietnam Court Experience’ (2001) 3 Australian Journal of Asian Law 38, 41; Phạm Hồng Thái, Vũ Công Giao and Nguyễn Anh Đức, ‘The Court System in the Fight against Corruption in Vietnam: Traditional Problems and New Challenges from Free Trade Agreements’ (2020) 15 Journal of Vietnamese Studies 77, 88.

^112^ D uẩn, *supra* note 10. Cf writings of Hoàng Quốc Việt (Fforde, *supra* note 10 at 67.).

^113^ Article 51 of the 1992 Constitution 1992 Vietnamese National Assembly, supra note 10; Chinh, supra note 10.

^114^Li-ann Thio, ‘Implementing Human Rights in ASEAN Countries: “Promises to Keep and Miles to Go before I Sleep”’ (1999) 2 Yale Human Rights and Development Journal 1

^115^ See Sections 3.1 and 3.3.

^116^ Lon Luvois Fuller, *The Morality of Law* (Revised ed, Yale University Press 1977).

^117^ These principles include, inter alia, laws be general, public, prospective, coherent, clear, stable, practicable, and respecting human rights. See ibid; TH (Thomas Henry) Bingham, *The Rule of Law* (Allen Lane 2010).

^118^ See, for example, Taiwanese Supreme Court, Constitution Interpretation No. 432 dated 1997/07/11; F E Jackson & Co Ltd v Collector of Customs [1939] NZLR 682 (SC); Wednesbury Case; R (on the application of Gardner and another) v Secretary of State for Health and Social Care and others, [2022] EWHC 967 (Admin); [2022] 4 All ER 896; [2022] PTSR 1338; 185 BMLR 30.

^119^ However, this paper is not without attention to or denies many forgettable mistakes in the US Legal History, e.g., Scott v. Sandford, 60 U.S. 393, 15 L. Ed. 691, 1856 U.S. LEXIS 472, 19 HOW 393 (U.S. March 5, 1857); Korematsu v. United States, 323 U.S. 214, 65 S. Ct. 193, 89 L. Ed. 194, 1944 U.S. LEXIS 1341 (U.S. December 18, 1944). These instances illustrate that checks can be wrong sometimes if and where proper balance and considerations are not given due regard to. These instances should not be read as the futility of a proper system of administration of justice or that justice promises are nothing but a lie, however,

^120^ UN. Human Rights Council. Advisory Committee, ‘Role of Local Government in the Promotion and Protection of Human Rights: Final Report of the Human Rights Advisory Committee’ (Geneva, 7 August 2015) para 28.
