# Supplementary material for: Expectations and Vietnam’s responses during COVID-19: potential human rights violations and related propositions
Source: Wellcome Open Res. 2025 May 13;8:147. Originally published 2023 Mar 31. [Version 4] doi: 10.12688/wellcomeopenres.18972.4 (PMC12121475; doi:10.12688/wellcomeopenres.18972.4)
Supplement: Peer review history

# Reviewer report: Reviewer response for version 4

**DOI:** 10.21956/wellcomeopenres.26602.r123682

**Published:** 2025-06-03

**Authors:** Mohammed R.M. Elshobake

**Affiliations:** 1 International Islamic University Malaysia, Kuala Lumpur, Federal Territory of Kuala Lumpur, Malaysia

The paper presents a conceptual framework by introducing the idea of "expectations" as a lens for analysing governmental behavior during the COVID-19 pandemic in Vietnam. The study is particularly significant as it sheds light on the underexplored legal and ethical implications of decentralised public health responses, especially in authoritarian or transitional systems. By illustrating how implicit and conflicting expectations among central and local authorities contributed to potential human rights violations, the paper offers an good contribution to the fields of bioethics and health law.

 Here are some comments to improve the paper:

1. **In terms of the title:**

If the human rights violations are confirmed through Court rulings or Official legal reports by recognised bodies (e.g., United Nations, Human Rights Watch, national human rights institutions). Then it is strongly recommended to remove the word "potential" from the title. The term "potential" implies uncertainty or speculation, which can weaken the force and clarity of the paper’s legal and ethical conclusions. Therefore, I suggest the following title:

- **"Expectations and Vietnam’s Responses During COVID-19: Human Rights Violations and Legal Propositions".**

I also suggest that the author provide concrete legal propositions or recommendations to address the research problems identified in the study.

1. **In terms of Literature Review:**

One major shortcoming of the paper is the insufficient engagement with previous literature on the topic, particularly about the Vietnamese context or comparable jurisdictions.

 The author mentions only one relevant study—"The Bound Executive: Emergency Powers during the Pandemic” by Tom Ginsburg and Mila Versteeg—which primarily focuses on central government overreach and the role of subnational actors. While the author critiques the assumptions in that paper and uses it to frame some of the conceptual discussion, this alone is not sufficient to establish a clear scholarly context.

 The paper fails to comprehensively review existing academic work on:

- Human rights violations during COVID-19.
- Legal responses to public health emergencies.
- The role of expectations or normative imaginaries in legal theory or governance.
- The behavior of local versus central authorities in emergency situations.

Furthermore, the author does not clearly articulate how this research differs from or builds upon previous studies, nor is the research gap (or contribution to knowledge) explicitly defined. Without a thorough literature review, it is difficult to assess the novelty and scholarly value of the paper. To strengthen the study, the author should review and synthesize a broader range of relevant literature and explicitly state how this work fills a gap or offers a new perspective.

1. **In terms of the Problem Statement:**

It is clear that the research problem identified by the author concerns the broad powers exercised by local authorities in responding to the COVID-19 pandemic, along with the general expectation that these actors are capable of tailoring policies to local needs, conditions, and preferences. However, I believe this issue was not adequately explored in the study. The legal context surrounding the delegation and exercise of such powers was not examined in sufficient depth, as I will elaborate later, and the political, social, economic, and administrative dimensions of the problem were also largely overlooked. A more comprehensive contextual analysis would have significantly enhanced the depth and impact of the research.

1. **Other comments on the content:**

- The discussion of human rights violations in Vietnam was limited and lacked reference to documented human rights reports or pertinent court rulings.
- The research did not include a detailed comparative analysis of contexts similar to Vietnam’s, nor did it examine how these contexts positively addressed human rights issues during the COVID-19 crisis, which could have offered valuable insights for Vietnam’s situation.
- When the author discussed the governance aspect and linked it to the research topic, the principles and standards of governance were not discussed in detail.
- The legal analysis within the research is insufficient and lacks rigor, as it relies on assertions unsupported by clear evidence or detailed justification. For instance, the researcher claims that the 2013 Vietnamese Constitution is merely symbolic and unenforceable, without providing a valid basis for this assertion (Where is the reference for that?).

Legally, the constitution is recognised as the supreme legal document in the hierarchy of laws and cannot be disregarded under any circumstances.

- The author mentioned that although Vietnam is a party to several international human rights instruments—most notably the International Covenant on Civil and Political Rights (ICCPR) and the International Covenant on Economic, Social and Cultural Rights (ICESCR)—these treaties are considered symbolic and unenforceable. Furthermore, it was claimed that Vietnamese courts have never applied the ICCPR or ICESCR in their rulings. However, the researcher did not provide any reliable references to support these assertions.

Additionally, the study failed to address the binding nature of international treaties on their parties, as established under international law. According to Article 26 of the Vienna Convention on the Law of Treaties (1969): *"Every treaty in force is binding upon the parties to it and must be performed by them in good faith (pacta sunt servanda)."*

 This principle clearly establishes that once a state ratifies a treaty, it is legally obligated to implement its provisions in good faith. Therefore, Vietnam, as a signatory to the ICCPR and ICESCR, is bound under international law to uphold the rights enshrined in these covenants.

- The author claims that Vietnam's legal system is chaotic, asserting that the principles of constitutional supremacy and the hierarchy of legal norms are not observed. It is further alleged that subordinate legal documents in Vietnam are often treated as prevailing over higher legal instruments, including the Constitution and fundamental legislation. However, these claims are made without citation of any credible or authoritative sources. Indeed, calling the legal system “chaotic" is not academically rigorous unless supported by credible legal studies, empirical evidence, or assessments from reputable organizations like the World Bank or UN bodies.
- In the conclusion, the author did not provide any practical recommendations to improve the situation in Vietnam in light of the concept of expectations.

 

1. **Recommendations for Improvement:**

- Highlight the theoretical contribution more explicitly—A dedicated section summarizing the conceptual implications for law and governance would enhance clarity.
- Critically engage more directly with the notion of abuse of power—While the paper discusses possible rights violations, stronger language or legal critique would help address the normative stakes.
- Consider including a brief timeline of the main governmental responses to COVID-19 in Vietnam to help readers unfamiliar with the local context.

The paper presents a conceptual framework by introducing the idea of "expectations" as a lens for analysing governmental behavior during the COVID-19 pandemic in Vietnam. The study is particularly significant as it sheds light on the underexplored legal and ethical implications of decentralised public health responses, especially in authoritarian or transitional systems. By illustrating how implicit and conflicting expectations among central and local authorities contributed to potential human rights violations, the paper offers an good contribution to the fields of bioethics and health law.

 Here are some comments to improve the paper:

1. **In terms of the title:**

If the human rights violations are confirmed through Court rulings or Official legal reports by recognised bodies (e.g., United Nations, Human Rights Watch, national human rights institutions). Then it is strongly recommended to remove the word "potential" from the title. The term "potential" implies uncertainty or speculation, which can weaken the force and clarity of the paper’s legal and ethical conclusions. Therefore, I suggest the following title:

- **"Expectations and Vietnam’s Responses During COVID-19: Human Rights Violations and Legal Propositions".**

I also suggest that the author provide concrete legal propositions or recommendations to address the research problems identified in the study.

1. **In terms of Literature Review:**

One major shortcoming of the paper is the insufficient engagement with previous literature on the topic, particularly about the Vietnamese context or comparable jurisdictions.

 The author mentions only one relevant study—"The Bound Executive: Emergency Powers during the Pandemic” by Tom Ginsburg and Mila Versteeg—which primarily focuses on central government overreach and the role of subnational actors. While the author critiques the assumptions in that paper and uses it to frame some of the conceptual discussion, this alone is not sufficient to establish a clear scholarly context.

 The paper fails to comprehensively review existing academic work on:

- Human rights violations during COVID-19.
- Legal responses to public health emergencies.
- The role of expectations or normative imaginaries in legal theory or governance.
- The behavior of local versus central authorities in emergency situations.

Furthermore, the author does not clearly articulate how this research differs from or builds upon previous studies, nor is the research gap (or contribution to knowledge) explicitly defined. Without a thorough literature review, it is difficult to assess the novelty and scholarly value of the paper. To strengthen the study, the author should review and synthesize a broader range of relevant literature and explicitly state how this work fills a gap or offers a new perspective.

1. **In terms of the Problem Statement:**

It is clear that the research problem identified by the author concerns the broad powers exercised by local authorities in responding to the COVID-19 pandemic, along with the general expectation that these actors are capable of tailoring policies to local needs, conditions, and preferences. However, I believe this issue was not adequately explored in the study. The legal context surrounding the delegation and exercise of such powers was not examined in sufficient depth, as I will elaborate later, and the political, social, economic, and administrative dimensions of the problem were also largely overlooked. A more comprehensive contextual analysis would have significantly enhanced the depth and impact of the research.

1. **Other comments on the content:**

- The discussion of human rights violations in Vietnam was limited and lacked reference to documented human rights reports or pertinent court rulings.
- The research did not include a detailed comparative analysis of contexts similar to Vietnam’s, nor did it examine how these contexts positively addressed human rights issues during the COVID-19 crisis, which could have offered valuable insights for Vietnam’s situation.
- When the author discussed the governance aspect and linked it to the research topic, the principles and standards of governance were not discussed in detail.
- The legal analysis within the research is insufficient and lacks rigor, as it relies on assertions unsupported by clear evidence or detailed justification. For instance, the researcher claims that the 2013 Vietnamese Constitution is merely symbolic and unenforceable, without providing a valid basis for this assertion (Where is the reference for that?).

Legally, the constitution is recognised as the supreme legal document in the hierarchy of laws and cannot be disregarded under any circumstances.

- The author mentioned that although Vietnam is a party to several international human rights instruments—most notably the International Covenant on Civil and Political Rights (ICCPR) and the International Covenant on Economic, Social and Cultural Rights (ICESCR)—these treaties are considered symbolic and unenforceable. Furthermore, it was claimed that Vietnamese courts have never applied the ICCPR or ICESCR in their rulings. However, the researcher did not provide any reliable references to support these assertions.

Additionally, the study failed to address the binding nature of international treaties on their parties, as established under international law. According to Article 26 of the Vienna Convention on the Law of Treaties (1969): *"Every treaty in force is binding upon the parties to it and must be performed by them in good faith (pacta sunt servanda)."*

 This principle clearly establishes that once a state ratifies a treaty, it is legally obligated to implement its provisions in good faith. Therefore, Vietnam, as a signatory to the ICCPR and ICESCR, is bound under international law to uphold the rights enshrined in these covenants.

- The author claims that Vietnam's legal system is chaotic, asserting that the principles of constitutional supremacy and the hierarchy of legal norms are not observed. It is further alleged that subordinate legal documents in Vietnam are often treated as prevailing over higher legal instruments, including the Constitution and fundamental legislation. However, these claims are made without citation of any credible or authoritative sources. Indeed, calling the legal system “chaotic" is not academically rigorous unless supported by credible legal studies, empirical evidence, or assessments from reputable organizations like the World Bank or UN bodies.
- In the conclusion, the author did not provide any practical recommendations to improve the situation in Vietnam in light of the concept of expectations.

 

1. **Recommendations for Improvement:**

- Highlight the theoretical contribution more explicitly—A dedicated section summarizing the conceptual implications for law and governance would enhance clarity.
- Critically engage more directly with the notion of abuse of power—While the paper discusses possible rights violations, stronger language or legal critique would help address the normative stakes.
- Consider including a brief timeline of the main governmental responses to COVID-19 in Vietnam to help readers unfamiliar with the local context.

**In conclusion, the paper may be considered suitable for indexing, provided that the aforementioned issues are thoroughly addressed.**

Is the work clearly and accurately presented and does it cite the current literature?

Partly

If applicable, is the statistical analysis and its interpretation appropriate?

Not applicable

Are all the source data underlying the results available to ensure full reproducibility?

Partly

Is the study design appropriate and is the work technically sound?

Yes

Are the conclusions drawn adequately supported by the results?

Partly

Are sufficient details of methods and analysis provided to allow replication by others?

Yes

Reviewer Expertise:

LAW

I confirm that I have read this submission and believe that I have an appropriate level of expertise to confirm that it is of an acceptable scientific standard, however I have significant reservations, as outlined above.

# Reviewer report: Reviewer response for version 4

**DOI:** 10.21956/wellcomeopenres.26602.r123470

**Published:** 2025-05-28

**Authors:** Rodrigo Lopez-Barreda

**Affiliations:** 1 School of Medicine, Pontificia Universidad Catolica de Chile, Región Metropolitana, Chile

Thank to the author for this interesting article, which reflects many of the observations made earlier. In this version, the central argument, as well as its potential implications for ethics and law, are much clearer.

 I believe this article contributes by presenting expectations as a relevant element when conducting ethical analyses, making it easier to engage in various debates. For example, as the author points out, expectations may not be entirely rational, so it is necessary to establish to what extent the government should respect less rational expectations in the context of public emergencies, such as the COVID-19 pandemic. Expecting to be unaffected by a pandemic and maintain a completely normal life is, from a medical point of view, an unrealistic expectation, but many people expected that from governments: complete protection without interference in their daily lives.

 Indeed, as the author comments, a lower level of respect for the rule of law is expected from non-democratic governments, but at the same time there is concern about what happens in more democratic states, but where citizens do not show civic and responsible behavior.

 I think that thanks to this article, interesting lines of debate are opened, in preparation for possible future similar events.

Is the work clearly and accurately presented and does it cite the current literature?

Yes

If applicable, is the statistical analysis and its interpretation appropriate?

Not applicable

Are all the source data underlying the results available to ensure full reproducibility?

No source data required

Is the study design appropriate and is the work technically sound?

Partly

Are the conclusions drawn adequately supported by the results?

Partly

Are sufficient details of methods and analysis provided to allow replication by others?

Yes

Reviewer Expertise:

Public health ethics, global health, humanitarian contexts

I confirm that I have read this submission and believe that I have an appropriate level of expertise to confirm that it is of an acceptable scientific standard.

# Reviewer report: Reviewer response for version 3

**DOI:** 10.21956/wellcomeopenres.24642.r121705

**Published:** 2025-04-09

**Authors:** Rodrigo Lopez-Barreda

**Affiliations:** 1 School of Medicine, Pontificia Universidad Catolica de Chile, Región Metropolitana, Chile

Thank you very much for the opportunity to read this interesting article, which addresses such a relevant topic as expectations. Despite being an important part of the decision-making process, they are often overlooked and not considered in our ethical and legal analysis. This reflection is key to a better understanding of human nature, which is not exclusively rational, but also has a significant emotional component. However, the article seems (overly) ambitious and purports to offer an exhaustive reflection on concepts with a rich meaning and multiple interpretations. Therefore, although the main point seems well-defined, the thread of some sections is not sufficiently clear.

 Expectations can be approached from different perspectives. Agents have expectations about their environment, but they can also perceive (or interpret) the expectations that other people may have of them (social expectations). The theory of planned behavior, for example, presents a clear distinction between these two aspects, as the analysis is quite different—in fact, almost opposite: internal expectations can increase agency, while external expectations can decrease it. It's worth noting that, according to this theory, expectations have a direction (positive or negative, according to the value that the agent assigns to the outcome), but the probability of the outcome also plays an important role. Even if the author chooses not to use this theoretical framework, greater clarity about the directionality of expectations would make the argument clearer.

 This distinction could help us analyze the expectations people have of the government, but also those the government might have of the population. In the case of a pandemic, for example, the government might not impose strict regulations, hoping that the population will adopt minimal precautionary measures or that diseases will not spread significantly; but if these expectations are not met, stricter measures might be necessary. In Chile, for example, when the pandemic hit, the country was emerging from a deep political crisis in which civil society expressed numerous social demands. In a context where public discourse spoke of solidarity with the most vulnerable, the government expected for high voluntary vaccination rates... which ultimately didn't happen, and coercive measures were implemented.

 Another clarification that should be made is that expectations affect ethics and law differently, as they are very different in nature: ethics is intrinsically a philosophical discipline, while law includes this moral reflection and also a political process. While it is true that laws are assumed to reflect moral values, the truth is that this is not always the case. In the same vein, there are different branches of law, and expectations can play a different role in the respective legal debates. Expectations can play a very important role in civil and international law, but probably less so in criminal law (at least in countries with positive code systems; in contexts where common law applies, expectations could play a more important role).

Is the work clearly and accurately presented and does it cite the current literature?

Yes

If applicable, is the statistical analysis and its interpretation appropriate?

Not applicable

Are all the source data underlying the results available to ensure full reproducibility?

No source data required

Is the study design appropriate and is the work technically sound?

Partly

Are the conclusions drawn adequately supported by the results?

Partly

Are sufficient details of methods and analysis provided to allow replication by others?

Yes

Reviewer Expertise:

Public health ethics, global health, humanitarian contexts

I confirm that I have read this submission and believe that I have an appropriate level of expertise to confirm that it is of an acceptable scientific standard, however I have significant reservations, as outlined above.

# Response

**Published:** 2025-04-10

**Authors:** Hai Doan

**Affiliations:** University of Otago, New Zealand

Thank you very much for your kind and generous reading and helpful comments. I hope that my clarification in this response together with some minor additional details for the purpose of clarification helps address your concerns,

 Expectations can be approached from different perspectives.  >> This is true. Different approaches to expectations are mentioned in Section 3.1. Agents have expectations about their environment, but they can also perceive (or interpret) the expectations that other people may have of them (social expectations). >> This is true and mentioned in Section 3.2. This point is also elaborated subsequently. Yet, you provide a concise statement or description of this matter, and I am appreciative of your helpful recommendation here. Section 3.2. revised reads “Several objects can give rise to expectations: persons (capturing both individuals and entities, the plurality includes the singularity), institutions (formal and informal), and manifestations (real, potential, or unreal, events included). Institutions are distinct and independent from persons (who invent and operate the institutions), and manifestations are mentioned in the sense emphasizing the manifestations themselves as opposed to the agency that performs or induces such manifestations. Meanwhile, only persons and institutions (generally called selves) are capable of holding expectations. It can be put differently that agents are capable of expectations which are directed toward their environment which is a subset of surrounding subjects and objects. Conversely, they also receive, perceive, or interpret expectations of other agents.”   The theory of planned behavior, for example, presents a clear distinction between these two aspects, as the analysis is quite different—in fact, almost opposite: internal expectations can increase agency, while external expectations can decrease it. It's worth noting that, according to this theory, expectations have a direction (positive or negative, according to the value that the agent assigns to the outcome), but the probability of the outcome also plays an important role. Even if the author chooses not to use this theoretical framework, greater clarity about the directionality of expectations would make the argument clearer.   >>>> >>> I agree with the “Planned behavior theory”. I am thankful for your comment, which helped me to become aware of the theory of planned behaviour. This is what is really helpful and desirable for the reviewing process. However, on the one hand, I am not ready to endorse that “internal expectations can increase agency, while external expectations can decrease it”. To endorse this requires analysis. However, while the increase or decrease of agency is not the thread that this topic is dedicated to addressing, there can be examples that external expectations help increase agency and ability, e.g., can be observed in the Thomas theorem and related theories and concepts or ‘expectancy theory of motivation’.  I also do not observe any “opposite” or “contradiction” between the “theory of planned behavior” and other theories and my analysis. I add the “Planned behavior theory” in Section 3.1 as follows “Certain aspects of each of the above theories are captured by ‘the Theory of Planned Behavior’. Accordingly, the interaction between behavioural beliefs, which are yielded by the expectations of outcomes, with control beliefs, which are the expectations of the ability to perform behaviours and normative beliefs, which are expectations of attitudes of peers and respected figures toward the behaviours, forms the precise intention and behaviours ^53^ .” The paper, as it stands, has already captured that point (expectations include the assessment of possibility/probability and capacity, they reinforce or discourage behaviours). However, I think the paper is still clear without precise words like “negative” or “positive”, which can be abstract or vague and need further elaborations and explanations.     

 This distinction could help us analyze the expectations people have of the government, but also those the government might have of the population. In the case of a pandemic, for example, the government might not impose strict regulations, hoping that the population will adopt minimal precautionary measures or that diseases will not spread significantly; but if these expectations are not met, stricter measures might be necessary. In Chile, for example, when the pandemic hit, the country was emerging from a deep political crisis in which civil society expressed numerous social demands. In a context where public discourse spoke of solidarity with the most vulnerable, the government expected for high voluntary vaccination rates... which ultimately didn't happen, and coercive measures were implemented. >>> I appreciate your perspective and the Chile experience. In fact, that is expressed via these statements already made by the paper “Likewise, the law, for it to effectively guide behaviours of selves, must pay attention to selves’ expectations and be responsive to these. The law can regulate society precisely by imagining and expecting various behaviours which can potentially happen and provide dictations, demands, and guidance accordingly”. However, I think further elaborations help clarify the matter. Thus, I added “It also follows that governments prepare their policy, laws, propositions, and behaviours upon expectations of their populace's attitudes and behaviours, such as expectations of general patterns of behaviours of the populace, expectations of and for social institutions such as ethics, social norms, and peers’ attitude, and expectations of expectations and attitudes of the populace vis-à-vis the planning policy, laws, propositions, and behaviours. The evaluation and planning of policy, laws, propositions, and behaviours take place in a plane in which expectations of likelihoods, outcomes, and capacity, among other factors, matter and are taken into account. It thus follows that initial expectations are measured against de facto patterns of behaviours, likelihoods, outcomes, and capacity, among other factors, which are kept under constant monitoring for the purpose of appropriately adjusting policy, laws, propositions, and behaviours.”

 Another clarification that should be made is that expectations affect ethics and law differently, as they are very different in nature: ethics is intrinsically a philosophical discipline, while law includes this moral reflection and also a political process. >>> The paper have already acknowledged that ethics and law are different animals: “For clarity, this paper deploys phrases ethics and law and/or rights together to signify that they are not synonymous but should be simultaneously considered in relation to a particular issue.” >> The analysis of Section 4.1. also indicates that expectations affect ethics and law in not identical manners. However, for clarity, I added “Ethics and law, conceding or not, have been informed by and have relied on ‘expectations’ though expectations do not affect ethics and law in identical manners. Certain similarities in the manners in which expectations affect ethics and law, however, can be observed.” While it is true that laws are assumed to reflect moral values, the truth is that this is not always the case. In the same vein, there are different branches of law, and expectations can play a different role in the respective legal debates. Expectations can play a very important role in civil and international law, but probably less so in criminal law (at least in countries with positive code systems; in contexts where common law applies, expectations could play a more important role). >> I read your statement that you agree with me that expectations still matter in, e.g., criminal law. It is just about the precise extent of influence of expectations. It may be true or not that expectations matter more in civil law or international law (compared to criminal law) where intentions and interactions of parties are driving factors. However, that does not affect my analysis of expectations of the law. And even in countries where codes are the primary source of [criminal] law, codes can be abstract and determining whether or not certain crimes have been constituted relies on expectations of what prudent or proper people should do in the precise context. Again, it does not change the nature of the analysis. However, this level of detailed explanation is not necessarily included in the main text as it is a little far from the thread (i.e., the distinction between branches of law).

# Reviewer report: Reviewer response for version 2

**DOI:** 10.21956/wellcomeopenres.23102.r75862

**Published:** 2024-04-01

**Authors:** Katelyn Esmonde

**Affiliations:** 1 Johns Hopkins Berman Institute of Bioethics, Johns Hopkins University, Baltimore, Maryland, USA

Thank you for the opportunity to read and review this manuscript. In this paper, the author puts forward a case for drawing on the concept of “expectations” in bioethics, as a tool to conceptualize the complexity of societal interactions. To help to illustrate the importance of “expectations,” the author draws on the initial handling of the COVID-19 pandemic in Vietnam as a case study. The author then looks to numerous fields of study to build a concept of “expectations,” and concludes by considering the implications of this concept for governance and future research.

 The concept of “expectations” put forward in this paper does seem like a useful and novel one for bioethics, and I applaud the work that the author has put in to think through the many complexities of the concept. I do think that the paper could make its points more clearly, and that the connection between the Vietnam COVID-19 case study and the concept of “expectations” could be strengthened. I also found some parts of the essay challenging to understand due to lengthy paragraphs and some very long sentences.

 First, like the other reviewers, I felt that the connection between the Vietnam case study and the gathering of various understandings of “expectations” could have been stronger. Given the title and the abstract, I expected that the paper would focus much more on public health ethics and COVID-19 in Vietnam; an interesting and important topic! However, most of the paper was not about that, and the threads were not carried through in the way they could be. As I was reading the Vietnam case study it wasn’t apparent to me why “expectations” was a useful device for framing the “possible human rights violations” that had occurred. Then, the paper shifts quite suddenly to a lengthy and detailed elaboration of “expectations,” with few references to Vietnam, COVID-19, or to public health law/ethics.

 I do think that moving the case study up in the paper was a good idea. However, the importance of the concept of “expectations” for understanding the COVID-19 response in Vietnam, and the response to the response, needs to be highlighted much more than it already is. Then, the author can make the following sections on “expectations” much more focused on public health law/ethics, drawing on the case study to provide more illustrative examples. I think that the paper is really promising, but more work is needed to connect these two parts of the paper.

 Second, section 4 (“Implications for governance and research agenda for the future”) is, at least right now, doing little to help readers truly understand the implications of “expectations.” Sentences like these are unwieldy and unclear: “The concept of expectations (which represents the totality of interactions of selves and social context) also implies that the law and governance of one jurisdiction can learn from others, but for them to be really effective, solutions must be designed, with considerations paid to and tailored to historical and social accounts for reason that underlying social and institutional arrangements are different expectations and these in turn yield different expectations.” (p. 14). Furthermore, with respect to future research and implications for governance, I would have liked for there to be more connections made between the concept of “expectations” and public health law/ethics. In general, the author could do more to connect this paper to bioethics.

 The other reviewers have given more detailed feedback on the author’s elaboration of the concept of “expectations.” I will add little to this, but I did want to say that this section, while very interesting and giving the reader much to think about, is quite lengthy and it was hard to come away with a clear understanding of what the author wanted to argue. I recognize that the author has tried to improve this with section 4, but I am still not feeling like I have a clear take-away regarding the importance of “expectations” for bioethics, or some take-aways regarding aspects of “expectations” that are particularly important for analysis.

 Finally, this is an incredibly small point, but on p. 10 the author wrote that “Stanley Milgram conducted two experiments.” He conducted many experiments, obviously, so the author should instead write that they will detail two experiments that are relevant to the concept of “expectations” in this paper.

Is the work clearly and accurately presented and does it cite the current literature?

Partly

If applicable, is the statistical analysis and its interpretation appropriate?

Not applicable

Are all the source data underlying the results available to ensure full reproducibility?

No source data required

Is the study design appropriate and is the work technically sound?

Yes

Are the conclusions drawn adequately supported by the results?

Partly

Are sufficient details of methods and analysis provided to allow replication by others?

Yes

Reviewer Expertise:

Public health ethics; sociology of health and illness; qualitative methods

I confirm that I have read this submission and believe that I have an appropriate level of expertise to confirm that it is of an acceptable scientific standard, however I have significant reservations, as outlined above.

# Response

**Published:** 2024-05-06

**Authors:** Hai Doan

**Affiliations:** University of Otago, New Zealand

I am thankful for your kind comments. Detailed revisions can be observed in the manuscript. Details of responses to reviewers’ comments and further explanations are provided hereunder.

**Reviewer Report Comments:**

 (Paragraph 3) : First, like the other reviewers, I felt that the connection between the Vietnam case study and the gathering of various understandings of “expectations” could have been stronger. Given the title and the abstract, I expected that the paper would focus much more on public health ethics and COVID-19 in Vietnam; an interesting and important topic! However, most of the paper was not about that, and the threads were not carried through in the way they could be. As I was reading the Vietnam case study it wasn’t apparent to me why “expectations” was a useful device for framing the “possible human rights violations” that had occurred. Then, the paper shifts quite suddenly to a lengthy and detailed elaboration of “expectations,” with few references to Vietnam, COVID-19, or to public health law/ethics.

 (Paragraph 4) :I do think that moving the case study up in the paper was a good idea. However, the importance of the concept of “expectations” for understanding the COVID-19 response in Vietnam, and the response to the response, needs to be highlighted much more than it already is. Then, the author can make the following sections on “expectations” much more focused on public health law/ethics, drawing on the case study to provide more illustrative examples. I think that the paper is really promising, but more work is needed to connect these two parts of the paper.

 (Paragraph 6) :The other reviewers have given more detailed feedback on the author’s elaboration of the concept of “expectations.” I will add little to this, but I did want to say that this section, while very interesting and giving the reader much to think about, is quite lengthy and it was hard to come away with a clear understanding of what the author wanted to argue. I recognize that the author has tried to improve this with section 4, but I am still not feeling like I have a clear take-away regarding the importance of “expectations” for bioethics, or some take-aways regarding aspects of “expectations” that are particularly important for analysis.

***Author Response:***

 >> Sections are now restructured. A new section 3.5. is constructed from certain materials of Section 2 (Cases). Now, Section 2 only provides cases and attempts to give rise to an initial impression of how expectations are relevant. Then, it comes to the section conceptualizing expectations (3.1 - 3.4). After that, the paper revisits Vietnamese cases described earlier to demonstrate and illustrate how expectations, as conceptualized, account for violations of human rights and certain related propositions within the Vietnamese context. This allows takeaways from sections 3.1-3.4 to be useful to shed light on patterns of behaviors. To concretize the points and provide a more comprehensive and comprehensive view and convincing argument, cases (legislation, case law, reports) in relation to COVID-19 in certain jurisdictions, namely, the UK, New Zealand, and Taiwan, are added. This reinforces one again the relevancy of expectations and demonstrates common expectations and expectations especially salient within the context of Vietnam. This also reinforces the conceptualization of expectation in earlier subsections. Then, building on the analysis of Section 3 (particularly Section 3.5), Section 4 comes out. The analysis is hermeneutic and back-and-forth. Hopefully, this is a better structure in terms of how sections link to each other, and the paper can progress with greater persuasiveness. I also hope that this can give a view as to the relevancy of and contributions to public health law and ethics of this paper.

 >> I also want to add that potentially there can be another way to address your concern related to the linkage between sections. In this possibility, I can, similarly, present cases first. Then, provide the conceptualization. Then, in each subheading of the conceptualization part, I can revisit an issue or a sub-issue or a case to illustrate the point. This is something I have done in the first revision (that can be referred back to in the version history of this paper). An advantage of this way is that the paper can be more concise, and it can be argued that the case links directly to the conceptualization.

 However, there are certain disadvantages some have been mentioned in your report:

 1) First, there shall not be enough space for demonstrating thoroughly how expectations relate to and account for Vietnamese cases and behaviours thereof (otherwise it can be a little bit distractive, i.e., as to the purpose of the section). Meanwhile, if what is to be shown is that Vietnamese cases are illustrative of each sub-heading of the conceptualization of expectations then ‘expectations within the context of Vietnam’ can only be shown scattered. Meanwhile, features of expectations should be observed as to have been drawn from the concept of expectations and analysis across areas (and where certain features have not yet been illustrated sufficiently by such areas, further examples are added) rather than Vietnamese cases; of course, Vietnamese cases standalone cannot provide sufficient ground for crystallizing these features or just demonstrating these features. Nor, I think, is it desirable to reconstruct the entire monument where other people have done some part of it.

 2) Second, in doing so, it presupposes without the benefit of analysis and substantiating that there were expectations underlying human rights violations and related propositions within the context of Vietnam. And if it is so presupposed, the possibility that I can deploy materials concerning public health law in relation to Covid-19 can be more limited. There shall also not be enough space to explore the landscape of legal ethical cases related to Covid-19.

 3) Third, if I do so, it can be argued that the takeaways of the conceptualization are not clear or are not applied to be of practical value. I guess – and me alone – that people with more theoretical/philosophical backgrounds can be pleased with a purely theoretical paper conceptualizing expectations and potential theoretical contributions. People having practical concerns, however, may want takeaways to be clearer and more applicable to Vietnamese cases.

 >>With the current approach, the conceptualization is clear, and takeaways from the conceptualization shall be used to argue how expectations relate to and account for Vietnamese cases. There are a conceptualization/theoretical part, an application part, and the ‘open-for-further-study’ part. Each part builds upon another but lends force from not only parts of this paper alone but also other studies. This allows me to construct a rounded and comprehensive paper (thus pleasing readers from a wide range of backgrounds in bioethics and health law), without falling into, as in the words of Dr Diego Silva “an ambitious task for anyone”.

 >>Meanwhile, if reviewers and readers have any concerns as to what specific materials or aspects should be included to make the paper more rounded and comprehensive, I am very happy to consider these recommendations subject to the scope of this paper (i.e., it cannot be too ambitious).  

**Reviewer Report Comments:**

 (Paragraph 5): Second, section 4 (“Implications for governance and research agenda for the future”) is, at least right now, doing little to help readers truly understand the implications of “expectations.” Sentences like these are unwieldy and unclear: “The concept of expectations (which represents the totality of interactions of selves and social context) also implies that the law and governance of one jurisdiction can learn from others, but for them to be really effective, solutions must be designed, with considerations paid to and tailored to historical and social accounts for reason that underlying social and institutional arrangements are different expectations and these in turn yield different expectations.” (p. 14).

***Author Response:***

 >> The sentence you mentioned is now restructured. In essence, it is talking about the desirability of critical comparative research. More comparative materials have been added to further clarify the point and provide a more comprehensive view. The entire section has also been revisited and revised. Certain components have been slimmed and streamlined. I hope this section now is with more brevity. I also hope that this section, as it is standing now, duly responds to and satisfies Dr Diego Silva’s and your concerns about the implications of expectations. Meanwhile, I pay great attention to keeping Section 4 builds upon and links with previous sections as well as within certain boundaries drawn by the thread of the paper. That is to keep the paper conceptually coherent.  

**Reviewer Report Comments:**

 (Continuation of Paragraph 5): Furthermore, with respect to future research and implications for governance, I would have liked for there to be more connections made between the concept of “expectations” and public health law/ethics. In general, the author could do more to connect this paper to bioethics.

***Author Response:***

   >> My apologies in advance if I have any misunderstanding. Section 3.1 in referring to the application of expectations across disciplines, briefly mentioned papers which suggest how expectations relate to biomedical ethics and health law. The concept is novel and any application is, however, limited. This is the reason why I added a footnote which read “Mark L Flear’s paper belongs to a Special Issue of the Journal of Law and the Biosciences which is about “advancing understanding of the way in which imaginaries underpin law’s engagement with biomedical technologies and science” (Mark L Flear and Richard Ashcroft, ‘Law, Biomedical Technoscience, and Imaginaries’ (2021) 8 Journal of Law and the Biosciences 1, 2.). It is introduced as “the first collection of law-led interdisciplinary accounts of imaginaries” ibid. Flear also suggested that the concept of ‘expectations’ has “obvious contemporary importance” for biomedical governance and regulation and legal and regulatory studies; however, “their roles receive little attention within legal and regulatory studies” Flear (n 21) 2. It can be observed from such assertions that ‘expectations’ is a relatively novel area of research in bioethics and health law.” In light of the structural and substantive revision concerning Vietnamese cases, the paper demonstrates how expectations account for certain violations and related propositions within the context of Vietnam, which is a prominent issue in public health ethics, public [health] law and governance (Section 3.5). And for that reason, the paper also demonstrates how expectations have connections with bioethics. The connections between expectations and ethics and law are demonstrated in Section 4. It has been intended that Section 4.2. is a final afterthought remark as to which issues in relation to the Vietnamese cases or broader issues of human rights violations can be benefited from certain kinds of further research which comes out in light of the application of expectations.

**Reviewer Report Comments:**

 (Paragraph 7): Finally, this is an incredibly small point, but on p. 10 the author wrote that “Stanley Milgram conducted two experiments.” He conducted many experiments, obviously, so the author should instead write that they will detail two experiments that are relevant to the concept of “expectations” in this paper.  

***Author Response:***

 >> Actually, because Wellcome Gateway encourages links to be added to help readers navigate information, I have added links to the newspaper and paper where readers can easily read descriptions of these two experiments. That is to prevent excessive descriptions that just quote the text of the papers. But in light of your comment, I guess it is desirable for some readers to read the descriptions while reading this paper as opposed to having to open other links (papers). Descriptions are added subsequently. It is really hoped that all concerns of reviewers are now satisfactorily addressed.

# Reviewer report: Reviewer response for version 1

**DOI:** 10.21956/wellcomeopenres.21034.r63336

**Published:** 2023-08-07

**Authors:** Max Steuer

**Affiliations:** 1 Jindal Global Law School, OP Jindal Global University, Sonipat, Haryana, India; 2 Department of Political Science, Comenius University in Bratislava, Bratislava, Slovakia

This manuscript tries to contribute both to studies understanding the changes in the standards of human rights protection in relation to the COVID-19 pandemic in various jurisdictions and to the frameworks of study of the behavior of political actors, the latter via a lens of how their 'expectations' might affect the standards of rights. This framing of the manuscript is an interesting one, though, as also pointed out by Reviewer 1, the goals so presented are very challenging to achieve in a single paper of standard length. 

 The focus on Vietnam is very welcome, as it is not frequently discussed in analyses of the COVID-19 pandemic and states of emergency--that being said, an explicit justification for why the authors think Vietnam is a suitable case study for the application of their conceptual framework (beyond the fact that it might be the jurisdiction they are most familiar with) could be useful. 

 Similarly, the interdisciplinary nature of the manuscript, bringing in insights from psychological, sociological and legal sources, is one of its strengths and helps avoid a schematic, rationality-premised study of the behavior of political actors.

 However, I think that there are some significant aspects that would need improvement or at least further engagement in the revision process. Firstly, I concur with Reviewer 1 that the limited linkage between theory and empirics considerably reduces the impact potential of this manuscript: Many subtypes of expectations and agents' reactions thereto introduced in Sections 3 and 4 remain without representations in the empirical section, which makes it less clear from where the authors derived them in the first place. At times, the framework is very complex and its presentation challenging to read (especially p. 8 of the pdf version of the article), so I wonder whether a more simplified framework with room for further research would not be more conducive to the argument and ultimately the impact of the manuscript. Despite this complexity, new categories are invoked in the empirical section (''expectations as presumptions that something may happen' versus 'expectations as desires to express powers’ - pp. 13-14) which are not in a dialogue with the conceptual/theoretical sections. 

 Secondly, it seems that the authors only attribute expectations to persons, as discussed in Sec. 3.2. (which covers not only objects, but also subjects of expectations in my reading). However, this is hardly the case, notably in the context of human rights, law and legal institutions which interpret it might also have expectations (and the authors' general definition of expectations mentioned in the abstract alludes to that as well). The authors may have a way how to address this within the scope of their conceptualization of expectations, but if so, it is not clear from the current version of the manuscript how this could be done. 

 Moreover, there are several claims without sufficient evidence:

- 'Scholars expect that resorting to emergency powers - or more precisely the declaration of resorting to emergency powers – could be a solution for human rights issues amidst the situation.' (p. 3) Not quite, there is a scholarly disagreement. For example, it manifested in competing opinions on whether states should use the derogation clause (Art. 15) of the European Convention on Human Rights (see e.g. Zysset 2022). One argument for (if the authors wish to engage with this debate, which I do not think is crucial for their argument) could be that states of emergency during actual emergencies help avoid a race to the bottom in the normal rights regime and, because they are expected to be temporary, they are preferable, as rights restrictions without the extraordinary regime may last much longer and become legitimized.  
- The authors seem to create a somewhat artificial unity between 'legal scholars' as an epistemic community when claiming that 'legal scholars attend to human rights issues' (p. 7). In fact, there may be other motivations of legal scholars, from whom many are not human rights specialists. 
- The allusion to bias of Ginsburg and Versteeg is interesting (p. 8), but not sufficiently elaborated upon in my view. If the authors wish to keep this point, it would be advisable to engage with the respective work in greater detail and show the readers how these bias operate. In particular, with Vietnam as a non-democratic regime, I am unclear on why the authors claim that scholars would have expectations of there being a system of the separation of powers (p. 12, last paragraphs of the subsection). Here, it is also unclear whether and how they differentiate between scholars from Vietnam who are working on domestic politics and law and do not publish in English, and international scholars who publish in English. Do their expectations differ? How do we know? 
- I do not think the authors conduct a 'frame analysis' as they claim in section 5.2.1. I think there is no need to introduce a new method that late into the manuscript. 
- '[G]ood health cannot exist in the absence of full guaranteeing of rights'--what does 'full guaranteeing of rights' mean (p. 14)? Is that even possible when the dominant contemporary accounts of rights tend to converge on conflicts between rights and hence the impossibility to 'fully guarantee' them are inevitable? 
- 'checks and balances mechanisms must effectively constrain the materialization of undue expectations' (p. 15) - this recommendation is not embedded in the findings of the manuscript or the literature. Thus, more justification would be needed for why it is being drawn in my view.

I also have a formal concern: the manuscript claims to have two co-authors but often the argument is presented in first-person singular ('I'). Since my background is in interdisciplinary democracy and socio-legal studies rather than bioethical and health studies, I might be used to a different standard in publications this regard, but to me, even if the other author is 'only' supervising and reviewing the writing, I would expect the arguments to be shared by both of them--otherwise, it is debatable why there is co-authorship instead of acknowledgement of feedback and support but without co-authorship. 

 Last but not least, there are a few interesting claims that would warrant further study, but perhaps cannot be drawn with the research design of the present one, namely:

- that political regimes show their 'true face' more during emergencies, particularly in case of non-democratic regimes which are built on propaganda that does not match the actual state of affairs (p. 12)
- that there is a tendency of lower-ranked officials to 'guess' the expectations of their superiors (p. 12)--on this, the work of Michael Mann (2004) on the operation of fascism might offer useful paralells. 

I wish the authors success in developing this manuscript and research agenda.

Is the work clearly and accurately presented and does it cite the current literature?

Partly

If applicable, is the statistical analysis and its interpretation appropriate?

Not applicable

Are all the source data underlying the results available to ensure full reproducibility?

No source data required

Is the study design appropriate and is the work technically sound?

Partly

Are the conclusions drawn adequately supported by the results?

Partly

Are sufficient details of methods and analysis provided to allow replication by others?

Partly

Reviewer Expertise:

My background is in interdisciplinary democracy research and socio-legal studies which includes Political Science and European studies. However, I have very limited familiarity with Vietnam as a jurisdiction and so I cannot assess any empirical claims made in this regard. Furthermore, I have not done work in bioethical and health law studies, so here I can only comment to the extent there are general claims about legal philosophy or legal studies, rather than any specifics focusing on health law and bioethics.

I confirm that I have read this submission and believe that I have an appropriate level of expertise to confirm that it is of an acceptable scientific standard, however I have significant reservations, as outlined above.

## References

[1] : To Derogate or to Restrict? The COVID-19 Pandemic, Proportionality and the Justificatory Gap in European Human Rights Law. *Jus Cogens* .2022;4(3) : 10.1007/s42439-022-00065-6 285-301 10.1007/s42439-022-00065-6

[2] : Fascists.2004; 10.1017/CBO9780511806568 10.1017/CBO9780511806568

# Response

**Published:** 2024-01-11

**Authors:** Hai Doan

**Affiliations:** University of Otago, New Zealand

This manuscript tries to contribute both to studies understanding the changes in the standards of human rights protection in relation to the COVID-19 pandemic in various jurisdictions and to the frameworks of study of the behavior of political actors, the latter via a lens of how their 'expectations' might affect the standards of rights. This framing of the manuscript is an interesting one, though, as also pointed out by Reviewer 1, the goals so presented are very challenging to achieve in a single paper of standard length. >> Thank you for your comment. We also perceive this paper as ambitious indeed. We agree with Dr Diego Silva that the issue can be solved or relieved if the paper is restructured with the cases come first which serve as background and evidence for the conceptualization part.   Similarly, the interdisciplinary nature of the manuscript, bringing in insights from psychological, sociological and legal sources, is one of its strengths and helps avoid a schematic, rationality-premised study of the behavior of political actors.   The focus on Vietnam is very welcome, as it is not frequently discussed in analyses of the COVID-19 pandemic and states of emergency--that being said, an explicit justification for why the authors think Vietnam is a suitable case study for the application of their conceptual framework (beyond the fact that it might be the jurisdiction they are most familiar with) could be useful.   >> We add in the Introduction, page 3 that “The rationale for which Vietnam is chosen …”       Firstly, I concur with Reviewer 1 that the limited linkage between theory and empirics considerably reduces the impact potential of this manuscript: Many subtypes of expectations and agents' reactions thereto introduced in Sections 3 and 4 remain without representations in the empirical section, which makes it less clear from where the authors derived them in the first place. >>> Currently, the paper has been restructured. The paper hopes that the restructure indicates clear impressions that many subtypes of expectations and agents' reactions can be illustrated by studies across disciplines and Vietnamese cases.   At times, the framework is very complex and its presentation challenging to read (especially p. 8 of the pdf version of the article), so I wonder whether a more simplified framework with room for further research would not be more conducive to the argument and ultimately the impact of the manuscript. >> A new section 4 on “Implications for governance and research agenda for the future” is added. This paper hopes that this new section can give a sense of how the concept and its framework can have implications for governance and further research.   Despite this complexity, new categories are invoked in the empirical section (''expectations as presumptions that something may happen' versus 'expectations as desires to express powers’ - pp. 13-14) which are not in a dialogue with the conceptual/theoretical sections.   >>> Initially, this paper mentioned these phenomena just to suggest that they can be specific possible forms of expectations (rather than new categories). Currently, the paper has been restructured. With the hope to make the paper clearer and more concise and better justified, cases are used to illustrate the conceptualization and put immediately after the conceptualization as illustrations; they are also reduced in length.   Secondly, it seems that the authors only attribute expectations to persons, as discussed in Sec. 3.2. (which covers not only objects, but also subjects of expectations in my reading). However, this is hardly the case, notably in the context of human rights, law and legal institutions which interpret it might also have expectations (and the authors' general definition of expectations mentioned in the abstract alludes to that as well). The authors may have a way how to address this within the scope of their conceptualization of expectations, but if so, it is not clear from the current version of the manuscript how this could be done.   >> Thank you very much for your reminder on the points that we have missed. Two categories are added in relation to this matter (see subsection 3.2). It does not seem to us that adding these two categories is difficult because these have been addressed by the body of literature of legal studies. Thus, we can add them by introducing the relevant body of law (and the introduced accounts of legal studies hopefully can support these categories) (see subsection 3.1).     Moreover, there are several claims without sufficient evidence: 'Scholars expect that resorting to emergency powers - or more precisely the declaration of resorting to emergency powers – could be a solution for human rights issues amidst the situation.' (p. 3) Not quite, there is a scholarly disagreement. For example, it manifested in competing opinions on whether states should use the derogation clause (Art. 15) of the European Convention on Human Rights (see e.g. Zysset 2022). One argument for (if the authors wish to engage with this debate, which I do not think is crucial for their argument) could be that states of emergency during actual emergencies help avoid a race to the bottom in the normal rights regime and, because they are expected to be temporary, they are preferable, as rights restrictions without the extraordinary regime may last much longer and become legitimized.    The authors seem to create a somewhat artificial unity between 'legal scholars' as an epistemic community when claiming that 'legal scholars attend to human rights issues' (p. 7). In fact, there may be other motivations of legal scholars, from whom many are not human rights specialists.   >> Apologies for leaving any impression, as you mentioned, because it is not what this paper intended.  What was intended is to suggest various possible expectations. Hopefully, the rephrasing and restructuring now can not only make the paper more concise but also shape the points the paper aims to convey clearly.   The allusion to bias of Ginsburg and Versteeg is interesting (p. 8), but not sufficiently elaborated upon in my view. If the authors wish to keep this point, it would be advisable to engage with the respective work in greater detail and show the readers how these bias operate. In particular, with Vietnam as a non-democratic regime, I am unclear on why the authors claim that scholars would have expectations of there being a system of the separation of powers (p. 12, last paragraphs of the subsection). Here, it is also unclear whether and how they differentiate between scholars from Vietnam who are working on domestic politics and law and do not publish in English, and international scholars who publish in English. Do their expectations differ? How do we know? >> Thank you very much for the great point. The social context of the US that might give rise to the expectations of Ginsburg and Versteeg is explained clearer and juxtaposed with the Vietnamese context. The context of Vietnam explains expectations for a system of better checking of powers (though as this paper suggested, these expectations might or might not be rational). See section 3.3.5. Your other point is great but this paper cannot address it because it is not aware of the writings of international scholars who publish in English on Vietnamese emergency. Yet, it seems that this paper can still deliver what it aims to deliver without addressing this point. It looks for your understanding.     I do not think the authors conduct a 'frame analysis' as they claim in section 5.2.1. I think there is no need to introduce a new method that late into the manuscript. >> This paper does not aim to 'frame analysis' or to introduce a new method there. What was intended is to suggest various possible expectations. The point has been reduced and put into section 3.3.5. as a demonstration to keep the paper concise and clear.   '[G]ood health cannot exist in the absence of full guaranteeing of rights'--what does 'full guaranteeing of rights' mean (p. 14)? Is that even possible when the dominant contemporary accounts of rights tend to converge on conflicts between rights and hence the impossibility to 'fully guarantee' them are inevitable?  >> Thank you for this (another) great point. This is truly a point that needs further explored and discussed normatively and openly. However, the paper is currently not in a position to address this point. Meanwhile, to keep the paper concise, this matter has been cut out from the paper.   'checks and balances mechanisms must effectively constrain the materialization of undue expectations' (p. 15) - this recommendation is not embedded in the findings of the manuscript or the literature. Thus, more justification would be needed for why it is being drawn in my view. >> This matter has been cut out from the text because as you see, the specific recommendation does not go well along with the general conceptualization design of this paper.   Last but not least, there are a few interesting claims that would warrant further study, but perhaps cannot be drawn with the research design of the present one, namely: that political regimes show their 'true face' more during emergencies, particularly in case of non-democratic regimes which are built on propaganda that does not match the actual state of affairs (p. 12)  that there is a tendency of lower-ranked officials to 'guess' the expectations of their superiors (p. 12)--on this, the work of Michael Mann (2004) on the operation of fascism might offer useful paralells. >> This piece of advice is really helpful and interesting. However, this paper does not have the capacity to address these issues (in light of its research design). We thus see this advice as suggestions for future research. That is addressed in the new section 4. However, we also cannot introduce directly and in detail the book Fascists (by Michael Mann (2004)) because it does not go along very well with the flow of ideas of the paper (that can surprise some readers perhaps).

# Response

**Published:** 2024-01-11

**Authors:** Hai Doan

**Affiliations:** University of Otago, New Zealand

Dear Dr Diego Silva and Dr Max Steuer, Thank you very much for your time spent reading our MS and very helpful comments. Apologies for the delayed revision and response because of the confusion and misunderstanding that we have to wait for the third reviewer’ report before carrying out the revision. We hope that the revisions and responses here address your concerns.

# Reviewer report: Reviewer response for version 1

**DOI:** 10.21956/wellcomeopenres.21034.r57553

**Published:** 2023-06-22

**Authors:** Diego Silva

**Affiliations:** 1 Sydney Health Ethics, The University of Sydney, Sydney, New South Wales, Australia

Thank you to the authors for the opportunity to review their paper. The essay claims that getting clear on the normative sense of 'expectations' that policy makers or decision makers use can be "fruitful concept for bioethical and health law studies because it helps better understand the complexity of interactions in society and patterns of thinking and acting of actors in real-world contexts, which have implications for how human rights and other ethical norms can be better realized in practice".

 It's an interesting and original idea but there are problems with the essay fulfilling its thesis. Part of the issue is claims are made but not defended in sufficient depth. One reason this might be the case is that the paper is trying to do two things: first, provide a conceptual argument about how expectations should be understood and why it's an important concept for bioethics, and second, then applying it to the context of COVID19 in Vietnam. That's an ambitious ask for anyone and I'm not sure whether it would have been better to do the conceptual work more fulsomely first and then in another paper the applied part. 

 With regard to the conceptual work, section two and three provides various definitions of 'expectations' from various fields as well as dictionary definitions. But there's insufficient analysis of these various definitions and their implications for ethics (e.g., the authors seem to value Keynes' definition but that's implied and could used a more robust defence). In section three, the authors reach their particular understanding of expectation, but then there's a lack of an explanation of it and a defence of it as a concept (end of section 3.1).

 The idea of complexity is introduced but not explored despite it being a key idea that motivates the paper in the first place. What do the authors mean by complexity and how does a normative understanding of expectation resolve some of the challenges we see in complexity of human behaviour vis-a-vis COVID19 decision-making and values? 

 Regarding the discussion of vagueness in section 3.2 and onward, I kept asking myself "okay but why does this matter?" I think it may very well matter and that the authors are onto something important, but the "this is why this matters" could be made more explicit. 

 Also, a minor point that will be a big deal to some, perhaps warranted: there's a sliding back and forth between human rights and ethics when the relationship between the two has been contested in the literature for a while now. 

 Again, I think there's something in this paper that is unique or at least not often discussed, but I don't think the authors have met their own aims. Perhaps one way forward is to actually take the definition at the end of section 3.1 and start there and go backward with what's in section two. That is, use what's in section two to fill out the definition where needed but always with an eye toward making clear why it matters that we care about 'expectations'. Maybe even consider starting with the case example of Vietnam and then coming back to it in the end to illustrate your conceptual points?

Is the work clearly and accurately presented and does it cite the current literature?

Yes

If applicable, is the statistical analysis and its interpretation appropriate?

Not applicable

Are all the source data underlying the results available to ensure full reproducibility?

No source data required

Is the study design appropriate and is the work technically sound?

Yes

Are the conclusions drawn adequately supported by the results?

Yes

Are sufficient details of methods and analysis provided to allow replication by others?

Yes

Reviewer Expertise:

Public health ethics; infectious disease ethics; normative ethics; political theory.

I confirm that I have read this submission and believe that I have an appropriate level of expertise to confirm that it is of an acceptable scientific standard, however I have significant reservations, as outlined above.

# Response

**Published:** 2024-01-11

**Authors:** Hai Doan

**Affiliations:** University of Otago, New Zealand

Dear Dr Diego Silva and Dr Max Steuer, Thank you very much for your time spent reading our MS and very helpful comments. Apologies for the delayed revision and response because of the confusion and misunderstanding that we have to wait for the third reviewer’ report before carrying out the revision. We hope that the revisions and responses here address your concerns.   It's an interesting and original idea but there are problems with the essay fulfilling its thesis. Part of the issue is claims are made but not defended in sufficient depth. One reason this might be the case is that the paper is trying to do two things: first, provide a conceptual argument about how expectations should be understood and why it's an important concept for bioethics, and second, then applying it to the context of COVID19 in Vietnam. That's an ambitious task for anyone and I'm not sure whether it would have been better to do the conceptual work more fulsomely first and then in another paper the applied part. Perhaps one way forward is to actually take the definition at the end of section 3.1 and start there and go backward with what's in section two. That is, use what's in section two to fill out the definition where needed but always with an eye toward making clear why it matters that we care about 'expectations'. Maybe even consider starting with the case example of Vietnam and then coming back to it in the end to illustrate your conceptual points?   >> Thank you for your comment. We also perceive this paper as ambitious indeed. Your comments are helpful and we agree with your points. We think that the issue you mentioned can be solved or relieved if the paper is restructured. The cases are now being presented earlier and analysis goes hand in hand with the conceptualization as demonstrations.   With regard to the conceptual work, section two and three provides various definitions of 'expectations' from various fields as well as dictionary definitions. But there's insufficient analysis of these various definitions and their implications for ethics (e.g., the authors seem to value Keynes' definition but that's implied and could used a more robust defence). In section three, the authors reach their particular understanding of expectation, but then there's a lack of an explanation of it and a defence of it as a concept (end of section 3.1). >>> It seems that your concern here can also be addressed with the restructure. Our apologies for leaving an impression that we value Keynes' definition. We value all understandings of expectations equally and without prejudice. We see that different understandings touch the concept of expectations from different angles although there are commonalities (that suggest the essence of the concept – our conceptualization of expectations). These accounts can supplement each other. We elaborate more on accounts of other disciplines (psychology, sociology, law) to reduce the possible impression that we value Keynes’s understanding. We neither aim to defend nor modify these existing understandings as they have been defended and empirically tested somewhere. Rather, these understandings should be seen as *illustrations and evidence* of our conceptualization and elaborate on the conceptualization with respect to the features and operations of expectations ( *to justify our conceptualization*). Notwithstanding, you can obverse that Vietnamese and other empirical cases reinforce these accounts to an extent.   We also add explanations in the introduction and section 3 to make clear our intention in this regard.   “This paper conceptualizes ‘expectations’ and indicates some implications of this concept. The conceptualization is done by the analytical method of interpreting meanings provided by the dictionaries, gathering relevant accounts across disciplines and using cases, particularly Vietnam’s response during the COVID-19 pandemic as an illustration.” “Implications for ethics and law are suggested in section 4.   The idea of complexity is introduced but not explored despite it being a key idea that motivates the paper in the first place. What do the authors mean by complexity and how does a normative understanding of expectation resolve some of the challenges we see in complexity of human behaviour vis-a-vis COVID19 decision-making and values? >> What is meant by ‘complexity’ is the interactions of social, historical, and agency. This is further illustrated in the conceptualization section (via Vietnamese cases) as well as the new section 4.   Regarding the discussion of vagueness in section 3.2 and onward, I kept asking myself "okay but why does this matter?" I think it may very well matter and that the authors are onto something important, but the "this is why this matters" could be made more explicit. >> A new section 4 on “Implications for governance and research agenda for the future” is added.     Also, a minor point that will be a big deal to some, perhaps warranted: there's a sliding back and forth between human rights and ethics when the relationship between the two has been contested in the literature for a while now. >> For clarity, we add “this paper deploys phrases ethics and law and/or rights together to signify that they are not synonymous but should be simultaneously considered in relation to a particular issue”.
